# New insights into the epidemiology of *Listeria monocytogenes* – A cross-sectoral retrospective genomic analysis in the Netherlands (2010–2020)

**DOI:** 10.3389/fmicb.2023.1147137

**Published:** 2023-04-06

**Authors:** Claudia E. Coipan, Ingrid H. M. Friesema, Angela H. A. M. van Hoek, Tijs van den Bosch, Maaike van den Beld, Sjoerd Kuiling, Lapo Mughini Gras, Indra Bergval, Thijs Bosch, Bart Wullings, Menno van der Voort, Eelco Franz

**Affiliations:** ^1^Centre for Infectious Disease Control, National Institute for Public Health and the Environment (RIVM), Bilthoven, Netherlands; ^2^Wageningen Food Safety Research (WFSR), Wageningen, Netherlands; ^3^Institute for Risk Assessment Sciences (IURAS), Utrecht University, Utrecht, Netherlands

**Keywords:** Listeria, human, food, source, cluster, persistence, cgMLST

## Abstract

**Introduction:**

Listeriosis, caused by infection with *Listeria monocytogenes* (*Lm*), is a relatively rare but severe disease with one of the highest mortality rates among bacterial foodborne illnesses. A better understanding on the degree of *Lm* clustering, the temporal distribution of the clusters, and their association with the various food sources is expected to lead to improved source tracing and risk-based sampling.

**Methods:**

We investigated the genomic epidemiology of *Lm* in the Netherlands between 2010 and 2020 by analyzing whole-genome-sequencing (WGS) data of isolates from listerioss patients and food sources from nationwide integrated surveillance and monitoring. WGS data of 756 patient and 770 food/environmental isolates was assessed using core-genome multi-locus sequence typing (cgMLST) with Hamming distance as measure for pairwise distances. Associations of genotype with the epidemiological variables such as patient’s age and gender, and systematic use of specific drugs were tested by multinomial logistic regressions. Genetic differentiation of the *Lm* within and between food categories was calculated based on allele frequencies at the 1701 cgMLST loci in each food category.

**Results:**

We confirmed previous results that some clonal complexes (CCs) are overrepresented among clinical isolates but could not identify any epidemiological risk factors. The main findings of this study include the observation of a very weak attribution of *Lm* types to food categories and a much better attribution to the producer level. In addition, we identified a high degree of temporal persistence of food, patient and mixed clusters, with more than half of the clusters spanning over more than 1 year and up to 10  years.

**Discussion:**

Taken together this would indicate that identifying persistent contamination in food production settings, and producers that process a wide variety of raw food produce, could significantly contribute to lowering the *Lm* disease burden.

## 1. Introduction

Invasive infection with *Listeria monocytogenes* (*Lm*) is a relatively rare but severe foodborne zoonosis with the most hospitalisations and one of the highest mortality rates among bacterial foodborne pathogens (EFSA and ECDC, 2022). *Lm* is widespread in the environment and has the ability to persist in food processing facilities from where it can contaminate food products (Ferreira et al., 2014), with food consumption being considered the main route of transmission of *Lm* to humans (Buchanan et al., 2017). Clinically, next to gastroenteritis, listeriosis may lead to septicaemia, encephalitis, meningitis, abortion, still-births, and/or neonatal infections (Drevets and Bronze, 2008; Koopmans et al., 2013). Illness mainly occurs among the elderly, immunocompromised persons, neonates, and pregnant women (Friesema et al., 2015; Pohl et al., 2019). The incidence of listeriosis is increasing in the Netherlands and the European Union, despite *Lm* rarely exceeding the food safety microbiological criteria (as set in Commission Regulation EC 2073/2005) in tested ready-to-eat food products (EFSA and ECDC, 2022).

Some characteristics of *Lm*, such as the capacity to replicate at low temperatures and to persist on food-processing surfaces under adverse conditions, increase the risk of contamination during the production of food products that are typically pre-packaged and ready-to-eat (Hurley et al., 2019). Together with the severity of clinically overt listeriosis, this implies the need for a high quality surveillance system, including both epidemiological and microbiological data collection and analysis. However, the limited discriminatory power of the traditional molecular typing methods like pulsed-field gel electrophoresis (PFGE) in combination with the often small numbers of clustered patients observed, and the frequent occurrence of *Lm* in common foods, has hindered the successful identification of specific food products or food business operators (FBOs) as sources of human infection (van Walle et al., 2018). With the advances in DNA sequencing technologies, whole-genome-sequencing (WGS) has become the standard method for routine surveillance with high discriminatory power among isolates (Franz et al., 2016). For *Lm* this has led to the insight that clusters are more common than anticipated and can persist for several years (van Walle et al., 2018). Identification and subsequent source tracing of these persistent clusters, coupled with source attribution of sporadic cases, may be a way forward in reducing the disease burden of *Lm*.

In the Netherlands, WGS and core-genome MLST (cgMLST) were implemented as routine tools for epidemiological surveillance of *Lm* in 2017 and retrospective sequencing has been applied to isolates from 2010 onwards in order to add historical context to the contemporary surveillance. Additionally, since 2017, food and animal *Lm* isolates are being sequenced and shared by the competent authorities. This provided the opportunity to conduct cross-sectoral integrated WGS-based surveillance for cluster detection and outbreak investigation. In this study we investigated the genomic epidemiology of *Lm* in the Netherlands between 2010 and 2020 by analyzing isolates from listeriosis patients and food sources from nationwide surveillance and monitoring. A better understanding on the degree of *Lm* clustering, the temporal distribution of the clusters, and their association with the various food sources is expected to lead to improved source tracing, risk-based sampling, and intervention strategies.

## 2. Materials and methods

### 2.1. Terminology

Throughout the manuscript we use specific terms to refer to the units of our analyses. When referring to the symptomatic listeriosis patients we use the term ***cases***. Of these cases, as well as from the food products and FBO environments, ***samples*** were taken. These samples were further cultured *in vitro* to obtain bacterial ***isolates***. These isolates were subjected to WGS, and the resulting raw reads were assembled into assemblies, which we refer to as ***sequences***. Thus, in this study, the ***cases*** were the object of the epidemiological analysis, while the ***sequences*** were the object of the genomic analyses. The other terms are used according to the context.

### 2.2. Surveillance

Listeriosis is a notifiable disease in the Netherlands since December 2008. Laboratory confirmed cases have to be reported to the regional public health service, which contacts the patient or relatives with a short questionnaire about underlying (chronic) diseases and drug use, clinical course of listeriosis, and exposure to possible risk factors in the 30 days before disease onset. This information, together with patient characteristics, is then reported in the national, web-based infectious disease notification database. A mother and her newborn with listeriosis are notified separately, but with reference to each other and are considered as one case in the analyses. Clinical microbiological laboratories send *Lm* isolates from cases with invasive disease to the Netherlands Reference Laboratory for Bacterial Meningitis (NRLBM), which forwards the isolates to the National Institute for Public Health and the Environment (RIVM) for further typing for national infectious disease surveillance purposes. The trend in the number of reported cases of listeriosis was tested with a generalized linear model of the Poisson family and log link function, where the total population of the Netherlands was used as offset.

Food sampling within the Netherlands occurred routinely, both randomly as well as risk-based, by the Netherlands Food and Consumer Product Safety Authority (NVWA). Food samples were delivered to Wageningen Food Safety Research (WFSR) and tested under accreditation using methods equivalent to ISO 11290-1 or 11290–2. *Lm* isolates that were found in positive food samples were shortly stored cryogenically until DNA extraction. For further typing one colony per food isolate, and a smear of colonies per human isolate was used.

The dataset used in this study covers the years 2010–2020 for the human surveillance and 2015–2020 for the food surveillance.

### 2.3. PFGE typing

Up to 2016, isolates were genotypically characterized by restriction enzyme analysis with *AscI* and *ApaI*, followed by PFGE using a PulseNet standardized protocol (Standard operating procedure for PulseNet PFGE of *Listeria monocytogenes*, 2017). Banding patterns were compared within BioNumerics (Applied Maths, Sint-Martens-Latem, Belgium).

### 2.4. Whole genome sequencing

Since, 2017 WGS has become the standard typing method for the national surveillance of human listeriosis and monitoring of *Lm* in foodstuff. Patient *Lm* isolates of 2016 were retrospectively sequenced within RIVM and those of 2010–2015 were sequenced within the ELiTE-study (European Listeria Typing Exercise Extension to Whole Genome Sequencing) led by the European Centre for Disease Prevention and Control (ECDC; van Walle et al., 2018). For both the human and food isolates, sequencing from 2016 to 2019 was performed by a commercial sequencing company, using Illumina HiSeq (2×100 bp) and Illumina NovaSeq (2×150 bp) and from 2020 onwards by RIVM, using Illumina NextSeq (2×150 bp). All sequences were subjected to quality control and *de novo* assembled using an in-house developed pipeline (Van Wijk, 2018). Raw data with phred score > 30 and draft genomes with a total length of 2,700,00 to 3,230,000 bp, N50 > 10,000 bp, GC% of 37.6 to 38.2%, and an average read coverage ≥10 were used in further analysis.

Assessment of the 7 loci multilocus sequence typing (MLST) (Ragon et al., 2008), and core genome MLST (cgMLST; schema developed by Ruppitsch et al., 2015), as well as determination of sequence type (ST) and clonal complex [CC; as defined in Ragon et al. (2008)] was done in Ridom SeqSphere+ version 5.0.0 (Ridom GmbH, Münster, Germany). All assembled genome sequences that had 98.1–100% of the loci identified (i.e., < 33 loci missing), were considered to have a good quality and were used in subsequent analyses.

Pairwise distances between sequences were measured as Hamming distances using all cgMLST loci. Clusters of sequences based on cgMLST were defined based on single-linkage hierarchical clustering, with a threshold of maximum seven allelic differences over the 1701 cgMLST loci (Ruppitsch et al., 2015). The resulting clusters are equivalent to the cgMLST type described in the literature but not identical, as we have calculated the clusters independently of any public database. As the CC is one of the most widely used typing level to report on population structure of pathogens, we refer to the clusters by using a combined notation of CC and cluster: {CC label}_{cluster label} (e.g., CC1_613).

Persistence of clusters was defined as presence of sequences belonging to a cluster over a time span of more than 1 year, regardless of the continuity. Genetic differentiation of the *Lm* within and between food categories was calculated based on allele frequencies at the 1701 cgMLST loci in each population (food category), using the functions implemented in the R package *hierfstat* v 0.5–10 (Goudet and Jombart, 2020). For an empirical source attribution, the sequences of human *Lm* that did not belong to clusters for which a clear food source could be identified were grouped to particular food produce based on the minimum pairwise Hamming distances.

### 2.5. Comparison cgMLST–PFGE

cgMLST and PFGE typing results were compared by the Fowlkes-Mallows (FM) index (Fowlkes and Mallows, 1983), as implemented in the R package *profdpm* v 3.3 (Shotwell et al., 2013). We compared the concordance for the pulsotypes obtained with *ApaI* (95% similarity), *AscI* (85% similarity), and the internationally accepted combination of the two enzymes (*ApaI*/*AscI*), and for a variable allele difference threshold in the cgMLST (from one to 50). The direct correspondence of clusters defined by either PFGE or cgMLST was visualized in the form of summary statistics on the cluster size. Persistence of clusters and correspondence of the cgMLST and PFGE clusters were visualized using the R package *alluvial* v 0.1–2 (Bojanowski and Edwards, 2016).

### 2.6. Genotype association with epidemiological variables and source of isolation

Associations of genotypes with epidemiological variables such as patient’s age or gender, and systematic use of specific drugs were tested by multinomial logistic regressions as implemented in the R package *nnet* v 7.3–15 (Venables and Ripley, 2002). The genotype was tested as nominal outcome variable, and the log odds of the outcome was modeled as a linear combination of the predictor epidemiological variables. We did not test for associations of genotype and risk factors with the symptoms developed by the patients as these latter ones were incomplete and not reliable enough to be used in statistical tests. Our list of symptoms corresponded to the moment of sampling and it was not updated during sickbed; it is, thus, possible that patients have developed additional symptoms after the sampling moment that were not reported. Furthermore, the symptoms of bacterial infections could be the expression of both host factors, as well as bacterial factors.

To account for the uncertainty generated by variable sample sizes, we have estimated the distribution of the various genotypes in each food category by generating 10,000 random samples from the observed Dirichlet distributions. Should one assume that the prevalence of the various genotypes among the human *Lm* sequences are a direct reflection of the prevalences among the food *Lm* sequences, then the distributions of the genotypes for the various sources should be overlapping. To gain further insight into potential specific niches of the various *Lm* types, we have compared the distributions of the types between the products from livestock aggregated into dairy, fresh and processed meat, where processed meat was defined as “any meat that has been modified in order to either improve its taste or extend its shelf life.” Similarly, we have compared the distributions of types among the fish/shellfish products (eel, herring, mackerel, salmon, trout, lobster, mussels, and shrimp).

Additionally, associations between the genotype (i.e., lineage, serogroup, clonal complex, cluster) and the origin of the isolates, i.e., human or food source or category thereof, were tested by means of Fisher’s exact test (Fisher, 1992), with false discovery rate (FDR) (Benjamini and Hochberg, 1995) correction of the value of *p*-values The tests yielding a value of *p* <=0.05 were considered significant.

## 3. Results

### 3.1. Surveillance

Between 2010 and 2020, 989 cases of listeriosis were reported in the Netherlands. Overall, a slight increase in the number of reported cases of listeriosis has been observed from 2010 to 2020 (intercept *p* = 0.003), with considerable yearly fluctuations (between 72 and 117 cases per year; *p* = 0.02). The 50 materno-neonatal cases represented 5.1% of the cases with annually one to nine cases and an annual incidence of 0.03 (range 0.01-0.05) cases per 100,000 inhabitants. Two pregnancies concerned twins. The outcome of the pregnancies or of severely ill neonates was not always reported, but in at least 12 pregnancies (24%) stillbirth/neonatal death was reported and five miscarriages (10%) occurred. The majority of the cases were non-materno-neonatal cases with 69 to 113 cases per year, representing an annual incidence of 0.5 (range 0.41–0.66) per 100,000 inhabitants. The age of the non-materno-neonatal cases varied between one and 98 years (median 74 years). The incidence of listeriosis increased with age, and was highest in the age group 65+ (413/989, 41.8%). More men than women were infected with *Lm* (566/989, 57.2% vs. 421/989, 42.6%). Moreover, men above 65 years had an odds ratio (OR) of 1.8 for *Lm* infection compared to women in the same age group (*p* < 0.001).

Thirteen per cent of the non-materno-neonatal listeriosis cases died due to the infection, the youngest being 35 years and the oldest 98 years (median 77 years). The outcome of the disease remained unknown for 8% of the cases.

About one third of the clinical cases was reported to be under medication with antacids (379/989; 38.3%), and a slightly higher percentage used immunosuppressives (412/989; 41.6%); among these there were also users of both medicine categories (208/989; 21%).

**Figure 1:**
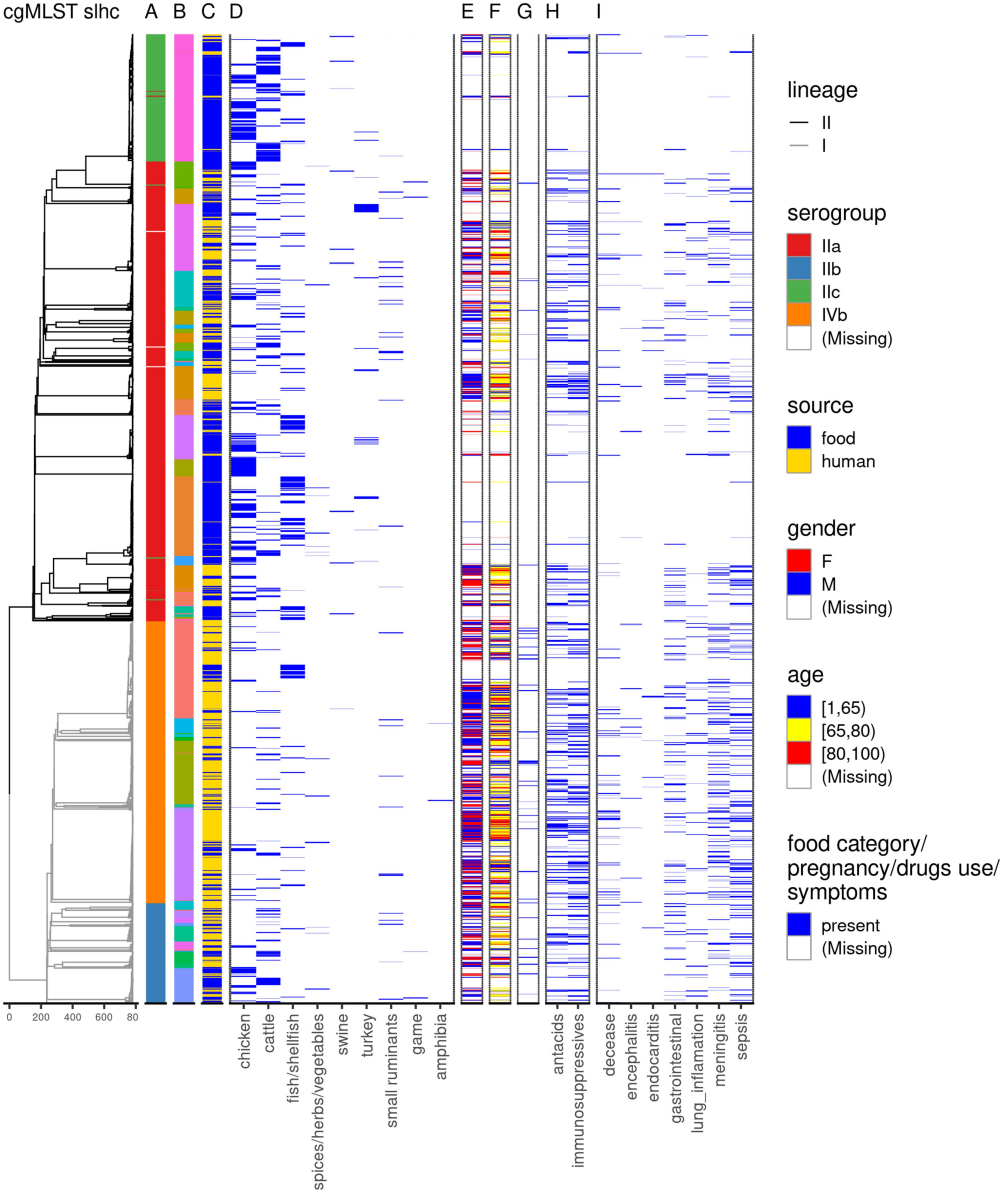
Overview of the dataset used in this study. The dendrogram is based on single linkage hierarchical clustering of cgMLST profiles of the isolates; the branches are colored by lineage. The annotation columns indicate the serogroup **(A)**, clonal complex–due to the large number of clonal complexes, these are not displayed in the legend, but can be found in Supplementary Table 1 **(B)**, source of the *Lm* sequences **(C)**, and food category **(D)**. For the human cases data on gender **(E)**, age **(F)**, pregnancy **(G)**, medicine use **(H)**, and symptoms following the *Lm* infection **(I)** is depicted.

Gastrointestinal symptoms were observed in 17.3% of the cases (171/989), while disseminated symptoms were observed in 50.6% of the cases (500/989), with 3.2% of the cases (32/989) presenting both gastrointestinal and disseminated symptoms. Among the disseminated symptoms, the most frequent one was sepsis (249/989; 25.2%), followed by meningitis (207/989; 20.9%), and pneumonia (86/989; 8.7%), with encephalitis (27/989; 2.7%) and endocarditis (13/989; 1.3%) being the least frequent (Figure 1; Supplementary Table 1).

### 3.2. Sequences

Isolates were received for 756 of the 989 human cases (76.4%). In one case, two different *Lm* strains were detected, and both sequences were included. Another 770 sequences were obtained from *Lm* isolates from food products and animal feces (n = 19) as part of the routine monitoring conducted by NVWA. Because the majority of non-human *Lm* sequences was represented by food products (95.2%) throughout the manuscript we will refer generically to the non-human *Lm* sequences as food *Lm* sequences. From 768 (99.7%) food *Lm* sequences the product category was known. Most of these sequences were collected from chicken products (39%), followed by cattle products (27.1%), and fish/shellfish (21%; Figure 1; Supplementary Table 1).

### 3.3. Limited concordance of cgMLST and PFGE

PFGE profiles were available for 418 human isolates over the years 2010–2016. Based on *ApaI*, there were 187 clusters, at 95% similarity, *AscI* could discern 95 clusters at 85% similarity, and the combination of the two enzymes discerned 74 clusters with a maximum cluster size of seven sequences, and 211 singletons (Supplementary Figure 1). Based on cgMLST, 54 clusters and 236 singletons were distinguished. The largest cluster contained 23 sequences (Supplementary Figure 1). Although the number of clusters in PFGE *ApaI*-*AscI* and cgMLST was comparable, the FM index was only 0.2, indicating a rather poor concordance of the two typing methods. A slightly higher concordance was observed with cgMLST clusters defined with a higher distance threshold, i.e., 0.24 for a distance of 12 alleles (Supplementary Figure 2). The majority of the larger clusters defined by either of the two methods did not have a corresponding cluster in the other method (Supplementary Figure 1). Occasionally several isolates would remain part of the same cluster in both methods, but it would never exceed four isolates (Supplementary Figure 1). The best concordance was observed between cgMLST and the pulsotypes defined by *AscI* only, with an FM index of 0.35 for a threshold of seven cgMLST alleles, and an FM index of 0.48 for a threshold of 36 cgMLST alleles (Supplementary Figure 2). This implies that cgMLST would be more consistent with PFGE if the pulsotypes were defined based on 85% similarity in the restriction profiles with only *AscI* enzyme.

### 3.4. Population structure at various typing levels

#### 3.4.1. Lineages

The distribution across lineages was 39.4% (602/1526) for lineage I and 60.6% (924/1526) for lineage II (Supplementary Table 1). However, that distribution was not similar among the human and food *Lm* sequences; lineage I was overrepresented among the human *Lm* sequences (OR = 6.1, Fisher’s exact test *p* < 0.0001). Lineage II was more prevalent among most of the food sources. In terms of prevalence of lineages among the food sources, the only significant difference was observed for cattle (OR = 1.8, *p* < 0.01) and chicken (OR = 0.5, *p* < 0.01). Lineage I was overrepresented in dairy products and lineage II in fresh meat. Among the fish/shellfish products lineage I was associated with trout and lineage II with salmon (Supplementary Table 2).

#### 3.4.2. Serogroups

For 1,523 (99.8%) sequences the serogroup was determined. The dominant serogroup was IIa, (47.1%), followed by IVb (29.1%), IIc (13.3%), and IIb (10.4%) (Figure 1; Supplementary Table 1). The largest part of serogroups IIa and IIc was represented by food *Lm* sequences (60.7, and 90.1%, respectively; Figure 1; Supplementary Table 3). The average proportion of serogroup IIa was significantly higher among food *Lm* sequences (56.5%) compared to the human *Lm* sequences (37.3%), as was the case for serogroup IIc (23.8% in food vs. 2.8% in human). The opposite was observed for serogroup IVb, which was much more prevalent among the human *Lm* sequences (47.5%) than among the food *Lm* sequences (11.1%; Supplementary Table 4). No significant differences were observed for serogroup IIb between the two sources (8.5% in food vs. 12.4% in human; Supplementary Table 5). Among the food *Lm* sequences the turkey products were overrepresented in serogroup IIa compared to other food categories. Similarly, cattle products were more abundant among serogroups IIc, and IIb, and fish/shellfish products were more abundant among serogroups IIa and IVb (Supplementary Table 3). Serogroup IIb was overrepresented in dairy products and serogroup IIc in both fresh and processed meat. Among the fish/shellfish products serogroup IVb, was associated with trout and serogroup IIa with salmon and herring (Supplementary Table 2).

The distribution of the serogroups among the human *Lm* sequences varied over the years, with the major fluctuations observed for serogroups IVb and IIa (Supplementary Figure 3). Thus, serogroup IVb accounted for 55.6% of the sequences in 2010, it dropped to 29.2% in 2016, and it raised again to 51.1% in the past years. Serogroup IIa accounted for 33.3% in 2010, it dropped to 17.6% in 2011, and it raised again to 38.5% in the past years (Supplementary Figure 3).

#### 3.4.3. Clonal complexes

**Figure 2:**
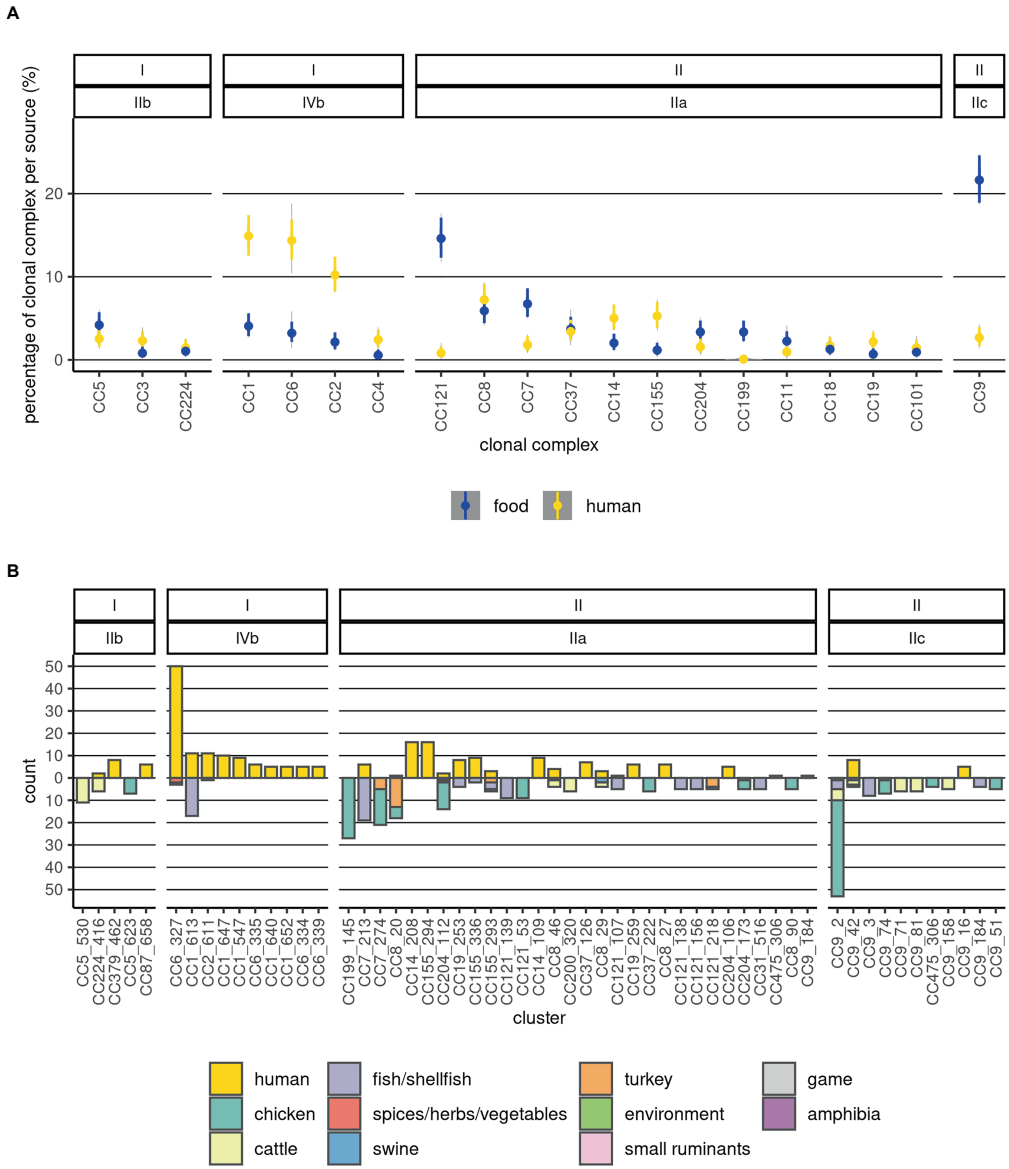
Distribution of sources and food product categories at CC and cluster levels. **(A)** The 20 most abundant CCs are depicted. Prevalences of CCs in the two sources are visualized as violin plots summarizing 10,000 draws of a Dirichlet distribution with probabilities equal to the observed ones; this was done in order to estimate the uncertainty associated with different and low sample sizes across the categories. **(B)** Distribution of the clusters by source and category of food products from which they were isolated. Counts of human *Lm* sequences are shown above the null of y-axis and those of food *Lm* sequences below it. The labels on top indicate the lineage and serogroup, and the x-axis labels indicate the CC and cluster. The sample type and food categories are depicted as fractions of the bars with distinct colors.

The CC or ST was determined for 1,517 sequences (99.4%). Most of the CCs comprised one serogroup (Supplementary Table 1; Figure 1). However, several sequences in each of the CCs CC9, CC14, CC101, CC204, and CC475 presented a different serogroup from the majority of the sequences in the respective CCs (switches between serogroups IIa and IIc). Of the 59 CCs identified, six contained more than 50% of all sequences: CC1, CC2, CC3, CC4, CC5, and CC6, and 30 contained 95% of the sequences. The dominant (comprising more than 50% of the sequences) CCs among food *Lm* sequences were: CC7, CC8, CC9, and CC121, while among human *Lm* sequences CC1, CC2, CC6, and CC8 prevailed (Figure 2A). Our results indicate that the prevalence of several CCs among human *Lm* sequences is significantly higher than expected based on their distribution among the food *Lm* sequences; these are CC1, CC2, CC4, CC6, CC14, CC19, CC155, and CC379. Other CCs, on the contrary, have been found significantly less in humans and more often in food - CC7, CC9, CC31, CC121, CC199, and CC321 (Figure 2A; Supplementary Tables 4, 5).

Among the food *Lm* sequences, CC199 and CC204 were associated with chicken products (Figure 2; Supplementary Table 1). Sequences from cattle-derived food products were overrepresented in CC9, CC200, and CC224. Fish/shellfish sequences were positively associated with CC1, CC7, CC19, CC31, CC121, and CC193. A large fraction of the turkey *Lm* sequences was found in CC8 (Supplementary Table 3). CC5 and CC9 were overrepresented in fresh meat. Additionally, CC200 was associated with processed meat. Associations of CCs with fish/shellfish products were: CC1 with trout, CC7, CC19, and CC31 with salmon, CC121 and CC193 with herring, and CC321 with shrimp (Supplementary Table 2).

#### 3.4.4. Clusters

A total of 187 clusters were detected, comprising 917 sequences (396 human, and 521 food; Supplementary Table 1) and 360 (47.6%) of the human *Lm* sequences were singletons. Of the 187 clusters, 110 contained more than 50% of all sequences. There were 33 (17.6%) clusters that comprised at least one human and one food *Lm* sequence (*n* = 143 human *Lm* sequences; 18.9% of the human *Lm* sequences). Clusters with only human *Lm* sequences accounted for 35.8% (67/187) of all clusters, and 33.5% (253/756) of the human *Lm* sequences (Supplementary Figure 4). There were 83 clusters that comprised at least two human *Lm* sequences (*n* = 379; 50.1% of the human *Lm* sequences). Clusters with only food *Lm* sequences accounted for 46.5% of all clusters (87/187), and 42% of all clustering sequences (385/917).

The size of the clusters varied largely, with 81 clusters containing only two sequences each, and the largest cluster containing 53 sequences; this latter cluster was part of CC9 (serogroup IIc), and it was present exclusively in food *Lm* sequences. The top 10 ranked largest clusters contained each a minimum of 16 sequences, and three of them consisted only of food *Lm* sequences while the remaining had both human and food *Lm* sequences. The largest clusters among the food *Lm* sequences were found in CC1, CC5, CC7, CC8, CC9, CC121, CC199, and CC204, while the largest ones among the human *Lm* sequences were part of CCs CC1, CC2, CC6, CC9, CC14, CC19, CC155, and CC379 (Figure 2B).

A number of three clusters, belonging to CC6, CC14, and CC155, were overrepresented among the human *Lm* sequences compared to the food ones, with some never being sampled in food products (Figure 2B). In contrast, other four clusters were significantly overrepresented among the food *Lm* sequences - these belong to CC7, CC8, CC9, and CC199 (Supplementary Tables 4,5). Out of the 33 clusters that contained both human (n = 143) and food (n = 136) sequences, the highest number of clusters could be linked to fish/shellfish sequences. However, the highest number of human *Lm* sequences did not link to fish/shellfish, but to cattle products (60 fish-linked vs. 72 cattle-linked; Supplementary Table 1). The composition of the cgMLST clusters was, however, not always homogeneous in terms of categories of food products. Thus, 10 of the 33 clusters contained sequences from more than one food category. In the attempt to define *Lm* populations by the main food categories we observed that the genetic differentiation indices within the various *Lm* populations were generally much larger than the ones between the populations (Supplementary Figure 5). This indicates that there is no clear differentiation between the *Lm* in the various food categories.

**Figure 3:**
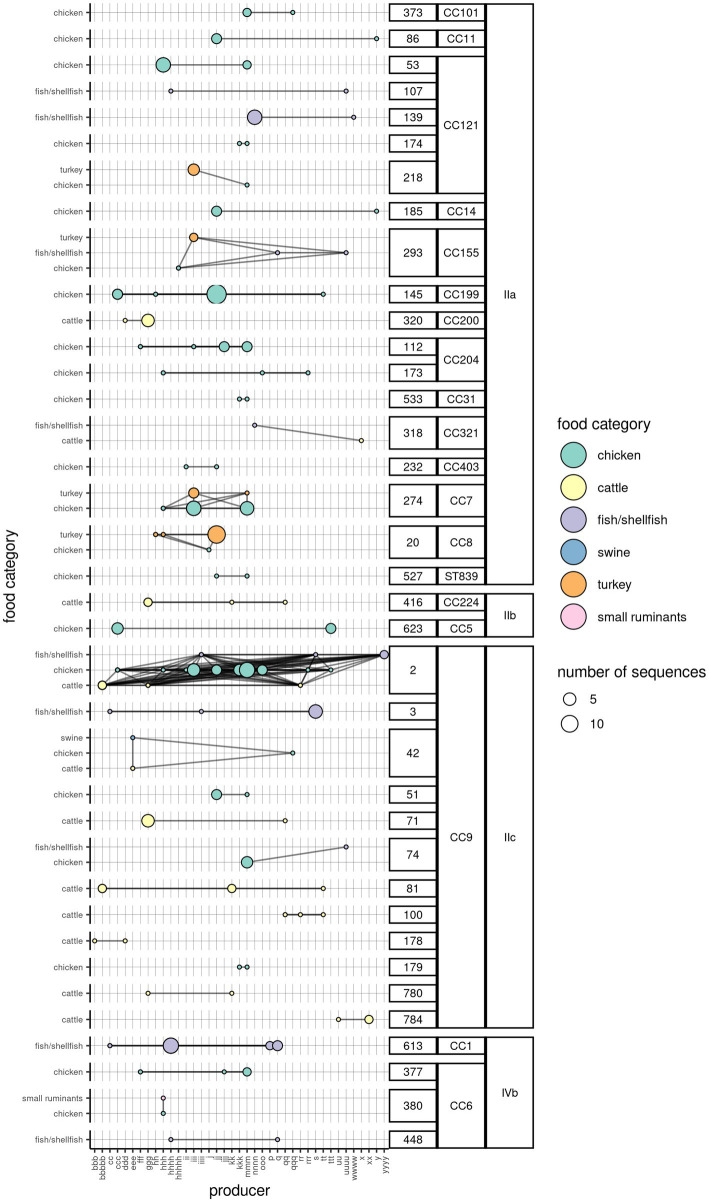
Network of *Lm* clusters among the food product categories and the corresponding producers. One circle indicates the presence of a particular cluster in a food category (*y*-axis), originating from a producer (*x*-axis). The color of the circle is indicative of the food category, while its size indicates the number of WG sequences. The horizontal lines connecting any two circles indicate that the cluster is present in the same food product category across multiple producers. The vertical lines connecting any two circles indicate that the cluster is present in more than one food product categories from the same producer. The oblique lines indicate the presence of the same cluster in different food categories from different producers. The clusters are aggregated by their membership to the CC and serogroup (labels on the right).

For a subset of sequences retrieved from food products (504; 65.5%) the producer was also known. The number of *Lm* sequences from each FBO was on median 4 (range = 1–78, interquartile range = 2–10). The analysis of *Lm* clusters according to the FBO indicated that 36 clusters (19.3%) were present in food products coming from more than one FBO, with one cluster containing sequences coming from up to 16 FBOs. The majority of the clusters (n = 28) were found in food products belonging to one category. However, nine clusters were found in up to three food product categories. Furthermore, three clusters were found each in two product categories from four different FBOs (Figure 3).

### 3.5. Persistence of clusters

**Figure 4:**
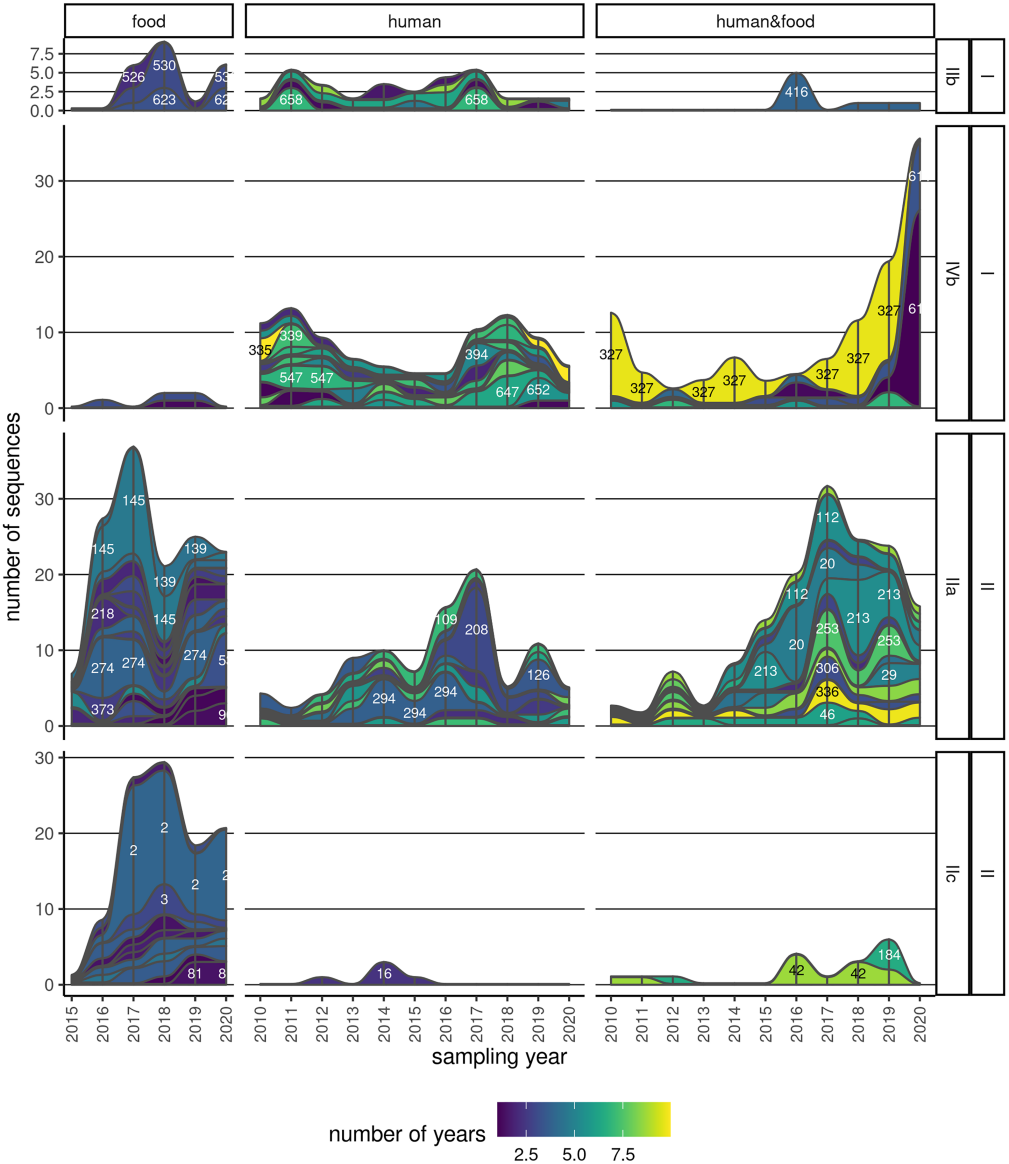
Persistence of clusters across multiple years. One continuous strip represents one cluster, with the corresponding name/number indicated on the strip, and its membership to serogroup and lineage indicated on the labels on the right. The color is indicative of the time span that the cluster has been found in either human, food, or both sample types. The thickness of the strip is proportional to the number of sequences in the cluster in a particular year.

A considerable number of clusters (111/187; 59.4%) were observed over multiple years (Figure 4; Supplementary Figure 6). About a third (42/111, 37.8%) of these clusters contained only human *Lm* sequences, and another 26.1% (29/111) contained *Lm* sequences of both human and food provenience (Supplementary Figure 6). The majority of the persistent clusters belonged to CC9 (16/111; 14.4%), followed by CC6 (14/111; 12.6%). Clonal complexes CC1, CC8, and CC121 also contained above 10 persistent clusters. One particular cluster (CC6_335) was found in an interval spanning over 10 years, and it comprised six sequences. Other four clusters in serogroup IIa (CC8, CC121, and CC155), two clusters in serogroup IVb (CC6), and one cluster in serogroup IIb (CC5) were found across 8 years (Supplementary Table 6). Some of the most abundant and persistent clusters in food products (CC9_3, CC199_154, and CC7_293) did not include human *Lm* sequences (Figure 4; Supplementary Figure 4).

### 3.6. Sources of outbreaks and sporadic cases

From 2015 to 2020 six outbreaks have been identified by using the match of human and food *Lm* WGS sequences. Three of these (CC1_613, n_human = 11; CC2_611, n_human = 11; CC7_213, n_human = 6) have been linked to fish, and other three (CC6_327, n_human = 50; CC8_23, n_human = 4; CC8_27, n_human = 6) with cold cuts of beef. Cluster CC6_327 contained, in addition to the isolate from beef products, also two isolates from spices/herbs/vegetables from an earlier year. The median distances withing the clusters corresponding to the outbreaks ranged from 1–7, while the maximum distances ranged 2–18.

**Figure 5:**
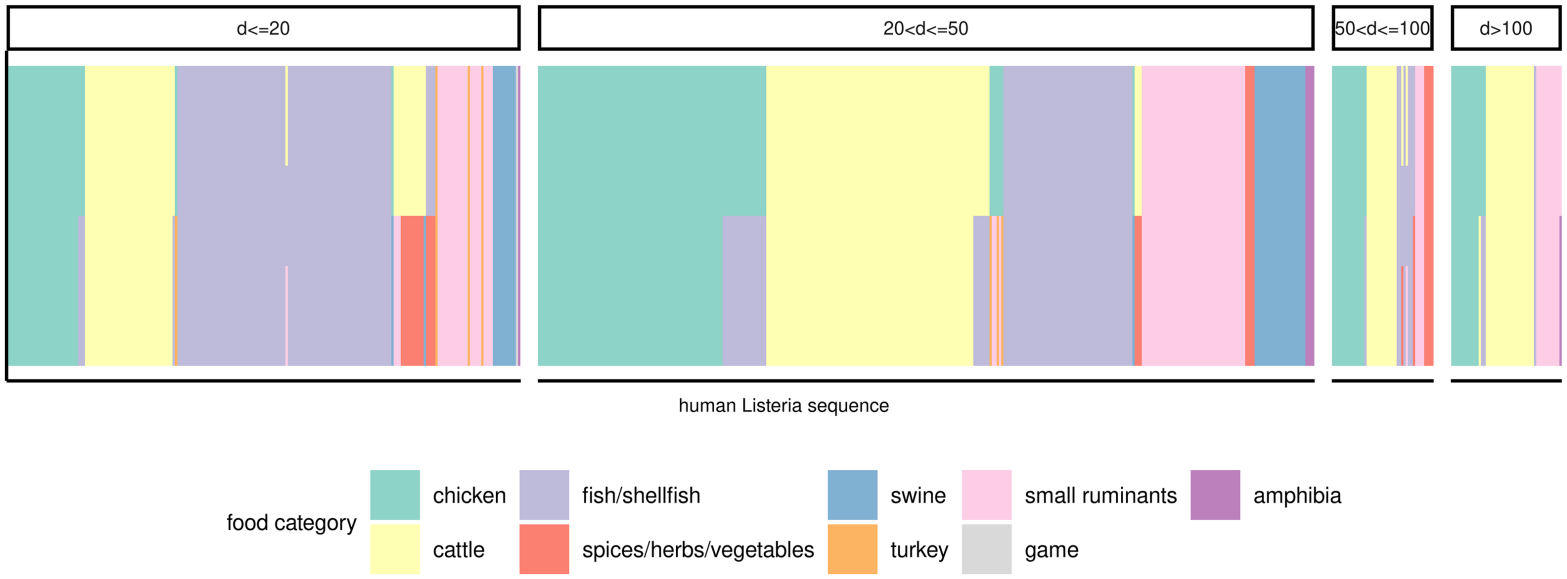
Potential food sources for the sporadic human *Lm* isolates. One vertical bar corresponds to a human *Lm* sequence (*x*-axis). The color of the bars is indicative of the food category the respective human *Lm* sequence was most similar with (i.e., smallest distance). Bars containing multiple colors indicate that two or three food categories were equally distanced from the human *Lm* sequence. The various panels indicate the range of distances between the respective human *Lm* sequences and the matching food categories.

There were 379 (50.1%) human *Lm* sequences that clustered (minimum cluster size of two) in 83 clusters. Another 17 human *Lm* sequences did not form clusters with any other human *Lm* sequences, but clustered with at least one food *Lm* sequence. The remaining 360 human *Lm* sequences did not cluster. Officially, the only sequences that were part of an outbreak were those implicated in the outbreaks described above and we have, therefore, extended our sporadic sequences subset to include all but the outbreak sequences (*n* = 652). In an attempt to infer a potential source for these isolates, we have measured the distances to the nearest food *Lm* sequences in the dataset. Many human *Lm* sequences (*n* = 69) were equidistant from two or three food categories. Of the remaining 583 sequences of sporadic cases, a large proportion were closest to *Lm* sequences from cattle products (24.9%), fish/shellfish products (23.6%), and chicken products (21%), followed by products from small ruminants (12.4%). Only 30.4% of the human *Lm* sequences of sporadic cases had a food *Lm* sequence nearest neighbor within 20 alleles, while a majority of them (46.2%) had a nearest neighbor food *Lm* sequence between 20–50 alleles distance, and other 12.9% human *Lm* sequences were at least 50 alleles removed from the nearest food *Lm* sequence (Figure 5; Supplementary Table 7). Among the food categories with large distances to the human *Lm* sequences, the best represented were also the most abundant food categories in the dataset.

### 3.7. Associations of genotype with epidemiological variables

The Spearman correlation coefficients indicated that use of immunosuppressives was positively correlated with use of antacids (0.33) and the presence of comorbidities (0.23). Likewise, pregnancy was positively correlated with the host sex (0.31), and negatively with the host age (−0.34), use of antacids (−0.22), and use of immunosuppressives (−0.20). Consequently, to avoid multiple collinearity in the models, the variables use of immunosuppressives and pregnancy were discarded.

The relative risk ratios (RR) coefficients of the multinomial models indicated that the use of antacids could be a risk factor for infection with lineage II (RR = 1.44, *p* = 0.04). Some CCs were overrepresented in the older age groups: CC6 (in age group [65,80), RR = 2.34, *p* = 0.013), CC14 (in both age group [65,80), RR = 6.97, *p* < 0.01, and [80,100), RR = 3.98, *p* = 0.029), and CC19 (in age group [80,100), RR = 8.89, *p* = 0.048). Similarly, ST6 and ST399 were slightly overrepresented in the age group [65,80). ST14 was associated with the patient’s gender, with a RR of 18.06 for women; likewise, ST14 was overrepresented in women (RR = 18.06, *p* < 0.01; Supplementary Table 8). From the three CCs overrepresented in elderly, CC19 was positively associated with environmental samples, while the other two CCs did not show any significant associations with a particular food category. CC9 and CC121, which have been previously found associated with heavily immunocompromised patients, were not significantly associated with neither use of immunosuppressives nor old age.

## 4. Discussion

Detection and source tracing of listeriosis outbreaks is hampered by the long incubation period, the relatively small fraction of the population at risk (elderly, immunocompromised, nenates, and pregnant women), and the ensuing low incidence rate. Since, 2017 human and food *Lm* WGS and metadata have been used for real-time integrated surveillance in the Netherlands. On several occasions this has led to matches of clusters of human *Lm* sequences to food *Lm* sequences, facilitating interventions on the sources of infection. With this study we have performed a retrospective analysis on the combined dataset of human and food *Lm* sequences, in order to reveal long-term temporal patterns in clustering and potential associations of *Lm* genotypes with food products and humans.

### 4.1. Epidemiological trends

Our data showed a mild increase in the incidence of listeriosis in the past 11 years. This might partially be explained by an increasing population of elderly (both relative and absolute), associated with the inherent age-related health issues as well as with increased usage of immunosupressives for this age category (Wallace et al., 2021). However, an improved surveillance system could, at least partly, account for an apparent increase in incidence. The age distribution of the clinical cases indicated that the population groups at risk are elderly people (65+) and pregnant women. More men than women were infected with *Lm*, particularly in the age group of 65+, as described also in previous studies (Herrador et al., 2019; Lüth et al., 2020). The reason for this remains speculative but might be related to the differential IL-10 production between the genders (Pasche et al., 2005), and/or behavioral differences between the sexes regarding food consumption. The distribution of the symptoms is comparable to general knowledge on listeriosis symptomatology (Koch and Stark, 2006; Bortolussi, 2008; Koopmans et al., 2013).

### 4.2. Hypervirulent CCs are overrepresented among clinical isolates but no epidemiological risk factors could be identified

The combined retrospective analysis of *Lm* sequences from food and humans offered the opportunity to assess comparatively the prevalence of the various *Lm* genotypes in the two sources, with subsequent generation of hypotheses regarding the most likely sources for human infection. Our analysis confirmed previous findings (Chenal-Francisque et al., 2011; Maury et al., 2016, 2019) regarding the skewed distribution of CCs among clinical and food isolates, with CC1, CC2, CC4, CC6, CC14, and CC379 more commonly encountered in clinical cases and CC7, CC31, CC121, CC199, CC204, and CC321 being overrepresented in food. This observation has previously led to hypothesize the existence of hyper- (CC1, CC2, CC4, and CC6) and hypo-virulent (CC9, CC121) *Lm* clones (Maury et al., 2016, 2019). The presumably hypovirulent *Lm* could come to infect only patients that are heavily immunocompromised, while presumably hypervirulent bacteria can also infect patients with a milder level of immunosuppression and/or comorbidities (Maury et al., 2016, 2019). While we found several CCs (CC6, CC14, CC19) and STs (ST6 and ST14) positively associated with older age groups, we did not find any associations of *Lm* genotypes and specific comorbidities. This highlights that it remains difficult to epidemiologically categorize the virulence of the various *Lm* types, possibly due to limited diversity in clinical forms among reported listeriosis cases, as mild cases usually go unreported, but also due to limited monitoring of the symptoms.

While cgMLST is a valuable tool for surveillance, the complexity of bacterial genomes extends beyond that, and different genomic elements might regulate the ability of the bacteria to grow in various environments. Similarly, the impact of the matrix on *Lm* detection may affect the observed distribution of the bacterial types across sources. In this study, in comparing the bacterial types across multiple sources of isolation, one of the main assumptions is that the ability of culturing and detecting the respective types is independent of the matrix/substrate the bacteria are embedded in. Our results indicate that the current paradigm that *Lm* types are equally pathogenic, should be reconsidered. However, further experiments on the impact of the matrix on *Lm* detection as well as comparative genomics analyses would be required in order to test the differential virulence hypothesis.

### 4.3. Integrated cross-domain WGS-based surveillance improved detection of clusters and source finding

Approximately a third of the clusters containing human *Lm* sequences also contained food *Lm* sequences. This shows the increased potential for source identification when using an integrated clinical-food WGS-based surveillance system. Starting with 2017, this integrated approach has facilitated source tracing for four larger clusters of human cases. For three of these clusters the source was in the fish/shellfish food category, and for three others the source was identified as cold cuts of beef. The direct comparison of the distances within clusters showed that, although the median distance can range between 0 (for the smallest clusters) and 11, the maximum distance can be in effect as large as 18. This is a result of, next to the cluster size, the linkage method chosen in the clustering algorithm; in this case the single-linkage will allow clustering of sequences with pairwise distances, on occasion, much higher than the set threshold of seven. Although this is not an impediment in using single-linkage, the results thereof must be interpreted with caution and often accompanied by extra analyses for refining and redefining the clusters, as seen here.

The integration of the WGS data from two different sectors also came with transition to a commonly accepted typing method - cgMLST. Until 2016 the molecular typing was based on PFGE, which is generally known to have low reproducibility. We have shown here that although the number of clusters identified with PFGE was comparable to that generated by cgMLST, the concordance of the clusters was very poor. Our finding is in line with other recent studies that have found the partitionings from the two methods to be poorly correlated (Henri et al., 2017; Moura et al., 2017).

### 4.4. General attribution of *Lm* to food categories is challenged by the ubiquitous nature of the pathogen: The importance of attribution at producer level

We found a few associations of various *Lm* types (CC, ST and cluster level) with certain food categories. For example, CC1 was positively associated with both human isolates and fish/shellfish products, suggesting this food category as potential source. This is in contrast with other studies that have found this CC to be associated with cattle and dairy products (Maury et al., 2019). Furthermore, the specific associations with food products are reliant on a balanced sampling of the various food categories. An oversampling of certain foods due to prior belief or experience of their contamination with *Lm* (resulting in risk-based sampling) would potentially bias the distribution of the *Lm* types.

Other major CCs, such as CC2, CC4, and CC6, did not show any associations with particular food categories. In fact, most of the typing levels of *Lm* were heterogeneous in their composition by food category. Thus, the *Lm* population had overall a structure that did not correlate with the food categories defined according to the animal species they come from (as indicated by the genetic differentiation indices). This is not entirely surprising, as *Lm* is an environmental bacterium, which, unlike other food-borne bacteria, is not primarily maintained in vertebrate reservoir hosts (Vivant et al., 2013). The direct implication hereof for public health is that a particular *Lm* strain might be able to contaminate equally well food products coming from very different categories/animal species. The existence of large food-processing plants that process different food categories (from various animal species) would facilitate the spread of similar *Lm* types over different food products. We have shown that some clusters are present in food products from multiple producers, which is compatible with the hypothesis of contaminated raw materials. Furthermore, we have shown that the same cluster can be present even in various categories of food products from the same producer, which is compatible with the hypothesis of cross-contamination in the production process. These findings indicate that source attribution of *Lm* based on presence of various types in food product categories, defined based on the animal species they come from, is likely to be of limited use, and that additional information, such as food consumption statistics or production facility, is required to disambiguate the source of the circulating *Lm*.

Based on Hamming distances, we have shown that approximately 50% of all human cases might come in equal proportions from cattle, fish/shellfish, and chicken, and about 10% from small ruminants. This is a very simple approach to quantify the source of the human *Lm* sequences, and it has two main drawbacks: (a) there might be multiple food *Lm* sequences at comparable, yet not equal distances from the human *Lm* sequences, but only the smallest is accounted for; (b) only about a third of the human *Lm* sequences were within 20 alleles distance from a food *Lm* sequence, about 40% found a match in between 20 and 50 alleles distance, and yet another 10% were at >50 alleles distance from a food *Lm* sequence. Additionally, about 11% of all human *Lm* sequences were equidistant from sequences belonging to different food categories making it thus impossible to establish a likely source and illustrating the direct impact of the high genetic heterogeneity of *Lm* within the various food categories.

### 4.5. *Lm* is characterized by a high degree of temporally persistent clustering

We identified a surprisingly high degree of temporal persistence of clusters with almost 60% of the identified clusters persisting over multiple years (up to 10 years). Under the assumption that two very similar sequences are likely to be descendants of a recent common ancestor, and that they have remained relatively unchanged over time, this may indicate the existence of persistent sources. The identification and eradication of the persistent sources that have been linked to human cases of listeriosis might provide a substantial reduction in disease burden. It is known that *Lm* can persist for prolonged periods of time in parts of production plants (Cruz and Fletcher, 2011) but also that contaminated raw materials can end up at different producers (Lüth et al., 2020). Further comparative genomics and phylogenetics studies (providing a higher resolution than cgMLST) are required to be able to address the question as to whether isolates in these persistent clusters are truly descendants of a recent common ancestor or whether they are the result of multiple introduction events and subsequent clonal evolution. Additionally, extensive and systematic sampling of *Lm* from the environment, combined with probabilistics for the chance of finding two or more similar sequences given random sampling from natural populations, would be required.

## 5. Conclusion

Our results highlight the utility of combined human-food WGS analyses for *Lm* and the added value of a shared database of bacterial cgMLST profiles for public health surveillance. This will contribute to timely detection of links between human and food bacterial sequences and a faster detection of the source in outbreaks, or even to prevention of these outbreaks. The main findings of this study include the observation of a very weak attribution of *Lm* types to animal species and a much better one to the producer level. In addition, we identified a high degree of temporal persistence of clusters. Together this would indicate that identifying persistent contamination and producers that process a wide variety of raw produce, and applying intervention measures hereupon could significantly contribute to lowering the *Lm* disease burden. On a scientific level, further efforts to incorporate also environmental samples into surveillance databases will contribute to a better understanding of bacterial evolution and persistence. Additional knowledge on the conditions during the food production process and their influence on the evolution of *Lm* (i.e., substitution rate) would also allow for the development of better predictive models of identity by descent.

## Data availability statement

The datasets presented in this study can be found in online repositories. The names of the repository/repositories and accession number(s) can be found in the article/supplementary material.

## Author contributions

AvH, TvdB, MvdB, SK, LMG, IB, ThB, BW, and MvdV contributed with isolate sequences and associated metadata. IF analyzed the epidemiological data. CC performed genomics and statistical analyses. CC and EF wrote the manuscript. All authors reviewed and approved the manuscript.

## Funding

This study was financed by the National Food and Consumer Products Authority and the Ministry of Health, Welfare and Sports in the Netherlands.

## Conflict of interest

The authors declare that the research was conducted in the absence of any commercial or financial relationships that could be construed as a potential conflict of interest.

## Publisher’s note

All claims expressed in this article are solely those of the authors and do not necessarily represent those of their affiliated organizations, or those of the publisher, the editors and the reviewers. Any product that may be evaluated in this article, or claim that may be made by its manufacturer, is not guaranteed or endorsed by the publisher.
