# Supplementary material for: New insights into the epidemiology of Listeria monocytogenes – A cross-sectoral retrospective genomic analysis in the Netherlands (2010–2020)
Source: Front Microbiol. 2023 Apr 6;14:1147137. doi: 10.3389/fmicb.2023.1147137 (PMC10118018; doi:10.3389/fmicb.2023.1147137)
Supplement: Supplementary file 1 [file Data_Sheet_1.docx]

## Supplementary material

### Supplementary tables

**Supplementary Table 1**: Sources and genotypes of the *Lm* sequences; cluster_s7=clusters have been delineated using single-linkage hierarchical clustering with a threshold of seven

| source | food_category | lineage | serotype | CC | ST | cgMLST_cluster | count |
| --- | --- | --- | --- | --- | --- | --- | --- |
| food | chicken | I | IIb | CC288 | ST288 | 747 | 1 |
| food | chicken | I | IIb | CC5 | ST5 | 524 | 1 |
| food | chicken | I | IIb | CC5 | ST5 | 526 | 4 |
| food | chicken | I | IIb | CC5 | ST5 | 535 | 1 |
| food | chicken | I | IIb | CC5 | ST5 | 538 | 1 |
| food | chicken | I | IIb | CC5 | ST5 | 623 | 7 |
| food | chicken | I | IIb | CC5 | ST5 | 686 | 1 |
| food | chicken | I | IIb | CC5 | ST5 | 735 | 1 |
| food | chicken | I | IIb | CC5 | ST5 | 739 | 1 |
| food | chicken | I | IIb | CC87 | ST87 | 682 | 1 |
| food | chicken | I | IIb | CC87 | ST87 | 740 | 2 |
| food | chicken | I | IVb | CC1 | ST1 | 622 | 1 |
| food | chicken | I | IVb | CC1 | ST1 | 625 | 3 |
| food | chicken | I | IVb | CC1 | ST1 | 630 | 1 |
| food | chicken | I | IVb | CC1 | ST1 | 685 | 1 |
| food | chicken | I | IVb | CC1 | ST1 | 737 | 1 |
| food | chicken | I | IVb | CC1 | ST1 | 748 | 1 |
| food | chicken | I | IVb | CC2 | ST2 | 633 | 1 |
| food | chicken | I | IVb | CC6 | ST6 | 350 | 1 |
| food | chicken | I | IVb | CC6 | ST6 | 377 | 4 |
| food | chicken | I | IVb | CC6 | ST6 | 379 | 1 |
| food | chicken | I | IVb | CC6 | ST6 | 380 | 1 |
| food | chicken | I | IVb | CC6 | ST6 | 381 | 2 |
| food | chicken | I | IVb | CC6 | ST6 | 447 | 1 |
| food | chicken | I | IVb | ST32 | ST32 | 680 | 1 |
| food | chicken | II | IIa | CC101 | ST431 | 373 | 4 |
| food | chicken | II | IIa | CC11 | ST11 | 315 | 1 |
| food | chicken | II | IIa | CC11 | ST451 | 62 | 2 |
| food | chicken | II | IIa | CC11 | ST451 | 64 | 1 |
| food | chicken | II | IIa | CC11 | ST451 | 86 | 4 |
| food | chicken | II | IIa | CC11 | ST451 | 91 | 1 |
| food | chicken | II | IIa | CC11 | ST451 | 246 | 1 |
| food | chicken | II | IIa | CC121 | ST121 | 48 | 3 |
| food | chicken | II | IIa | CC121 | ST121 | 53 | 9 |
| food | chicken | II | IIa | CC121 | ST121 | 54 | 1 |
| food | chicken | II | IIa | CC121 | ST121 | 59 | 1 |
| food | chicken | II | IIa | CC121 | ST121 | 65 | 1 |
| food | chicken | II | IIa | CC121 | ST121 | 85 | 2 |
| food | chicken | II | IIa | CC121 | ST121 | 96 | 2 |
| food | chicken | II | IIa | CC121 | ST121 | 147 | 4 |
| food | chicken | II | IIa | CC121 | ST121 | 149 | 1 |
| food | chicken | II | IIa | CC121 | ST121 | 159 | 3 |
| food | chicken | II | IIa | CC121 | ST121 | 162 | 1 |
| food | chicken | II | IIa | CC121 | ST121 | 170 | 1 |
| food | chicken | II | IIa | CC121 | ST121 | 174 | 2 |
| food | chicken | II | IIa | CC121 | ST121 | 177 | 1 |
| food | chicken | II | IIa | CC121 | ST121 | 180 | 1 |
| food | chicken | II | IIa | CC121 | ST121 | 186 | 1 |
| food | chicken | II | IIa | CC121 | ST121 | 191 | 1 |
| food | chicken | II | IIa | CC121 | ST121 | 218 | 1 |
| food | chicken | II | IIa | CC121 | ST121 | 225 | 1 |
| food | chicken | II | IIa | CC121 | ST121 | 231 | 1 |
| food | chicken | II | IIa | CC121 | ST121 | 242 | 1 |
| food | chicken | II | IIa | CC121 | ST121 | 541 | 1 |
| food | chicken | II | IIa | CC121 | ST121 | 542 | 2 |
| food | chicken | II | IIa | CC14 | ST14 | 230 | 1 |
| food | chicken | II | IIa | CC14 | ST399 | 185 | 4 |
| food | chicken | II | IIa | CC14 | ST91 | 276 | 1 |
| food | chicken | II | IIa | CC14 | ST91 | 311 | 1 |
| food | chicken | II | IIa | CC155 | ST155 | 293 | 1 |
| food | chicken | II | IIa | CC155 | ST155 | 319 | 1 |
| food | chicken | II | IIa | CC18 | ST18 | 309 | 2 |
| food | chicken | II | IIa | CC199 | ST230 | 145 | 27 |
| food | chicken | II | IIa | CC200 | ST200 | 215 | 2 |
| food | chicken | II | IIa | CC204 | ST204 | 80 | 1 |
| food | chicken | II | IIa | CC204 | ST204 | 112 | 12 |
| food | chicken | II | IIa | CC204 | ST204 | 163 | 1 |
| food | chicken | II | IIa | CC204 | ST204 | 173 | 4 |
| food | chicken | II | IIa | CC204 | ST204 | 221 | 1 |
| food | chicken | II | IIa | CC26 | ST26 | 281 | 1 |
| food | chicken | II | IIa | CC31 | ST31 | 525 | 1 |
| food | chicken | II | IIa | CC31 | ST31 | 533 | 2 |
| food | chicken | II | IIa | CC31 | ST31 | 537 | 1 |
| food | chicken | II | IIa | CC37 | ST37 | 55 | 1 |
| food | chicken | II | IIa | CC37 | ST37 | 165 | 1 |
| food | chicken | II | IIa | CC37 | ST37 | 168 | 2 |
| food | chicken | II | IIa | CC37 | ST37 | 222 | 6 |
| food | chicken | II | IIa | CC37 | ST37 | 233 | 1 |
| food | chicken | II | IIa | CC403 | ST403 | 167 | 1 |
| food | chicken | II | IIa | CC403 | ST403 | 228 | 2 |
| food | chicken | II | IIa | CC403 | ST403 | 232 | 2 |
| food | chicken | II | IIa | CC475 | ST504 | 290 | 1 |
| food | chicken | II | IIa | CC475 | ST504 | 306 | 2 |
| food | chicken | II | IIa | CC475 | ST504 | 372 | 1 |
| food | chicken | II | IIa | CC475 | ST504 | 375 | 1 |
| food | chicken | II | IIa | CC475 | ST504 | 376 | 1 |
| food | chicken | II | IIa | CC475 | ST504 | 383 | 1 |
| food | chicken | II | IIa | CC7 | ST7 | 229 | 2 |
| food | chicken | II | IIa | CC7 | ST7 | 236 | 2 |
| food | chicken | II | IIa | CC7 | ST7 | 238 | 1 |
| food | chicken | II | IIa | CC7 | ST7 | 245 | 1 |
| food | chicken | II | IIa | CC7 | ST7 | 274 | 16 |
| food | chicken | II | IIa | CC7 | ST7 | 308 | 2 |
| food | chicken | II | IIa | CC7 | ST7 | 313 | 1 |
| food | chicken | II | IIa | CC8 | ST30 | 152 | 1 |
| food | chicken | II | IIa | CC8 | ST8 | 14 | 1 |
| food | chicken | II | IIa | CC8 | ST8 | 20 | 5 |
| food | chicken | II | IIa | CC8 | ST8 | 90 | 5 |
| food | chicken | II | IIa | CC8 | ST8 | 776 | 1 |
| food | chicken | II | IIa | CC8 | ST8 | 782 | 1 |
| food | chicken | II | IIa | CC8 | ST8 | 783 | 1 |
| food | chicken | II | IIa | CC8 | ST8 | 792 | 1 |
| food | chicken | II | IIa | ST839 | ST839 | 527 | 2 |
| food | chicken | II | IIc | CC101 | ST431 | 436 | 1 |
| food | chicken | II | IIc | CC475 | ST504 | 306 | 2 |
| food | chicken | II | IIc | CC9 | ST1031 | 78 | 1 |
| food | chicken | II | IIc | CC9 | ST580 | 781 | 1 |
| food | chicken | II | IIc | CC9 | ST9 | 2 | 42 |
| food | chicken | II | IIc | CC9 | ST9 | 41 | 1 |
| food | chicken | II | IIc | CC9 | ST9 | 42 | 1 |
| food | chicken | II | IIc | CC9 | ST9 | 45 | 1 |
| food | chicken | II | IIc | CC9 | ST9 | 51 | 5 |
| food | chicken | II | IIc | CC9 | ST9 | 63 | 1 |
| food | chicken | II | IIc | CC9 | ST9 | 74 | 6 |
| food | chicken | II | IIc | CC9 | ST9 | 79 | 1 |
| food | chicken | II | IIc | CC9 | ST9 | 148 | 2 |
| food | chicken | II | IIc | CC9 | ST9 | 166 | 1 |
| food | chicken | II | IIc | CC9 | ST9 | 179 | 2 |
| food | chicken | II | IIc | CC9 | ST9 | 189 | 1 |
| food | chicken | II | IIc | CC9 | ST9 | 240 | 1 |
| food | chicken | II | IIc | CC9 | ST9 | 243 | 1 |
| food | chicken | II | IIc | CC9 | ST9 | 787 | 1 |
| food | chicken | II | IIc | CC9 | (Missing) | 2 | 1 |
| food | cattle | I | IIb | CC224 | ST224 | 416 | 6 |
| food | cattle | I | IIb | CC224 | ST224 | 534 | 1 |
| food | cattle | I | IIb | CC3 | ST3 | 520 | 1 |
| food | cattle | I | IIb | CC3 | ST3 | 531 | 1 |
| food | cattle | I | IIb | CC3 | ST3 | 618 | 1 |
| food | cattle | I | IIb | CC379 | ST379 | 449 | 1 |
| food | cattle | I | IIb | CC426 | ST426 | 446 | 1 |
| food | cattle | I | IIb | CC489 | ST489 | 438 | 1 |
| food | cattle | I | IIb | CC5 | ST5 | 450 | 1 |
| food | cattle | I | IIb | CC5 | ST5 | 530 | 11 |
| food | cattle | I | IIb | CC5 | ST5 | 734 | 1 |
| food | cattle | I | IIb | CC59 | ST59 | 437 | 1 |
| food | cattle | I | IIb | CC59 | ST59 | 617 | 1 |
| food | cattle | I | IIb | CC59 | ST59 | 626 | 1 |
| food | cattle | I | IIb | CC77 | ST77 | 439 | 1 |
| food | cattle | I | IIb | (Missing) | (Missing) | 631 | 1 |
| food | cattle | I | IVb | CC1 | ST1 | 627 | 1 |
| food | cattle | I | IVb | CC1 | ST1 | 678 | 1 |
| food | cattle | I | IVb | CC1 | ST1 | 679 | 1 |
| food | cattle | I | IVb | CC1 | ST1 | 683 | 1 |
| food | cattle | I | IVb | CC1 | ST1 | 708 | 1 |
| food | cattle | I | IVb | CC1 | ST1 | 716 | 1 |
| food | cattle | I | IVb | CC2 | ST2 | 521 | 1 |
| food | cattle | I | IVb | CC2 | ST2 | 522 | 1 |
| food | cattle | I | IVb | CC2 | ST2 | 532 | 1 |
| food | cattle | I | IVb | CC2 | ST2 | 619 | 1 |
| food | cattle | I | IVb | CC2 | ST2 | 633 | 1 |
| food | cattle | I | IVb | CC2 | ST2 | 634 | 1 |
| food | cattle | I | IVb | CC2 | ST2 | 635 | 1 |
| food | cattle | I | IVb | CC2 | ST2 | 676 | 1 |
| food | cattle | I | IVb | CC218 | ST218 | 632 | 1 |
| food | cattle | I | IVb | CC4 | ST816 | 687 | 1 |
| food | cattle | I | IVb | CC54 | ST54 | 443 | 1 |
| food | cattle | I | IVb | CC6 | ST6 | 327 | 1 |
| food | cattle | I | IVb | CC6 | ST6 | 342 | 1 |
| food | cattle | I | IVb | CC6 | ST6 | 359 | 1 |
| food | cattle | I | IVb | CC6 | ST6 | 387 | 1 |
| food | cattle | I | IVb | CC6 | ST616 | 374 | 3 |
| food | cattle | I | IVb | (Missing) | (Missing) | 445 | 1 |
| food | cattle | II | (Missing) | CC200 | ST200 | 320 | 1 |
| food | cattle | II | IIa | CC101 | ST101 | 442 | 1 |
| food | cattle | II | IIa | CC101 | ST101 | 444 | 1 |
| food | cattle | II | IIa | CC11 | ST451 | 38 | 1 |
| food | cattle | II | IIa | CC11 | ST451 | 69 | 1 |
| food | cattle | II | IIa | CC11 | ST451 | 76 | 1 |
| food | cattle | II | IIa | CC11 | ST451 | 244 | 1 |
| food | cattle | II | IIa | CC11 | ST451 | 247 | 1 |
| food | cattle | II | IIa | CC11 | ST451 | 288 | 2 |
| food | cattle | II | IIa | CC121 | ST121 | 37 | 1 |
| food | cattle | II | IIa | CC121 | ST121 | 110 | 1 |
| food | cattle | II | IIa | CC121 | ST121 | 161 | 1 |
| food | cattle | II | IIa | CC121 | ST121 | 169 | 1 |
| food | cattle | II | IIa | CC121 | ST121 | 183 | 1 |
| food | cattle | II | IIa | CC121 | ST121 | 187 | 1 |
| food | cattle | II | IIa | CC121 | ST121 | 192 | 1 |
| food | cattle | II | IIa | CC121 | ST121 | 194 | 1 |
| food | cattle | II | IIa | CC121 | ST121 | 239 | 3 |
| food | cattle | II | IIa | CC121 | ST121 | 287 | 1 |
| food | cattle | II | IIa | CC121 | ST121 | 326 | 1 |
| food | cattle | II | IIa | CC121 | ST236 | 70 | 2 |
| food | cattle | II | IIa | CC124 | ST124 | 755 | 1 |
| food | cattle | II | IIa | CC14 | ST14 | 144 | 1 |
| food | cattle | II | IIa | CC14 | ST14 | 323 | 1 |
| food | cattle | II | IIa | CC14 | ST206 | 154 | 1 |
| food | cattle | II | IIa | CC14 | ST91 | 285 | 1 |
| food | cattle | II | IIa | CC14 | ST91 | 305 | 1 |
| food | cattle | II | IIa | CC14 | ST91 | 621 | 1 |
| food | cattle | II | IIa | CC18 | ST18 | 279 | 1 |
| food | cattle | II | IIa | CC18 | ST18 | 283 | 1 |
| food | cattle | II | IIa | CC18 | ST18 | 310 | 1 |
| food | cattle | II | IIa | CC19 | ST398 | 284 | 1 |
| food | cattle | II | IIa | CC20 | ST20 | 681 | 1 |
| food | cattle | II | IIa | CC200 | ST200 | 320 | 6 |
| food | cattle | II | IIa | CC204 | ST204 | 171 | 1 |
| food | cattle | II | IIa | CC21 | ST21 | 249 | 1 |
| food | cattle | II | IIa | CC29 | ST29 | 786 | 1 |
| food | cattle | II | IIa | CC31 | ST31 | 629 | 1 |
| food | cattle | II | IIa | CC321 | ST321 | 317 | 3 |
| food | cattle | II | IIa | CC321 | ST321 | 318 | 1 |
| food | cattle | II | IIa | CC37 | ST37 | 57 | 1 |
| food | cattle | II | IIa | CC37 | ST37 | 73 | 1 |
| food | cattle | II | IIa | CC37 | ST37 | 75 | 1 |
| food | cattle | II | IIa | CC37 | ST37 | 84 | 1 |
| food | cattle | II | IIa | CC37 | ST37 | 87 | 1 |
| food | cattle | II | IIa | CC37 | ST37 | 88 | 1 |
| food | cattle | II | IIa | CC37 | ST37 | 93 | 1 |
| food | cattle | II | IIa | CC37 | ST37 | 95 | 1 |
| food | cattle | II | IIa | CC37 | ST37 | 182 | 1 |
| food | cattle | II | IIa | CC412 | ST412 | 304 | 1 |
| food | cattle | II | IIa | CC7 | ST511 | 223 | 1 |
| food | cattle | II | IIa | CC7 | ST7 | 227 | 1 |
| food | cattle | II | IIa | CC7 | ST7 | 316 | 1 |
| food | cattle | II | IIa | CC8 | ST120 | 775 | 2 |
| food | cattle | II | IIa | CC8 | ST2117 | 29 | 2 |
| food | cattle | II | IIa | CC8 | ST8 | 46 | 3 |
| food | cattle | II | IIa | CC8 | ST8 | 795 | 1 |
| food | cattle | II | IIc | CC9 | ST580 | 71 | 6 |
| food | cattle | II | IIc | CC9 | ST580 | 752 | 1 |
| food | cattle | II | IIc | CC9 | ST9 | 1 | 3 |
| food | cattle | II | IIc | CC9 | ST9 | 2 | 5 |
| food | cattle | II | IIc | CC9 | ST9 | 36 | 1 |
| food | cattle | II | IIc | CC9 | ST9 | 40 | 4 |
| food | cattle | II | IIc | CC9 | ST9 | 42 | 2 |
| food | cattle | II | IIc | CC9 | ST9 | 44 | 1 |
| food | cattle | II | IIc | CC9 | ST9 | 45 | 3 |
| food | cattle | II | IIc | CC9 | ST9 | 50 | 2 |
| food | cattle | II | IIc | CC9 | ST9 | 56 | 1 |
| food | cattle | II | IIc | CC9 | ST9 | 72 | 3 |
| food | cattle | II | IIc | CC9 | ST9 | 81 | 6 |
| food | cattle | II | IIc | CC9 | ST9 | 89 | 1 |
| food | cattle | II | IIc | CC9 | ST9 | 92 | 1 |
| food | cattle | II | IIc | CC9 | ST9 | 94 | 1 |
| food | cattle | II | IIc | CC9 | ST9 | 97 | 2 |
| food | cattle | II | IIc | CC9 | ST9 | 100 | 3 |
| food | cattle | II | IIc | CC9 | ST9 | 150 | 2 |
| food | cattle | II | IIc | CC9 | ST9 | 153 | 1 |
| food | cattle | II | IIc | CC9 | ST9 | 155 | 1 |
| food | cattle | II | IIc | CC9 | ST9 | 157 | 1 |
| food | cattle | II | IIc | CC9 | ST9 | 158 | 5 |
| food | cattle | II | IIc | CC9 | ST9 | 176 | 1 |
| food | cattle | II | IIc | CC9 | ST9 | 178 | 3 |
| food | cattle | II | IIc | CC9 | ST9 | 188 | 1 |
| food | cattle | II | IIc | CC9 | ST9 | 241 | 1 |
| food | cattle | II | IIc | CC9 | ST9 | 275 | 1 |
| food | cattle | II | IIc | CC9 | ST9 | 282 | 1 |
| food | cattle | II | IIc | CC9 | ST9 | 289 | 1 |
| food | cattle | II | IIc | CC9 | ST9 | 314 | 1 |
| food | cattle | II | IIc | CC9 | ST9 | 382 | 3 |
| food | cattle | II | IIc | CC9 | ST9 | 756 | 1 |
| food | cattle | II | IIc | CC9 | ST9 | 780 | 2 |
| food | cattle | II | IIc | CC9 | ST9 | 784 | 3 |
| food | cattle | II | IIc | CC9 | ST9 | 785 | 1 |
| food | cattle | II | IIc | CC9 | ST9 | 788 | 1 |
| food | cattle | II | IIc | CC9 | ST9 | 790 | 1 |
| food | cattle | II | IIc | CC9 | ST9 | 791 | 1 |
| food | cattle | II | IIc | CC9 | ST9 | 796 | 1 |
| food | cattle | II | IIc | (Missing) | (Missing) | 248 | 1 |
| food | fish/shellfish | I | IIb | CC3 | ST3 | 684 | 1 |
| food | fish/shellfish | I | IIb | CC5 | ST5 | 620 | 1 |
| food | fish/shellfish | I | IVb | CC1 | ST1 | 613 | 17 |
| food | fish/shellfish | I | IVb | CC2 | ST2 | 425 | 1 |
| food | fish/shellfish | I | IVb | CC2 | ST2 | 611 | 1 |
| food | fish/shellfish | I | IVb | CC2 | ST2 | 688 | 1 |
| food | fish/shellfish | I | IVb | CC2 | ST2 | 731 | 1 |
| food | fish/shellfish | I | IVb | CC6 | ST6 | 355 | 1 |
| food | fish/shellfish | I | IVb | CC6 | ST6 | 384 | 1 |
| food | fish/shellfish | I | IVb | CC6 | ST6 | 385 | 1 |
| food | fish/shellfish | I | IVb | CC6 | ST6 | 448 | 2 |
| food | fish/shellfish | II | IIa | CC121 | ST121 | 60 | 1 |
| food | fish/shellfish | II | IIa | CC121 | ST121 | 66 | 2 |
| food | fish/shellfish | II | IIa | CC121 | ST121 | 67 | 3 |
| food | fish/shellfish | II | IIa | CC121 | ST121 | 68 | 2 |
| food | fish/shellfish | II | IIa | CC121 | ST121 | 77 | 1 |
| food | fish/shellfish | II | IIa | CC121 | ST121 | 107 | 5 |
| food | fish/shellfish | II | IIa | CC121 | ST121 | 138 | 5 |
| food | fish/shellfish | II | IIa | CC121 | ST121 | 139 | 9 |
| food | fish/shellfish | II | IIa | CC121 | ST121 | 140 | 2 |
| food | fish/shellfish | II | IIa | CC121 | ST121 | 156 | 5 |
| food | fish/shellfish | II | IIa | CC121 | ST121 | 164 | 1 |
| food | fish/shellfish | II | IIa | CC121 | ST121 | 172 | 2 |
| food | fish/shellfish | II | IIa | CC121 | ST121 | 175 | 1 |
| food | fish/shellfish | II | IIa | CC121 | ST121 | 226 | 1 |
| food | fish/shellfish | II | IIa | CC121 | ST121 | 237 | 1 |
| food | fish/shellfish | II | IIa | CC121 | ST121 | 324 | 3 |
| food | fish/shellfish | II | IIa | CC121 | ST121 | 733 | 1 |
| food | fish/shellfish | II | IIa | CC121 | ST236 | 83 | 4 |
| food | fish/shellfish | II | IIa | CC155 | ST155 | 293 | 3 |
| food | fish/shellfish | II | IIa | CC155 | ST155 | 336 | 2 |
| food | fish/shellfish | II | IIa | CC18 | ST18 | 270 | 1 |
| food | fish/shellfish | II | IIa | CC18 | ST18 | 272 | 1 |
| food | fish/shellfish | II | IIa | CC19 | ST173 | 253 | 4 |
| food | fish/shellfish | II | IIa | CC193 | ST193 | 615 | 3 |
| food | fish/shellfish | II | IIa | CC20 | ST20 | 181 | 1 |
| food | fish/shellfish | II | IIa | CC20 | ST20 | 235 | 1 |
| food | fish/shellfish | II | IIa | CC204 | ST204 | 190 | 2 |
| food | fish/shellfish | II | IIa | CC31 | ST31 | 516 | 5 |
| food | fish/shellfish | II | IIa | CC31 | ST31 | 528 | 2 |
| food | fish/shellfish | II | IIa | CC31 | ST31 | 529 | 1 |
| food | fish/shellfish | II | IIa | CC31 | ST31 | 536 | 1 |
| food | fish/shellfish | II | IIa | CC31 | ST31 | 539 | 1 |
| food | fish/shellfish | II | IIa | CC321 | ST321 | 307 | 3 |
| food | fish/shellfish | II | IIa | CC321 | ST321 | 318 | 1 |
| food | fish/shellfish | II | IIa | CC37 | ST37 | 82 | 2 |
| food | fish/shellfish | II | IIa | CC37 | ST37 | 98 | 1 |
| food | fish/shellfish | II | IIa | CC7 | ST7 | 213 | 19 |
| food | fish/shellfish | II | IIa | CC7 | ST7 | 264 | 1 |
| food | fish/shellfish | II | IIa | CC7 | ST7 | 286 | 1 |
| food | fish/shellfish | II | IIa | CC7 | ST7 | 312 | 1 |
| food | fish/shellfish | II | IIa | CC8 | ST8 | 135 | 1 |
| food | fish/shellfish | II | IIa | CC8 | ST8 | 762 | 1 |
| food | fish/shellfish | II | IIa | CC8 | ST8 | 777 | 1 |
| food | fish/shellfish | II | IIa | CC8 | ST8 | 789 | 1 |
| food | fish/shellfish | II | IIa | CC9 | ST9 | 234 | 1 |
| food | fish/shellfish | II | IIa | ST13 | ST13 | 736 | 1 |
| food | fish/shellfish | II | IIc | CC14 | ST14 | 101 | 1 |
| food | fish/shellfish | II | IIc | CC204 | ST204 | 190 | 1 |
| food | fish/shellfish | II | IIc | CC9 | ST9 | 2 | 4 |
| food | fish/shellfish | II | IIc | CC9 | ST9 | 3 | 8 |
| food | fish/shellfish | II | IIc | CC9 | ST9 | 61 | 2 |
| food | fish/shellfish | II | IIc | CC9 | ST9 | 74 | 1 |
| food | fish/shellfish | II | IIc | CC9 | ST9 | 99 | 1 |
| food | fish/shellfish | II | IIc | CC9 | ST9 | 184 | 4 |
| food | spices/herbs/vegetables | I | IIb | CC3 | ST3 | 517 | 1 |
| food | spices/herbs/vegetables | I | IIb | CC3 | ST3 | 518 | 1 |
| food | spices/herbs/vegetables | I | IIb | CC5 | ST5 | 616 | 1 |
| food | spices/herbs/vegetables | I | IVb | CC2 | ST2 | 515 | 1 |
| food | spices/herbs/vegetables | I | IVb | CC2 | ST2 | 731 | 1 |
| food | spices/herbs/vegetables | I | IVb | CC6 | ST6 | 327 | 2 |
| food | spices/herbs/vegetables | II | IIa | CC121 | ST121 | 35 | 1 |
| food | spices/herbs/vegetables | II | IIa | CC121 | ST121 | 141 | 1 |
| food | spices/herbs/vegetables | II | IIa | CC121 | ST121 | 143 | 1 |
| food | spices/herbs/vegetables | II | IIa | CC121 | ST121 | 149 | 1 |
| food | spices/herbs/vegetables | II | IIa | CC121 | ST121 | 151 | 1 |
| food | spices/herbs/vegetables | II | IIa | CC121 | ST121 | 278 | 1 |
| food | spices/herbs/vegetables | II | IIa | CC204 | ST204 | 112 | 1 |
| food | spices/herbs/vegetables | II | IIa | CC37 | ST37 | 273 | 1 |
| food | swine | I | IVb | CC1 | ST1 | 689 | 1 |
| food | swine | II | IIa | CC121 | ST121 | 142 | 1 |
| food | swine | II | IIa | CC121 | ST121 | 193 | 1 |
| food | swine | II | IIa | CC204 | ST204 | 112 | 1 |
| food | swine | II | IIa | CC475 | ST504 | 378 | 1 |
| food | swine | II | IIa | CC8 | ST2117 | 29 | 2 |
| food | swine | II | IIa | CC8 | ST8 | 793 | 1 |
| food | swine | II | IIa | (Missing) | (Missing) | 730 | 1 |
| food | swine | II | IIc | CC9 | ST580 | 224 | 1 |
| food | swine | II | IIc | CC9 | ST9 | 42 | 1 |
| food | swine | II | IIc | CC9 | ST9 | 794 | 1 |
| food | turkey | II | IIa | CC121 | ST121 | 52 | 1 |
| food | turkey | II | IIa | CC121 | ST121 | 218 | 4 |
| food | turkey | II | IIa | CC155 | ST155 | 293 | 2 |
| food | turkey | II | IIa | CC200 | ST200 | 215 | 1 |
| food | turkey | II | IIa | CC204 | ST204 | 173 | 1 |
| food | turkey | II | IIa | CC37 | ST37 | 31 | 1 |
| food | turkey | II | IIa | CC475 | ST504 | 290 | 1 |
| food | turkey | II | IIa | CC7 | ST7 | 274 | 5 |
| food | turkey | II | IIa | CC8 | ST8 | 20 | 13 |
| food | turkey | II | IIc | CC9 | ST9 | 2 | 1 |
| food | turkey | II | IIc | CC9 | ST9 | 47 | 1 |
| food | turkey | II | IIc | CC9 | (Missing) | 47 | 1 |
| food | small ruminants | I | IIb | CC224 | ST224 | 435 | 1 |
| food | small ruminants | I | IIb | CC489 | ST489 | 519 | 1 |
| food | small ruminants | I | IIb | CC5 | ST5 | 624 | 1 |
| food | small ruminants | I | IIb | CC59 | ST59 | 433 | 1 |
| food | small ruminants | I | IIb | CC77 | ST77 | 523 | 1 |
| food | small ruminants | I | IVb | CC1 | ST1 | 709 | 1 |
| food | small ruminants | I | IVb | CC2 | ST2 | 543 | 1 |
| food | small ruminants | I | IVb | CC220 | ST220 | 710 | 1 |
| food | small ruminants | I | IVb | CC4 | ST4 | 434 | 1 |
| food | small ruminants | I | IVb | CC4 | ST4 | 677 | 1 |
| food | small ruminants | I | IVb | CC6 | ST6 | 380 | 1 |
| food | small ruminants | I | IVb | CC6 | ST6 | 388 | 1 |
| food | small ruminants | II | IIa | CC11 | ST451 | 39 | 1 |
| food | small ruminants | II | IIa | CC121 | ST236 | 321 | 1 |
| food | small ruminants | II | IIa | CC14 | ST14 | 160 | 1 |
| food | small ruminants | II | IIa | CC14 | ST91 | 280 | 1 |
| food | small ruminants | II | IIa | CC18 | ST18 | 277 | 1 |
| food | small ruminants | II | IIa | CC18 | ST18 | 325 | 1 |
| food | small ruminants | II | IIa | CC20 | ST20 | 220 | 1 |
| food | small ruminants | II | IIa | CC26 | ST26 | 219 | 1 |
| food | small ruminants | II | IIa | CC321 | ST321 | 636 | 3 |
| food | small ruminants | II | IIa | CC37 | ST37 | 25 | 1 |
| food | small ruminants | II | IIa | CC37 | ST37 | 43 | 1 |
| food | small ruminants | II | IIa | CC37 | ST37 | 58 | 1 |
| food | small ruminants | II | IIa | CC37 | ST37 | 146 | 1 |
| food | small ruminants | II | IIa | CC37 | ST37 | 322 | 1 |
| food | small ruminants | II | IIa | CC8 | ST120 | 779 | 1 |
| food | small ruminants | II | IIa | CC8 | ST8 | 46 | 1 |
| food | small ruminants | II | IIa | CC8 | ST8 | 778 | 1 |
| food | small ruminants | II | IIc | CC9 | ST9 | 49 | 1 |
| food | game | I | IIb | CC426 | ST426 | 440 | 1 |
| food | game | I | IIb | CC5 | ST5 | 628 | 1 |
| food | game | II | IIa | CC18 | ST18 | 386 | 1 |
| food | game | II | IIa | CC204 | ST204 | 163 | 1 |
| food | amphibia | I | IVb | CC2 | ST2 | 540 | 1 |
| food | amphibia | I | IVb | CC4 | ST4 | 441 | 1 |
| food | (Missing) | I | IIb | CC489 | ST489 | 675 | 1 |
| food | (Missing) | II | IIa | CC8 | ST8 | 34 | 1 |
| human | (Missing) | I | IIb | CC224 | ST1343 | 466 | 1 |
| human | (Missing) | I | IIb | CC224 | ST224 | 397 | 1 |
| human | (Missing) | I | IIb | CC224 | ST224 | 402 | 1 |
| human | (Missing) | I | IIb | CC224 | ST224 | 412 | 3 |
| human | (Missing) | I | IIb | CC224 | ST224 | 416 | 2 |
| human | (Missing) | I | IIb | CC224 | ST224 | 487 | 1 |
| human | (Missing) | I | IIb | CC224 | ST224 | 721 | 1 |
| human | (Missing) | I | IIb | CC224 | ST224 | 727 | 1 |
| human | (Missing) | I | IIb | CC288 | ST288 | 544 | 1 |
| human | (Missing) | I | IIb | CC288 | ST330 | 549 | 2 |
| human | (Missing) | I | IIb | CC288 | ST330 | 598 | 1 |
| human | (Missing) | I | IIb | CC288 | ST330 | 757 | 1 |
| human | (Missing) | I | IIb | CC3 | ST1342 | 563 | 1 |
| human | (Missing) | I | IIb | CC3 | ST3 | 454 | 1 |
| human | (Missing) | I | IIb | CC3 | ST3 | 457 | 1 |
| human | (Missing) | I | IIb | CC3 | ST3 | 465 | 1 |
| human | (Missing) | I | IIb | CC3 | ST3 | 475 | 1 |
| human | (Missing) | I | IIb | CC3 | ST3 | 500 | 1 |
| human | (Missing) | I | IIb | CC3 | ST3 | 508 | 1 |
| human | (Missing) | I | IIb | CC3 | ST3 | 556 | 1 |
| human | (Missing) | I | IIb | CC3 | ST3 | 570 | 1 |
| human | (Missing) | I | IIb | CC3 | ST3 | 575 | 1 |
| human | (Missing) | I | IIb | CC3 | ST3 | 587 | 1 |
| human | (Missing) | I | IIb | CC3 | ST3 | 602 | 1 |
| human | (Missing) | I | IIb | CC3 | ST3 | 639 | 1 |
| human | (Missing) | I | IIb | CC3 | ST3 | 651 | 1 |
| human | (Missing) | I | IIb | CC3 | ST3 | 671 | 1 |
| human | (Missing) | I | IIb | CC3 | ST3 | 729 | 1 |
| human | (Missing) | I | IIb | CC3 | ST3 | 754 | 1 |
| human | (Missing) | I | IIb | CC3 | ST576 | 392 | 1 |
| human | (Missing) | I | IIb | CC379 | ST182 | 459 | 1 |
| human | (Missing) | I | IIb | CC379 | ST379 | 403 | 1 |
| human | (Missing) | I | IIb | CC379 | ST379 | 418 | 1 |
| human | (Missing) | I | IIb | CC379 | ST808 | 462 | 8 |
| human | (Missing) | I | IIb | CC392 | ST392 | 577 | 1 |
| human | (Missing) | I | IIb | CC489 | ST489 | 399 | 1 |
| human | (Missing) | I | IIb | CC489 | ST489 | 400 | 1 |
| human | (Missing) | I | IIb | CC5 | ST5 | 419 | 1 |
| human | (Missing) | I | IIb | CC5 | ST5 | 429 | 1 |
| human | (Missing) | I | IIb | CC5 | ST5 | 451 | 4 |
| human | (Missing) | I | IIb | CC5 | ST5 | 452 | 1 |
| human | (Missing) | I | IIb | CC5 | ST5 | 455 | 4 |
| human | (Missing) | I | IIb | CC5 | ST5 | 472 | 1 |
| human | (Missing) | I | IIb | CC5 | ST5 | 478 | 1 |
| human | (Missing) | I | IIb | CC5 | ST5 | 492 | 1 |
| human | (Missing) | I | IIb | CC5 | ST5 | 499 | 2 |
| human | (Missing) | I | IIb | CC5 | ST5 | 561 | 1 |
| human | (Missing) | I | IIb | CC5 | ST5 | 576 | 1 |
| human | (Missing) | I | IIb | CC5 | ST5 | 601 | 1 |
| human | (Missing) | I | IIb | CC5 | ST893 | 489 | 1 |
| human | (Missing) | I | IIb | CC517 | ST517 | 432 | 1 |
| human | (Missing) | I | IIb | CC59 | ST59 | 393 | 1 |
| human | (Missing) | I | IIb | CC59 | ST59 | 410 | 2 |
| human | (Missing) | I | IIb | CC59 | ST59 | 502 | 1 |
| human | (Missing) | I | IIb | CC59 | ST59 | 583 | 1 |
| human | (Missing) | I | IIb | CC59 | ST59 | 641 | 1 |
| human | (Missing) | I | IIb | CC59 | ST59 | 711 | 1 |
| human | (Missing) | I | IIb | CC59 | ST894 | 395 | 1 |
| human | (Missing) | I | IIb | CC77 | ST77 | 477 | 2 |
| human | (Missing) | I | IIb | CC77 | ST77 | 482 | 1 |
| human | (Missing) | I | IIb | CC77 | ST77 | 725 | 1 |
| human | (Missing) | I | IIb | CC77 | ST77 | 726 | 1 |
| human | (Missing) | I | IIb | CC87 | ST87 | 650 | 1 |
| human | (Missing) | I | IIb | CC87 | ST87 | 656 | 1 |
| human | (Missing) | I | IIb | CC87 | ST87 | 658 | 6 |
| human | (Missing) | I | IIb | CC87 | ST87 | 667 | 1 |
| human | (Missing) | I | IIb | ST773 | ST773 | 504 | 1 |
| human | (Missing) | I | IIb | ST773 | ST773 | 724 | 1 |
| human | (Missing) | I | IVb | CC1 | ST1 | 546 | 1 |
| human | (Missing) | I | IVb | CC1 | ST1 | 547 | 9 |
| human | (Missing) | I | IVb | CC1 | ST1 | 550 | 1 |
| human | (Missing) | I | IVb | CC1 | ST1 | 555 | 1 |
| human | (Missing) | I | IVb | CC1 | ST1 | 559 | 1 |
| human | (Missing) | I | IVb | CC1 | ST1 | 560 | 1 |
| human | (Missing) | I | IVb | CC1 | ST1 | 562 | 1 |
| human | (Missing) | I | IVb | CC1 | ST1 | 565 | 2 |
| human | (Missing) | I | IVb | CC1 | ST1 | 567 | 1 |
| human | (Missing) | I | IVb | CC1 | ST1 | 568 | 1 |
| human | (Missing) | I | IVb | CC1 | ST1 | 571 | 3 |
| human | (Missing) | I | IVb | CC1 | ST1 | 578 | 1 |
| human | (Missing) | I | IVb | CC1 | ST1 | 579 | 1 |
| human | (Missing) | I | IVb | CC1 | ST1 | 582 | 1 |
| human | (Missing) | I | IVb | CC1 | ST1 | 584 | 1 |
| human | (Missing) | I | IVb | CC1 | ST1 | 585 | 1 |
| human | (Missing) | I | IVb | CC1 | ST1 | 589 | 1 |
| human | (Missing) | I | IVb | CC1 | ST1 | 590 | 1 |
| human | (Missing) | I | IVb | CC1 | ST1 | 596 | 1 |
| human | (Missing) | I | IVb | CC1 | ST1 | 597 | 1 |
| human | (Missing) | I | IVb | CC1 | ST1 | 600 | 1 |
| human | (Missing) | I | IVb | CC1 | ST1 | 603 | 1 |
| human | (Missing) | I | IVb | CC1 | ST1 | 604 | 1 |
| human | (Missing) | I | IVb | CC1 | ST1 | 605 | 1 |
| human | (Missing) | I | IVb | CC1 | ST1 | 606 | 1 |
| human | (Missing) | I | IVb | CC1 | ST1 | 613 | 11 |
| human | (Missing) | I | IVb | CC1 | ST1 | 637 | 2 |
| human | (Missing) | I | IVb | CC1 | ST1 | 640 | 5 |
| human | (Missing) | I | IVb | CC1 | ST1 | 643 | 1 |
| human | (Missing) | I | IVb | CC1 | ST1 | 644 | 3 |
| human | (Missing) | I | IVb | CC1 | ST1 | 645 | 1 |
| human | (Missing) | I | IVb | CC1 | ST1 | 647 | 10 |
| human | (Missing) | I | IVb | CC1 | ST1 | 649 | 1 |
| human | (Missing) | I | IVb | CC1 | ST1 | 652 | 5 |
| human | (Missing) | I | IVb | CC1 | ST1 | 653 | 1 |
| human | (Missing) | I | IVb | CC1 | ST1 | 655 | 1 |
| human | (Missing) | I | IVb | CC1 | ST1 | 657 | 1 |
| human | (Missing) | I | IVb | CC1 | ST1 | 659 | 2 |
| human | (Missing) | I | IVb | CC1 | ST1 | 660 | 1 |
| human | (Missing) | I | IVb | CC1 | ST1 | 662 | 1 |
| human | (Missing) | I | IVb | CC1 | ST1 | 663 | 1 |
| human | (Missing) | I | IVb | CC1 | ST1 | 664 | 1 |
| human | (Missing) | I | IVb | CC1 | ST1 | 665 | 1 |
| human | (Missing) | I | IVb | CC1 | ST1 | 666 | 1 |
| human | (Missing) | I | IVb | CC1 | ST1 | 669 | 1 |
| human | (Missing) | I | IVb | CC1 | ST1 | 670 | 1 |
| human | (Missing) | I | IVb | CC1 | ST1 | 673 | 1 |
| human | (Missing) | I | IVb | CC1 | ST1 | 678 | 1 |
| human | (Missing) | I | IVb | CC1 | ST1 | 690 | 2 |
| human | (Missing) | I | IVb | CC1 | ST1 | 691 | 2 |
| human | (Missing) | I | IVb | CC1 | ST1 | 694 | 1 |
| human | (Missing) | I | IVb | CC1 | ST1 | 696 | 1 |
| human | (Missing) | I | IVb | CC1 | ST1 | 697 | 1 |
| human | (Missing) | I | IVb | CC1 | ST1 | 698 | 1 |
| human | (Missing) | I | IVb | CC1 | ST1 | 700 | 1 |
| human | (Missing) | I | IVb | CC1 | ST1 | 701 | 1 |
| human | (Missing) | I | IVb | CC1 | ST1 | 702 | 1 |
| human | (Missing) | I | IVb | CC1 | ST1 | 703 | 1 |
| human | (Missing) | I | IVb | CC1 | ST1 | 704 | 1 |
| human | (Missing) | I | IVb | CC1 | ST1 | 705 | 1 |
| human | (Missing) | I | IVb | CC1 | ST1 | 706 | 1 |
| human | (Missing) | I | IVb | CC1 | ST1 | 712 | 1 |
| human | (Missing) | I | IVb | CC1 | ST1 | 713 | 1 |
| human | (Missing) | I | IVb | CC1 | ST1 | 714 | 1 |
| human | (Missing) | I | IVb | CC1 | ST1 | 715 | 2 |
| human | (Missing) | I | IVb | CC1 | ST1 | 732 | 1 |
| human | (Missing) | I | IVb | CC1 | ST1 | 743 | 1 |
| human | (Missing) | I | IVb | CC1 | ST1 | 744 | 1 |
| human | (Missing) | I | IVb | CC1 | ST1 | 746 | 1 |
| human | (Missing) | I | IVb | CC1 | ST1 | 750 | 1 |
| human | (Missing) | I | IVb | CC1 | ST1 | 758 | 1 |
| human | (Missing) | I | IVb | CC1 | ST1345 | 654 | 1 |
| human | (Missing) | I | IVb | CC1 | ST1575 | 693 | 1 |
| human | (Missing) | I | IVb | CC1 | ST515 | 638 | 2 |
| human | (Missing) | I | IVb | CC183 | ST382 | 490 | 1 |
| human | (Missing) | I | IVb | CC2 | ST2 | 396 | 2 |
| human | (Missing) | I | IVb | CC2 | ST2 | 408 | 1 |
| human | (Missing) | I | IVb | CC2 | ST2 | 415 | 1 |
| human | (Missing) | I | IVb | CC2 | ST2 | 424 | 1 |
| human | (Missing) | I | IVb | CC2 | ST2 | 425 | 1 |
| human | (Missing) | I | IVb | CC2 | ST2 | 428 | 1 |
| human | (Missing) | I | IVb | CC2 | ST2 | 453 | 1 |
| human | (Missing) | I | IVb | CC2 | ST2 | 460 | 1 |
| human | (Missing) | I | IVb | CC2 | ST2 | 461 | 1 |
| human | (Missing) | I | IVb | CC2 | ST2 | 463 | 1 |
| human | (Missing) | I | IVb | CC2 | ST2 | 464 | 1 |
| human | (Missing) | I | IVb | CC2 | ST2 | 467 | 1 |
| human | (Missing) | I | IVb | CC2 | ST2 | 469 | 1 |
| human | (Missing) | I | IVb | CC2 | ST2 | 473 | 1 |
| human | (Missing) | I | IVb | CC2 | ST2 | 476 | 1 |
| human | (Missing) | I | IVb | CC2 | ST2 | 480 | 1 |
| human | (Missing) | I | IVb | CC2 | ST2 | 481 | 1 |
| human | (Missing) | I | IVb | CC2 | ST2 | 483 | 1 |
| human | (Missing) | I | IVb | CC2 | ST2 | 484 | 1 |
| human | (Missing) | I | IVb | CC2 | ST2 | 488 | 1 |
| human | (Missing) | I | IVb | CC2 | ST2 | 491 | 2 |
| human | (Missing) | I | IVb | CC2 | ST2 | 493 | 3 |
| human | (Missing) | I | IVb | CC2 | ST2 | 495 | 1 |
| human | (Missing) | I | IVb | CC2 | ST2 | 497 | 1 |
| human | (Missing) | I | IVb | CC2 | ST2 | 501 | 1 |
| human | (Missing) | I | IVb | CC2 | ST2 | 503 | 1 |
| human | (Missing) | I | IVb | CC2 | ST2 | 505 | 1 |
| human | (Missing) | I | IVb | CC2 | ST2 | 506 | 1 |
| human | (Missing) | I | IVb | CC2 | ST2 | 507 | 1 |
| human | (Missing) | I | IVb | CC2 | ST2 | 509 | 1 |
| human | (Missing) | I | IVb | CC2 | ST2 | 545 | 1 |
| human | (Missing) | I | IVb | CC2 | ST2 | 548 | 4 |
| human | (Missing) | I | IVb | CC2 | ST2 | 551 | 1 |
| human | (Missing) | I | IVb | CC2 | ST2 | 552 | 1 |
| human | (Missing) | I | IVb | CC2 | ST2 | 553 | 1 |
| human | (Missing) | I | IVb | CC2 | ST2 | 554 | 1 |
| human | (Missing) | I | IVb | CC2 | ST2 | 557 | 1 |
| human | (Missing) | I | IVb | CC2 | ST2 | 564 | 1 |
| human | (Missing) | I | IVb | CC2 | ST2 | 566 | 1 |
| human | (Missing) | I | IVb | CC2 | ST2 | 573 | 1 |
| human | (Missing) | I | IVb | CC2 | ST2 | 580 | 1 |
| human | (Missing) | I | IVb | CC2 | ST2 | 581 | 1 |
| human | (Missing) | I | IVb | CC2 | ST2 | 588 | 1 |
| human | (Missing) | I | IVb | CC2 | ST2 | 591 | 1 |
| human | (Missing) | I | IVb | CC2 | ST2 | 592 | 1 |
| human | (Missing) | I | IVb | CC2 | ST2 | 594 | 1 |
| human | (Missing) | I | IVb | CC2 | ST2 | 599 | 1 |
| human | (Missing) | I | IVb | CC2 | ST2 | 607 | 1 |
| human | (Missing) | I | IVb | CC2 | ST2 | 608 | 1 |
| human | (Missing) | I | IVb | CC2 | ST2 | 610 | 1 |
| human | (Missing) | I | IVb | CC2 | ST2 | 611 | 11 |
| human | (Missing) | I | IVb | CC2 | ST2 | 612 | 1 |
| human | (Missing) | I | IVb | CC2 | ST2 | 633 | 1 |
| human | (Missing) | I | IVb | CC2 | ST2 | 642 | 1 |
| human | (Missing) | I | IVb | CC2 | ST2 | 646 | 1 |
| human | (Missing) | I | IVb | CC2 | ST2 | 648 | 1 |
| human | (Missing) | I | IVb | CC2 | ST2 | 661 | 1 |
| human | (Missing) | I | IVb | CC2 | ST2 | 668 | 1 |
| human | (Missing) | I | IVb | CC2 | ST2 | 672 | 1 |
| human | (Missing) | I | IVb | CC2 | ST2 | 707 | 1 |
| human | (Missing) | I | IVb | CC2 | ST2 | 728 | 1 |
| human | (Missing) | I | IVb | CC2 | ST2 | 753 | 1 |
| human | (Missing) | I | IVb | CC2 | ST2 | 759 | 1 |
| human | (Missing) | I | IVb | CC2 | ST809 | 498 | 1 |
| human | (Missing) | I | IVb | CC2 | ST809 | 558 | 1 |
| human | (Missing) | I | IVb | CC217 | ST217 | 367 | 1 |
| human | (Missing) | I | IVb | CC217 | ST217 | 430 | 1 |
| human | (Missing) | I | IVb | CC218 | ST218 | 458 | 1 |
| human | (Missing) | I | IVb | CC218 | ST218 | 474 | 1 |
| human | (Missing) | I | IVb | CC218 | ST218 | 511 | 1 |
| human | (Missing) | I | IVb | CC218 | ST218 | 699 | 1 |
| human | (Missing) | I | IVb | CC315 | ST194 | 692 | 1 |
| human | (Missing) | I | IVb | CC315 | ST194 | 695 | 1 |
| human | (Missing) | I | IVb | CC315 | ST194 | 722 | 1 |
| human | (Missing) | I | IVb | CC388 | ST388 | 330 | 1 |
| human | (Missing) | I | IVb | CC388 | ST388 | 348 | 2 |
| human | (Missing) | I | IVb | CC388 | ST388 | 356 | 1 |
| human | (Missing) | I | IVb | CC388 | ST388 | 366 | 1 |
| human | (Missing) | I | IVb | CC389 | ST389 | 718 | 1 |
| human | (Missing) | I | IVb | CC389 | ST389 | 719 | 1 |
| human | (Missing) | I | IVb | CC389 | ST389 | 749 | 1 |
| human | (Missing) | I | IVb | CC4 | ST219 | 496 | 1 |
| human | (Missing) | I | IVb | CC4 | ST219 | 513 | 1 |
| human | (Missing) | I | IVb | CC4 | ST4 | 405 | 1 |
| human | (Missing) | I | IVb | CC4 | ST4 | 420 | 1 |
| human | (Missing) | I | IVb | CC4 | ST4 | 422 | 1 |
| human | (Missing) | I | IVb | CC4 | ST4 | 423 | 1 |
| human | (Missing) | I | IVb | CC4 | ST4 | 431 | 2 |
| human | (Missing) | I | IVb | CC4 | ST4 | 470 | 1 |
| human | (Missing) | I | IVb | CC4 | ST4 | 471 | 1 |
| human | (Missing) | I | IVb | CC4 | ST4 | 479 | 1 |
| human | (Missing) | I | IVb | CC4 | ST4 | 485 | 1 |
| human | (Missing) | I | IVb | CC4 | ST4 | 486 | 1 |
| human | (Missing) | I | IVb | CC4 | ST4 | 494 | 1 |
| human | (Missing) | I | IVb | CC4 | ST4 | 510 | 1 |
| human | (Missing) | I | IVb | CC4 | ST4 | 512 | 1 |
| human | (Missing) | I | IVb | CC4 | ST4 | 593 | 1 |
| human | (Missing) | I | IVb | CC4 | ST4 | 609 | 1 |
| human | (Missing) | I | IVb | CC4 | ST4 | 741 | 1 |
| human | (Missing) | I | IVb | CC54 | ST54 | 398 | 1 |
| human | (Missing) | I | IVb | CC54 | ST54 | 413 | 1 |
| human | (Missing) | I | IVb | CC54 | ST54 | 751 | 1 |
| human | (Missing) | I | IVb | CC6 | ST6 | 327 | 50 |
| human | (Missing) | I | IVb | CC6 | ST6 | 329 | 1 |
| human | (Missing) | I | IVb | CC6 | ST6 | 333 | 3 |
| human | (Missing) | I | IVb | CC6 | ST6 | 334 | 5 |
| human | (Missing) | I | IVb | CC6 | ST6 | 335 | 6 |
| human | (Missing) | I | IVb | CC6 | ST6 | 337 | 1 |
| human | (Missing) | I | IVb | CC6 | ST6 | 338 | 1 |
| human | (Missing) | I | IVb | CC6 | ST6 | 339 | 5 |
| human | (Missing) | I | IVb | CC6 | ST6 | 340 | 2 |
| human | (Missing) | I | IVb | CC6 | ST6 | 341 | 3 |
| human | (Missing) | I | IVb | CC6 | ST6 | 342 | 1 |
| human | (Missing) | I | IVb | CC6 | ST6 | 343 | 2 |
| human | (Missing) | I | IVb | CC6 | ST6 | 344 | 1 |
| human | (Missing) | I | IVb | CC6 | ST6 | 345 | 1 |
| human | (Missing) | I | IVb | CC6 | ST6 | 346 | 1 |
| human | (Missing) | I | IVb | CC6 | ST6 | 350 | 2 |
| human | (Missing) | I | IVb | CC6 | ST6 | 352 | 1 |
| human | (Missing) | I | IVb | CC6 | ST6 | 354 | 1 |
| human | (Missing) | I | IVb | CC6 | ST6 | 355 | 1 |
| human | (Missing) | I | IVb | CC6 | ST6 | 357 | 1 |
| human | (Missing) | I | IVb | CC6 | ST6 | 358 | 1 |
| human | (Missing) | I | IVb | CC6 | ST6 | 359 | 1 |
| human | (Missing) | I | IVb | CC6 | ST6 | 361 | 1 |
| human | (Missing) | I | IVb | CC6 | ST6 | 362 | 1 |
| human | (Missing) | I | IVb | CC6 | ST6 | 364 | 1 |
| human | (Missing) | I | IVb | CC6 | ST6 | 365 | 1 |
| human | (Missing) | I | IVb | CC6 | ST6 | 369 | 1 |
| human | (Missing) | I | IVb | CC6 | ST6 | 389 | 2 |
| human | (Missing) | I | IVb | CC6 | ST6 | 390 | 1 |
| human | (Missing) | I | IVb | CC6 | ST6 | 391 | 3 |
| human | (Missing) | I | IVb | CC6 | ST6 | 394 | 4 |
| human | (Missing) | I | IVb | CC6 | ST6 | 404 | 1 |
| human | (Missing) | I | IVb | CC6 | ST6 | 406 | 1 |
| human | (Missing) | I | IVb | CC6 | ST6 | 409 | 1 |
| human | (Missing) | I | IVb | CC6 | ST6 | 414 | 2 |
| human | (Missing) | I | IVb | CC6 | ST6 | 421 | 1 |
| human | (Missing) | I | IVb | CC6 | ST6 | 426 | 1 |
| human | (Missing) | I | IVb | CC6 | ST6 | 569 | 1 |
| human | (Missing) | I | IVb | CC6 | ST6 | 574 | 1 |
| human | (Missing) | I | IVb | CC6 | ST6 | 742 | 1 |
| human | (Missing) | I | IVb | (Missing) | (Missing) | 614 | 1 |
| human | (Missing) | II | (Missing) | CC155 | ST155 | 293 | 1 |
| human | (Missing) | II | (Missing) | CC8 | ST8 | 760 | 1 |
| human | (Missing) | II | IIa | CC101 | ST101 | 353 | 1 |
| human | (Missing) | II | IIa | CC101 | ST101 | 363 | 1 |
| human | (Missing) | II | IIa | CC101 | ST101 | 401 | 2 |
| human | (Missing) | II | IIa | CC101 | ST101 | 407 | 1 |
| human | (Missing) | II | IIa | CC101 | ST101 | 411 | 2 |
| human | (Missing) | II | IIa | CC101 | ST101 | 427 | 1 |
| human | (Missing) | II | IIa | CC101 | ST101 | 572 | 1 |
| human | (Missing) | II | IIa | CC101 | ST38 | 674 | 2 |
| human | (Missing) | II | IIa | CC11 | ST451 | 6 | 1 |
| human | (Missing) | II | IIa | CC11 | ST451 | 22 | 1 |
| human | (Missing) | II | IIa | CC11 | ST451 | 33 | 1 |
| human | (Missing) | II | IIa | CC11 | ST451 | 124 | 1 |
| human | (Missing) | II | IIa | CC11 | ST451 | 134 | 1 |
| human | (Missing) | II | IIa | CC11 | ST451 | 216 | 1 |
| human | (Missing) | II | IIa | CC11 | ST451 | 217 | 1 |
| human | (Missing) | II | IIa | CC121 | ST121 | 102 | 1 |
| human | (Missing) | II | IIa | CC121 | ST121 | 107 | 1 |
| human | (Missing) | II | IIa | CC121 | ST121 | 110 | 1 |
| human | (Missing) | II | IIa | CC121 | ST121 | 130 | 1 |
| human | (Missing) | II | IIa | CC121 | ST121 | 199 | 1 |
| human | (Missing) | II | IIa | CC121 | ST121 | 204 | 1 |
| human | (Missing) | II | IIa | CC14 | ST14 | 109 | 9 |
| human | (Missing) | II | IIa | CC14 | ST14 | 111 | 2 |
| human | (Missing) | II | IIa | CC14 | ST14 | 198 | 1 |
| human | (Missing) | II | IIa | CC14 | ST206 | 127 | 1 |
| human | (Missing) | II | IIa | CC14 | ST399 | 122 | 1 |
| human | (Missing) | II | IIa | CC14 | ST399 | 133 | 1 |
| human | (Missing) | II | IIa | CC14 | ST399 | 208 | 16 |
| human | (Missing) | II | IIa | CC14 | ST91 | 258 | 1 |
| human | (Missing) | II | IIa | CC14 | ST91 | 263 | 1 |
| human | (Missing) | II | IIa | CC14 | ST91 | 267 | 1 |
| human | (Missing) | II | IIa | CC14 | ST91 | 295 | 1 |
| human | (Missing) | II | IIa | CC14 | ST91 | 297 | 1 |
| human | (Missing) | II | IIa | CC14 | ST91 | 298 | 1 |
| human | (Missing) | II | IIa | CC14 | ST91 | 331 | 1 |
| human | (Missing) | II | IIa | CC14 | ST91 | 347 | 1 |
| human | (Missing) | II | IIa | CC14 | ST91 | 595 | 1 |
| human | (Missing) | II | IIa | CC155 | ST155 | 292 | 1 |
| human | (Missing) | II | IIa | CC155 | ST155 | 293 | 3 |
| human | (Missing) | II | IIa | CC155 | ST155 | 294 | 16 |
| human | (Missing) | II | IIa | CC155 | ST155 | 299 | 3 |
| human | (Missing) | II | IIa | CC155 | ST155 | 328 | 4 |
| human | (Missing) | II | IIa | CC155 | ST155 | 336 | 9 |
| human | (Missing) | II | IIa | CC155 | ST155 | 349 | 1 |
| human | (Missing) | II | IIa | CC155 | ST155 | 456 | 1 |
| human | (Missing) | II | IIa | CC155 | ST155 | 468 | 1 |
| human | (Missing) | II | IIa | CC155 | ST994 | 291 | 2 |
| human | (Missing) | II | IIa | CC177 | ST177 | 17 | 1 |
| human | (Missing) | II | IIa | CC18 | ST18 | 197 | 2 |
| human | (Missing) | II | IIa | CC18 | ST18 | 209 | 2 |
| human | (Missing) | II | IIa | CC18 | ST18 | 210 | 1 |
| human | (Missing) | II | IIa | CC18 | ST18 | 257 | 1 |
| human | (Missing) | II | IIa | CC18 | ST18 | 260 | 1 |
| human | (Missing) | II | IIa | CC18 | ST18 | 262 | 1 |
| human | (Missing) | II | IIa | CC18 | ST18 | 266 | 2 |
| human | (Missing) | II | IIa | CC18 | ST18 | 270 | 1 |
| human | (Missing) | II | IIa | CC18 | ST18 | 300 | 1 |
| human | (Missing) | II | IIa | CC18 | ST18 | 370 | 1 |
| human | (Missing) | II | IIa | CC19 | ST173 | 253 | 8 |
| human | (Missing) | II | IIa | CC19 | ST173 | 259 | 6 |
| human | (Missing) | II | IIa | CC19 | ST173 | 514 | 1 |
| human | (Missing) | II | IIa | CC19 | ST398 | 254 | 1 |
| human | (Missing) | II | IIa | CC19 | ST398 | 303 | 1 |
| human | (Missing) | II | IIa | CC20 | ST20 | 137 | 1 |
| human | (Missing) | II | IIa | CC20 | ST20 | 196 | 1 |
| human | (Missing) | II | IIa | CC20 | ST20 | 211 | 1 |
| human | (Missing) | II | IIa | CC200 | ST200 | 202 | 1 |
| human | (Missing) | II | IIa | CC200 | ST200 | 215 | 1 |
| human | (Missing) | II | IIa | CC200 | ST200 | 417 | 1 |
| human | (Missing) | II | IIa | CC204 | ST204 | 105 | 1 |
| human | (Missing) | II | IIa | CC204 | ST204 | 106 | 5 |
| human | (Missing) | II | IIa | CC204 | ST204 | 112 | 2 |
| human | (Missing) | II | IIa | CC204 | ST204 | 131 | 1 |
| human | (Missing) | II | IIa | CC204 | ST204 | 163 | 1 |
| human | (Missing) | II | IIa | CC204 | ST204 | 203 | 2 |
| human | (Missing) | II | IIa | CC21 | ST21 | 15 | 1 |
| human | (Missing) | II | IIa | CC21 | ST21 | 26 | 1 |
| human | (Missing) | II | IIa | CC21 | ST21 | 121 | 1 |
| human | (Missing) | II | IIa | CC26 | ST26 | 206 | 1 |
| human | (Missing) | II | IIa | CC26 | ST26 | 371 | 1 |
| human | (Missing) | II | IIa | CC29 | ST1344 | 761 | 1 |
| human | (Missing) | II | IIa | CC29 | ST29 | 12 | 2 |
| human | (Missing) | II | IIa | CC29 | ST427 | 764 | 1 |
| human | (Missing) | II | IIa | CC31 | ST325 | 360 | 1 |
| human | (Missing) | II | IIa | CC37 | ST37 | 5 | 1 |
| human | (Missing) | II | IIa | CC37 | ST37 | 8 | 1 |
| human | (Missing) | II | IIa | CC37 | ST37 | 9 | 1 |
| human | (Missing) | II | IIa | CC37 | ST37 | 13 | 1 |
| human | (Missing) | II | IIa | CC37 | ST37 | 19 | 2 |
| human | (Missing) | II | IIa | CC37 | ST37 | 21 | 1 |
| human | (Missing) | II | IIa | CC37 | ST37 | 25 | 1 |
| human | (Missing) | II | IIa | CC37 | ST37 | 28 | 1 |
| human | (Missing) | II | IIa | CC37 | ST37 | 30 | 1 |
| human | (Missing) | II | IIa | CC37 | ST37 | 31 | 1 |
| human | (Missing) | II | IIa | CC37 | ST37 | 104 | 1 |
| human | (Missing) | II | IIa | CC37 | ST37 | 114 | 1 |
| human | (Missing) | II | IIa | CC37 | ST37 | 115 | 1 |
| human | (Missing) | II | IIa | CC37 | ST37 | 117 | 1 |
| human | (Missing) | II | IIa | CC37 | ST37 | 120 | 1 |
| human | (Missing) | II | IIa | CC37 | ST37 | 123 | 1 |
| human | (Missing) | II | IIa | CC37 | ST37 | 125 | 1 |
| human | (Missing) | II | IIa | CC37 | ST37 | 126 | 7 |
| human | (Missing) | II | IIa | CC37 | ST37 | 207 | 1 |
| human | (Missing) | II | IIa | CC37 | ST37 | 368 | 1 |
| human | (Missing) | II | IIa | CC403 | ST403 | 118 | 2 |
| human | (Missing) | II | IIa | CC412 | ST412 | 252 | 1 |
| human | (Missing) | II | IIa | CC412 | ST412 | 261 | 1 |
| human | (Missing) | II | IIa | CC412 | ST412 | 265 | 1 |
| human | (Missing) | II | IIa | CC412 | ST412 | 296 | 1 |
| human | (Missing) | II | IIa | CC415 | ST394 | 116 | 1 |
| human | (Missing) | II | IIa | CC475 | ST504 | 306 | 1 |
| human | (Missing) | II | IIa | CC475 | ST504 | 332 | 1 |
| human | (Missing) | II | IIa | CC475 | ST504 | 351 | 1 |
| human | (Missing) | II | IIa | CC7 | ST7 | 195 | 1 |
| human | (Missing) | II | IIa | CC7 | ST7 | 205 | 1 |
| human | (Missing) | II | IIa | CC7 | ST7 | 212 | 1 |
| human | (Missing) | II | IIa | CC7 | ST7 | 213 | 6 |
| human | (Missing) | II | IIa | CC7 | ST7 | 214 | 1 |
| human | (Missing) | II | IIa | CC7 | ST7 | 264 | 2 |
| human | (Missing) | II | IIa | CC7 | ST7 | 268 | 1 |
| human | (Missing) | II | IIa | CC7 | ST7 | 269 | 1 |
| human | (Missing) | II | IIa | CC8 | ST120 | 738 | 1 |
| human | (Missing) | II | IIa | CC8 | ST16 | 10 | 1 |
| human | (Missing) | II | IIa | CC8 | ST16 | 773 | 1 |
| human | (Missing) | II | IIa | CC8 | ST2117 | 29 | 3 |
| human | (Missing) | II | IIa | CC8 | ST8 | 14 | 1 |
| human | (Missing) | II | IIa | CC8 | ST8 | 20 | 1 |
| human | (Missing) | II | IIa | CC8 | ST8 | 23 | 4 |
| human | (Missing) | II | IIa | CC8 | ST8 | 24 | 1 |
| human | (Missing) | II | IIa | CC8 | ST8 | 27 | 6 |
| human | (Missing) | II | IIa | CC8 | ST8 | 32 | 3 |
| human | (Missing) | II | IIa | CC8 | ST8 | 46 | 4 |
| human | (Missing) | II | IIa | CC8 | ST8 | 119 | 1 |
| human | (Missing) | II | IIa | CC8 | ST8 | 128 | 1 |
| human | (Missing) | II | IIa | CC8 | ST8 | 132 | 1 |
| human | (Missing) | II | IIa | CC8 | ST8 | 135 | 2 |
| human | (Missing) | II | IIa | CC8 | ST8 | 136 | 3 |
| human | (Missing) | II | IIa | CC8 | ST8 | 250 | 1 |
| human | (Missing) | II | IIa | CC8 | ST8 | 251 | 1 |
| human | (Missing) | II | IIa | CC8 | ST8 | 271 | 3 |
| human | (Missing) | II | IIa | CC8 | ST8 | 717 | 1 |
| human | (Missing) | II | IIa | CC8 | ST8 | 723 | 1 |
| human | (Missing) | II | IIa | CC8 | ST8 | 745 | 1 |
| human | (Missing) | II | IIa | CC8 | ST8 | 760 | 2 |
| human | (Missing) | II | IIa | CC8 | ST8 | 762 | 2 |
| human | (Missing) | II | IIa | CC8 | ST8 | 763 | 1 |
| human | (Missing) | II | IIa | CC8 | ST8 | 765 | 1 |
| human | (Missing) | II | IIa | CC8 | ST8 | 766 | 1 |
| human | (Missing) | II | IIa | CC8 | ST8 | 767 | 2 |
| human | (Missing) | II | IIa | CC8 | ST8 | 768 | 1 |
| human | (Missing) | II | IIa | CC8 | ST8 | 769 | 1 |
| human | (Missing) | II | IIa | CC8 | ST8 | 770 | 1 |
| human | (Missing) | II | IIa | CC8 | ST8 | 771 | 1 |
| human | (Missing) | II | IIa | CC8 | ST8 | 772 | 1 |
| human | (Missing) | II | IIa | CC8 | ST8 | 774 | 1 |
| human | (Missing) | II | IIa | CC89 | ST391 | 586 | 1 |
| human | (Missing) | II | IIa | CC9 | ST9 | 184 | 1 |
| human | (Missing) | II | IIa | CC90 | ST222 | 201 | 1 |
| human | (Missing) | II | IIa | CC90 | ST425 | 255 | 1 |
| human | (Missing) | II | IIa | ST36 | ST36 | 18 | 1 |
| human | (Missing) | II | IIa | ST585 | ST585 | 103 | 1 |
| human | (Missing) | II | IIa | (Missing) | (Missing) | 256 | 1 |
| human | (Missing) | II | IIa | (Missing) | (Missing) | 301 | 1 |
| human | (Missing) | II | IIa | (Missing) | (Missing) | 302 | 1 |
| human | (Missing) | II | IIa | (Missing) | (Missing) | 720 | 1 |
| human | (Missing) | II | IIc | CC9 | ST9 | 4 | 1 |
| human | (Missing) | II | IIc | CC9 | ST9 | 7 | 1 |
| human | (Missing) | II | IIc | CC9 | ST9 | 11 | 1 |
| human | (Missing) | II | IIc | CC9 | ST9 | 16 | 5 |
| human | (Missing) | II | IIc | CC9 | ST9 | 42 | 8 |
| human | (Missing) | II | IIc | CC9 | ST9 | 108 | 1 |
| human | (Missing) | II | IIc | CC9 | ST9 | 113 | 1 |
| human | (Missing) | II | IIc | CC9 | ST9 | 129 | 1 |
| human | (Missing) | II | IIc | CC9 | ST9 | 200 | 1 |

**Supplementary Table 2**: Odds ratios and p-values for the differential distribution of the various genotypes among the food types; a false discovery rate correction has been used for the p-values; cluster_s7=clusters have been delineated using single-linkage hierarchical clustering with a threshold of seven.

| food_source | genotype | OR | pval_fdr | typing |
| --- | --- | --- | --- | --- |
| trout | I | 3.3 | 0.0006408 | lineage |
| trout | II | 0.3 | 0.0006408 | lineage |
| salmon | II | 8.5 | 0.0000731 | lineage |
| salmon | I | 0.1 | 0.0000731 | lineage |
| spices/herbs/vegetables | I | 3.7 | 0.0147549 | lineage |
| spices/herbs/vegetables | II | 0.3 | 0.0147549 | lineage |
| amphibia | I | Inf | 0.0377451 | lineage |
| amphibia | II | 0.0 | 0.0377451 | lineage |
| feces | I | 3.9 | 0.0049882 | lineage |
| feces | II | 0.3 | 0.0049882 | lineage |
| fresh meat | IIc | 1.6 | 0.0256168 | serogroup |
| fresh meat | IVb | 0.5 | 0.0279054 | serogroup |
| trout | IVb | 7.2 | 0.0000006 | serogroup |
| salmon | IIa | 2.8 | 0.0019422 | serogroup |
| salmon | IVb | 0.2 | 0.0400116 | serogroup |
| salmon | IIb | 0.0 | 0.0105110 | serogroup |
| herring | IIa | 11.8 | 0.0000371 | serogroup |
| herring | IIc | 0.0 | 0.0007996 | serogroup |
| fresh meat | CC5 | 3.4 | 0.0407337 | CC |
| fresh meat | CC9 | 1.7 | 0.0407337 | CC |
| fresh meat | CC121 | 0.4 | 0.0058741 | CC |
| processed meat | CC200 | 14.7 | 0.0088006 | CC |
| trout | CC1 | 23.4 | 0.0000000 | CC |
| salmon | CC19 | 44.8 | 0.0069570 | CC |
| salmon | CC7 | 8.3 | 0.0000001 | CC |
| salmon | CC31 | 7.7 | 0.0171307 | CC |
| herring | CC193 | Inf | 0.0017792 | CC |
| herring | CC121 | 10.0 | 0.0000002 | CC |
| herring | CC9 | 0.0 | 0.0066577 | CC |
| shrimp | CC321 | 45.5 | 0.0104664 | CC |
| fresh meat | 213 | 0.0 | 0.0011266 | cluster_s7 |
| fresh meat | 613 | 0.0 | 0.0023593 | cluster_s7 |
| processed meat | 71 | Inf | 0.0199290 | cluster_s7 |
| processed meat | 320 | Inf | 0.0085559 | cluster_s7 |
| trout | 67 | Inf | 0.0080169 | cluster_s7 |
| trout | 83 | Inf | 0.0011602 | cluster_s7 |
| trout | 190 | Inf | 0.0080169 | cluster_s7 |
| trout | 613 | 211.6 | 0.0000000 | cluster_s7 |
| trout | 107 | 79.2 | 0.0027914 | cluster_s7 |
| trout | 3 | 34.1 | 0.0018116 | cluster_s7 |
| salmon | 138 | Inf | 0.0007406 | cluster_s7 |
| salmon | 184 | Inf | 0.0045748 | cluster_s7 |
| salmon | 213 | Inf | 0.0000000 | cluster_s7 |
| salmon | 253 | Inf | 0.0045748 | cluster_s7 |
| salmon | 516 | 44.8 | 0.0171142 | cluster_s7 |
| herring | 139 | Inf | 0.0000000 | cluster_s7 |
| herring | 156 | Inf | 0.0000141 | cluster_s7 |
| herring | 324 | Inf | 0.0054710 | cluster_s7 |
| herring | 615 | Inf | 0.0054710 | cluster_s7 |
| shrimp | 307 | Inf | 0.0004090 | cluster_s7 |

**Supplementary Table 3**: Odds ratios and p-values for the differential distribution of the various genotypes among the food categories; a false discovery rate correction has been used for the p-values.

| food_source | genotype | OR | pval_fdr | typing |
| --- | --- | --- | --- | --- |
| chicken | IIa | 1.4 | 0.0428196 | serogroup |
| chicken | IVb | 0.4 | 0.0101840 | serogroup |
| cattle | IIc | 2.8 | 0.0000000 | serogroup |
| cattle | IIb | 2.7 | 0.0003651 | serogroup |
| cattle | IIa | 0.3 | 0.0000000 | serogroup |
| fish/shellfish | IIa | 2.0 | 0.0008523 | serogroup |
| fish/shellfish | IVb | 1.8 | 0.0415789 | serogroup |
| fish/shellfish | IIc | 0.4 | 0.0009355 | serogroup |
| fish/shellfish | IIb | 0.1 | 0.0002099 | serogroup |
| turkey | IIa | 7.8 | 0.0001604 | serogroup |
| small ruminants | IIc | 0.1 | 0.0123151 | serogroup |
| chicken | CC199 | Inf | 0.0000000 | CC |
| chicken | CC204 | 3.9 | 0.0227577 | CC |
| cattle | CC224 | 19.3 | 0.0056991 | CC |
| cattle | CC200 | 6.4 | 0.0361710 | CC |
| cattle | CC9 | 2.9 | 0.0000002 | CC |
| cattle | CC121 | 0.3 | 0.0008031 | CC |
| cattle | CC204 | 0.1 | 0.0251341 | CC |
| cattle | CC7 | 0.1 | 0.0008031 | CC |
| cattle | CC199 | 0.0 | 0.0027571 | CC |
| fish/shellfish | CC193 | Inf | 0.0469027 | CC |
| fish/shellfish | CC19 | 15.3 | 0.0456669 | CC |
| fish/shellfish | CC31 | 7.9 | 0.0022952 | CC |
| fish/shellfish | CC1 | 4.3 | 0.0016579 | CC |
| fish/shellfish | CC121 | 3.3 | 0.0000033 | CC |
| fish/shellfish | CC7 | 2.7 | 0.0080859 | CC |
| fish/shellfish | CC9 | 0.4 | 0.0056822 | CC |
| fish/shellfish | CC5 | 0.1 | 0.0293208 | CC |
| fish/shellfish | CC199 | 0.0 | 0.0212554 | CC |
| turkey | CC8 | 13.6 | 0.0000003 | CC |
| chicken | 53 | Inf | 0.0173475 | cluster_s7 |
| chicken | 145 | Inf | 0.0000000 | cluster_s7 |
| chicken | 112 | 9.7 | 0.0252027 | cluster_s7 |
| chicken | 2 | 7.7 | 0.0000000 | cluster_s7 |
| chicken | 274 | 5.2 | 0.0473187 | cluster_s7 |
| chicken | 213 | 0.0 | 0.0160305 | cluster_s7 |
| chicken | 613 | 0.0 | 0.0173475 | cluster_s7 |
| cattle | 71 | Inf | 0.0233261 | cluster_s7 |
| cattle | 81 | Inf | 0.0233261 | cluster_s7 |
| cattle | 320 | Inf | 0.0185937 | cluster_s7 |
| cattle | 416 | Inf | 0.0233261 | cluster_s7 |
| cattle | 530 | Inf | 0.0001787 | cluster_s7 |
| cattle | 145 | 0.0 | 0.0233261 | cluster_s7 |
| fish/shellfish | 3 | Inf | 0.0003081 | cluster_s7 |
| fish/shellfish | 107 | Inf | 0.0180988 | cluster_s7 |
| fish/shellfish | 138 | Inf | 0.0180988 | cluster_s7 |
| fish/shellfish | 139 | Inf | 0.0000830 | cluster_s7 |
| fish/shellfish | 156 | Inf | 0.0180988 | cluster_s7 |
| fish/shellfish | 213 | Inf | 0.0000000 | cluster_s7 |
| fish/shellfish | 516 | Inf | 0.0180988 | cluster_s7 |
| fish/shellfish | 613 | Inf | 0.0000000 | cluster_s7 |
| turkey | 218 | 102.4 | 0.0022158 | cluster_s7 |
| turkey | 20 | 97.2 | 0.0000000 | cluster_s7 |
| small ruminants | 636 | Inf | 0.0241480 | cluster_s7 |

**Supplementary Table 4**: Odds ratios and p-values for the differential distribution of the various genotypes in human and food *Lm* sequences; a false discovery rate correction has been used for the p-values; cluster_s7=clusters have been delineated using single-linkage hierarchical clustering with a threshold of seven.

| source | genotype | OR | pval_fdr | typing |
| --- | --- | --- | --- | --- |
| human | IIa | 0.5 | <0.0001 | serogroup |
| human | IIb | 1.5 | =0.015 | serogroup |
| human | IIc | 0.1 | <0.0001 | serogroup |
| human | IVb | 7.3 | <0.0001 | serogroup |
| human | CC1 | 4.2 | 2.01538711203685e-13 | CC |
| human | CC101 | 1.6 | 0.573891853500276 | CC |
| human | CC11 | 0.4 | 0.137150851627193 | CC |
| human | CC121 | 0.0 | 6.42128407101102e-29 | CC |
| human | CC124 | 0.0 | 1 | CC |
| human | CC14 | 2.6 | 0.00507768030985597 | CC |
| human | CC155 | 5.0 | 8.54961277789719e-06 | CC |
| human | CC177 | Inf | 0.589668541055374 | CC |
| human | CC18 | 1.3 | 0.618446063987045 | CC |
| human | CC183 | Inf | 0.589668541055374 | CC |
| human | CC19 | 3.5 | 0.039849534587575 | CC |
| human | CC193 | 0.0 | 0.460160643098533 | CC |
| human | CC199 | 0.0 | 1.19917399324362e-07 | CC |
| human | CC2 | 5.4 | 2.55609256891984e-11 | CC |
| human | CC20 | 0.8 | 1 | CC |
| human | CC200 | 0.3 | 0.244336926434731 | CC |
| human | CC204 | 0.4 | 0.0818984920524743 | CC |
| human | CC21 | 3.1 | 0.573891853500276 | CC |
| human | CC217 | Inf | 0.460160643098533 | CC |
| human | CC218 | 4.1 | 0.450048615127044 | CC |
| human | CC220 | 0.0 | 1 | CC |
| human | CC224 | 1.4 | 0.589668541055374 | CC |
| human | CC26 | 1.0 | 1 | CC |
| human | CC288 | 5.1 | 0.285757479221832 | CC |
| human | CC29 | 4.1 | 0.450048615127044 | CC |
| human | CC3 | 3.1 | 0.0514774485275776 | CC |
| human | CC31 | 0.1 | 0.00322870585602649 | CC |
| human | CC315 | Inf | 0.285757479221832 | CC |
| human | CC321 | 0.0 | 0.00507768030985597 | CC |
| human | CC37 | 0.9 | 0.894087793363637 | CC |
| human | CC379 | 11.4 | 0.013816930217023 | CC |
| human | CC388 | Inf | 0.102505245458156 | CC |
| human | CC389 | Inf | 0.285757479221832 | CC |
| human | CC392 | Inf | 0.589668541055374 | CC |
| human | CC4 | 4.9 | 0.00638711000824245 | CC |
| human | CC403 | 0.4 | 0.589668541055374 | CC |
| human | CC412 | 4.1 | 0.450048615127044 | CC |
| human | CC415 | Inf | 0.589668541055374 | CC |
| human | CC426 | 0.0 | 0.589668541055374 | CC |
| human | CC475 | 0.3 | 0.175179042143196 | CC |
| human | CC489 | 0.7 | 1 | CC |
| human | CC5 | 0.6 | 0.210874878046967 | CC |
| human | CC517 | Inf | 0.589668541055374 | CC |
| human | CC54 | 3.1 | 0.573891853500276 | CC |
| human | CC59 | 2.1 | 0.465817810066142 | CC |
| human | CC6 | 5.2 | 2.2208942231901e-15 | CC |
| human | CC7 | 0.2 | 3.58128032478066e-06 | CC |
| human | CC77 | 2.6 | 0.477647503141584 | CC |
| human | CC8 | 1.3 | 0.468560658818128 | CC |
| human | CC87 | 3.1 | 0.244336926434731 | CC |
| human | CC89 | Inf | 0.589668541055374 | CC |
| human | CC9 | 0.1 | 7.06022065185662e-34 | CC |
| human | CC90 | Inf | 0.460160643098533 | CC |
| human | ST13 | Inf | 1 | CC |
| human | ST32 | Inf | 1 | CC |
| human | ST36 | 0.0 | 0.589668541055374 | CC |
| human | ST585 | 0.0 | 0.589668541055374 | CC |
| human | ST773 | 0.0 | 0.460160643098533 | CC |
| human | ST839 | Inf | 0.589668541055374 | CC |
| human | 327 | 18.1 | 3.80882273186362e-10 | cluster_s7 |
| human | 208 | Inf | 0.00161099497806003 | cluster_s7 |
| human | 294 | Inf | 0.00161099497806003 | cluster_s7 |
| human | 145 | 0.0 | 3.22468149466277e-06 | cluster_s7 |
| human | 274 | 0.0 | 0.000168074995528159 | cluster_s7 |
| human | 20 | 0.1 | 0.00801667292380219 | cluster_s7 |
| human | 2 | 0.0 | 7.81583392820446e-14 | cluster_s7 |

**Supplementary Table 5**: Average prevalences and ranges for the various genotypes in human and food *Lm* sequences; results summarized from 10000 Dirichlet samples.

| source | genotype | average_10000sim | range_10000sim | typing |
| --- | --- | --- | --- | --- |
| food | IIa | 56.46 | 50.04-62.51 | serogroup |
| food | IIb | 8.52 | 5.23-12.27 | serogroup |
| food | IIc | 23.77 | 17.83-29.9 | serogroup |
| food | IVb | 11.11 | 7.46-16.16 | serogroup |
| human | IIa | 37.28 | 30.88-44 | serogroup |
| human | IIb | 12.36 | 8.14-18.31 | serogroup |
| human | IIc | 2.77 | 1.08-5.59 | serogroup |
| human | IVb | 47.46 | 41.05-55.25 | serogroup |
| food | CC1 | 4.12 | 2.04-7.7 | CC |
| food | CC101 | 0.97 | 0.18-2.69 | CC |
| food | CC11 | 2.29 | 0.85-5.82 | CC |
| food | CC121 | 14.63 | 10.16-20.14 | CC |
| food | CC124 | 0.24 | 0-1.91 | CC |
| food | CC14 | 2.06 | 0.72-4.14 | CC |
| food | CC155 | 1.21 | 0.3-3.29 | CC |
| food | CC177 | 0.12 | 0-1.05 | CC |
| food | CC18 | 1.33 | 0.29-3.29 | CC |
| food | CC183 | 0.12 | 0-1.06 | CC |
| food | CC19 | 0.73 | 0.03-2.38 | CC |
| food | CC193 | 0.48 | 0.03-1.99 | CC |
| food | CC199 | 3.39 | 1.46-6.31 | CC |
| food | CC2 | 2.18 | 0.72-4.8 | CC |
| food | CC20 | 0.61 | 0.05-2.14 | CC |
| food | CC200 | 1.34 | 0.39-3.46 | CC |
| food | CC204 | 3.39 | 1.43-6.47 | CC |
| food | CC21 | 0.24 | 0-1.46 | CC |
| food | CC217 | 0.12 | 0-1.05 | CC |
| food | CC218 | 0.24 | 0-1.53 | CC |
| food | CC220 | 0.24 | 0-1.57 | CC |
| food | CC224 | 1.09 | 0.19-3.08 | CC |
| food | CC26 | 0.36 | 0.01-1.79 | CC |
| food | CC288 | 0.25 | 0-1.42 | CC |
| food | CC29 | 0.24 | 0-1.48 | CC |
| food | CC3 | 0.85 | 0.11-2.57 | CC |
| food | CC31 | 1.94 | 0.57-4.93 | CC |
| food | CC315 | 0.12 | 0-1.1 | CC |
| food | CC321 | 1.45 | 0.26-3.41 | CC |
| food | CC37 | 3.76 | 1.62-6.75 | CC |
| food | CC379 | 0.24 | 0-1.43 | CC |
| food | CC388 | 0.12 | 0-1.07 | CC |
| food | CC389 | 0.12 | 0-1.17 | CC |
| food | CC392 | 0.12 | 0-1.22 | CC |
| food | CC4 | 0.61 | 0.04-2.42 | CC |
| food | CC403 | 0.73 | 0.08-2.27 | CC |
| food | CC412 | 0.24 | 0-1.66 | CC |
| food | CC415 | 0.12 | 0-1.29 | CC |
| food | CC426 | 0.36 | 0.01-2.26 | CC |
| food | CC475 | 1.45 | 0.42-3.62 | CC |
| food | CC489 | 0.49 | 0.02-2.4 | CC |
| food | CC5 | 4.24 | 2-7.35 | CC |
| food | CC517 | 0.12 | 0-1.06 | CC |
| food | CC54 | 0.24 | 0-1.62 | CC |
| food | CC59 | 0.60 | 0.04-2.13 | CC |
| food | CC6 | 3.26 | 1.39-5.82 | CC |
| food | CC7 | 6.77 | 4-10.35 | CC |
| food | CC77 | 0.36 | 0.01-1.72 | CC |
| food | CC8 | 5.94 | 3.29-9.35 | CC |
| food | CC87 | 0.49 | 0.02-1.85 | CC |
| food | CC89 | 0.12 | 0-1.29 | CC |
| food | CC9 | 21.67 | 16.79-27.68 | CC |
| food | CC90 | 0.12 | 0-1.12 | CC |
| food | ST13 | 0.24 | 0-1.34 | CC |
| food | ST32 | 0.24 | 0-1.3 | CC |
| food | ST36 | 0.12 | 0-1.18 | CC |
| food | ST585 | 0.12 | 0-1.45 | CC |
| food | ST773 | 0.12 | 0-1.29 | CC |
| food | ST839 | 0.36 | 0.01-1.73 | CC |
| human | CC1 | 14.93 | 10.31-20.22 | CC |
| human | CC101 | 1.49 | 0.42-3.51 | CC |
| human | CC11 | 0.98 | 0.17-2.71 | CC |
| human | CC121 | 0.86 | 0.1-2.71 | CC |
| human | CC124 | 0.12 | 0-1.47 | CC |
| human | CC14 | 5.05 | 2.59-9 | CC |
| human | CC155 | 5.31 | 2.86-8.84 | CC |
| human | CC177 | 0.25 | 0-1.51 | CC |
| human | CC18 | 1.73 | 0.36-4.23 | CC |
| human | CC183 | 0.25 | 0-1.56 | CC |
| human | CC19 | 2.22 | 0.8-5.04 | CC |
| human | CC193 | 0.12 | 0-1.24 | CC |
| human | CC199 | 0.12 | 0-1.5 | CC |
| human | CC2 | 10.24 | 6.53-15.48 | CC |
| human | CC20 | 0.49 | 0.03-2.18 | CC |
| human | CC200 | 0.49 | 0.03-2.09 | CC |
| human | CC204 | 1.61 | 0.48-3.79 | CC |
| human | CC21 | 0.49 | 0.02-1.85 | CC |
| human | CC217 | 0.37 | 0.01-1.85 | CC |
| human | CC218 | 0.62 | 0.06-2.43 | CC |
| human | CC220 | 0.13 | 0-1.07 | CC |
| human | CC224 | 1.48 | 0.39-3.53 | CC |
| human | CC26 | 0.37 | 0.01-2.66 | CC |
| human | CC288 | 0.74 | 0.07-2.31 | CC |
| human | CC29 | 0.61 | 0.05-2.21 | CC |
| human | CC3 | 2.34 | 0.91-4.76 | CC |
| human | CC31 | 0.25 | 0-1.65 | CC |
| human | CC315 | 0.49 | 0.01-1.73 | CC |
| human | CC321 | 0.12 | 0-1.07 | CC |
| human | CC37 | 3.46 | 1.28-6.38 | CC |
| human | CC379 | 1.47 | 0.35-4.05 | CC |
| human | CC388 | 0.74 | 0.1-2.58 | CC |
| human | CC389 | 0.49 | 0.04-2.02 | CC |
| human | CC392 | 0.24 | 0-1.56 | CC |
| human | CC4 | 2.47 | 1-5.55 | CC |
| human | CC403 | 0.37 | 0-1.71 | CC |
| human | CC412 | 0.62 | 0.06-2.15 | CC |
| human | CC415 | 0.25 | 0-1.35 | CC |
| human | CC426 | 0.13 | 0-1.15 | CC |
| human | CC475 | 0.49 | 0.02-1.75 | CC |
| human | CC489 | 0.37 | 0.01-1.78 | CC |
| human | CC5 | 2.58 | 1.03-5.24 | CC |
| human | CC517 | 0.24 | 0-1.3 | CC |
| human | CC54 | 0.49 | 0.01-1.9 | CC |
| human | CC59 | 1.11 | 0.18-3.04 | CC |
| human | CC6 | 14.41 | 9.77-18.89 | CC |
| human | CC7 | 1.85 | 0.52-4.65 | CC |
| human | CC77 | 0.74 | 0.09-2.58 | CC |
| human | CC8 | 7.27 | 4.42-10.48 | CC |
| human | CC87 | 1.24 | 0.3-3.24 | CC |
| human | CC89 | 0.25 | 0-1.33 | CC |
| human | CC9 | 2.71 | 1.04-5.04 | CC |
| human | CC90 | 0.37 | 0-1.63 | CC |
| human | ST13 | 0.12 | 0-1.04 | CC |
| human | ST32 | 0.12 | 0-1.15 | CC |
| human | ST36 | 0.25 | 0-1.67 | CC |
| human | ST585 | 0.25 | 0-1.5 | CC |
| human | ST773 | 0.37 | 0.01-1.74 | CC |
| human | ST839 | 0.12 | 0-1.52 | CC |
| food | 1 | 0.26 | 0.01-1.14 | cluster_s7 |
| food | 2 | 3.45 | 1.84-5.47 | cluster_s7 |
| food | 3 | 0.57 | 0.12-1.46 | cluster_s7 |
| food | 4 | 0.06 | 0-0.57 | cluster_s7 |
| food | 5 | 0.06 | 0-0.69 | cluster_s7 |
| food | 6 | 0.06 | 0-0.48 | cluster_s7 |
| food | 7 | 0.06 | 0-0.55 | cluster_s7 |
| food | 8 | 0.06 | 0-0.48 | cluster_s7 |
| food | 9 | 0.06 | 0-0.58 | cluster_s7 |
| food | 10 | 0.06 | 0-0.65 | cluster_s7 |
| food | 11 | 0.06 | 0-0.65 | cluster_s7 |
| food | 12 | 0.06 | 0-0.69 | cluster_s7 |
| food | 13 | 0.06 | 0-0.54 | cluster_s7 |
| food | 14 | 0.13 | 0-0.74 | cluster_s7 |
| food | 15 | 0.06 | 0-0.55 | cluster_s7 |
| food | 16 | 0.06 | 0-0.58 | cluster_s7 |
| food | 17 | 0.06 | 0-0.57 | cluster_s7 |
| food | 18 | 0.06 | 0-0.67 | cluster_s7 |
| food | 19 | 0.06 | 0-0.52 | cluster_s7 |
| food | 20 | 1.21 | 0.44-2.42 | cluster_s7 |
| food | 21 | 0.06 | 0-0.56 | cluster_s7 |
| food | 22 | 0.06 | 0-0.85 | cluster_s7 |
| food | 23 | 0.06 | 0-0.55 | cluster_s7 |
| food | 24 | 0.06 | 0-0.55 | cluster_s7 |
| food | 25 | 0.13 | 0-0.8 | cluster_s7 |
| food | 26 | 0.06 | 0-0.53 | cluster_s7 |
| food | 27 | 0.06 | 0-0.51 | cluster_s7 |
| food | 28 | 0.06 | 0-0.74 | cluster_s7 |
| food | 29 | 0.32 | 0.03-1.22 | cluster_s7 |
| food | 30 | 0.06 | 0-0.51 | cluster_s7 |
| food | 31 | 0.13 | 0-0.74 | cluster_s7 |
| food | 32 | 0.06 | 0-0.54 | cluster_s7 |
| food | 33 | 0.06 | 0-0.53 | cluster_s7 |
| food | 34 | 0.13 | 0-0.75 | cluster_s7 |
| food | 35 | 0.13 | 0-0.78 | cluster_s7 |
| food | 36 | 0.13 | 0-0.73 | cluster_s7 |
| food | 37 | 0.13 | 0-0.83 | cluster_s7 |
| food | 38 | 0.13 | 0-0.83 | cluster_s7 |
| food | 39 | 0.13 | 0-0.78 | cluster_s7 |
| food | 40 | 0.32 | 0.03-1.22 | cluster_s7 |
| food | 41 | 0.13 | 0-0.88 | cluster_s7 |
| food | 42 | 0.32 | 0.02-1.17 | cluster_s7 |
| food | 43 | 0.13 | 0-0.85 | cluster_s7 |
| food | 44 | 0.13 | 0-0.78 | cluster_s7 |
| food | 45 | 0.32 | 0.03-1.19 | cluster_s7 |
| food | 46 | 0.32 | 0.02-1.39 | cluster_s7 |
| food | 47 | 0.19 | 0-0.92 | cluster_s7 |
| food | 48 | 0.25 | 0.01-1.02 | cluster_s7 |
| food | 49 | 0.13 | 0-0.78 | cluster_s7 |
| food | 50 | 0.19 | 0-0.86 | cluster_s7 |
| food | 51 | 0.39 | 0.04-1.39 | cluster_s7 |
| food | 52 | 0.13 | 0-1.01 | cluster_s7 |
| food | 53 | 0.64 | 0.12-1.88 | cluster_s7 |
| food | 54 | 0.13 | 0-0.78 | cluster_s7 |
| food | 55 | 0.13 | 0-0.8 | cluster_s7 |
| food | 56 | 0.13 | 0-0.74 | cluster_s7 |
| food | 57 | 0.13 | 0-1.19 | cluster_s7 |
| food | 58 | 0.13 | 0-1.04 | cluster_s7 |
| food | 59 | 0.13 | 0-0.98 | cluster_s7 |
| food | 60 | 0.13 | 0-0.72 | cluster_s7 |
| food | 61 | 0.19 | 0.01-0.89 | cluster_s7 |
| food | 62 | 0.19 | 0-0.93 | cluster_s7 |
| food | 63 | 0.13 | 0-0.67 | cluster_s7 |
| food | 64 | 0.13 | 0-0.74 | cluster_s7 |
| food | 65 | 0.13 | 0-0.68 | cluster_s7 |
| food | 66 | 0.19 | 0-1.07 | cluster_s7 |
| food | 67 | 0.26 | 0.02-0.92 | cluster_s7 |
| food | 68 | 0.19 | 0-0.8 | cluster_s7 |
| food | 69 | 0.13 | 0-0.72 | cluster_s7 |
| food | 70 | 0.19 | 0-1.07 | cluster_s7 |
| food | 71 | 0.45 | 0.06-1.38 | cluster_s7 |
| food | 72 | 0.25 | 0.01-0.94 | cluster_s7 |
| food | 73 | 0.13 | 0-0.91 | cluster_s7 |
| food | 74 | 0.51 | 0.09-1.76 | cluster_s7 |
| food | 75 | 0.13 | 0-0.78 | cluster_s7 |
| food | 76 | 0.13 | 0-0.71 | cluster_s7 |
| food | 77 | 0.13 | 0-0.74 | cluster_s7 |
| food | 78 | 0.13 | 0-0.7 | cluster_s7 |
| food | 79 | 0.13 | 0-0.78 | cluster_s7 |
| food | 80 | 0.13 | 0-0.69 | cluster_s7 |
| food | 81 | 0.45 | 0.06-1.41 | cluster_s7 |
| food | 82 | 0.19 | 0-1.19 | cluster_s7 |
| food | 83 | 0.32 | 0.02-1.09 | cluster_s7 |
| food | 84 | 0.13 | 0-0.84 | cluster_s7 |
| food | 85 | 0.19 | 0-1.07 | cluster_s7 |
| food | 86 | 0.32 | 0.03-1.38 | cluster_s7 |
| food | 87 | 0.13 | 0-0.87 | cluster_s7 |
| food | 88 | 0.13 | 0-0.9 | cluster_s7 |
| food | 89 | 0.13 | 0-0.96 | cluster_s7 |
| food | 90 | 0.38 | 0.04-1.25 | cluster_s7 |
| food | 91 | 0.13 | 0-0.89 | cluster_s7 |
| food | 92 | 0.13 | 0-0.74 | cluster_s7 |
| food | 93 | 0.13 | 0-0.82 | cluster_s7 |
| food | 94 | 0.13 | 0-0.83 | cluster_s7 |
| food | 95 | 0.13 | 0-0.63 | cluster_s7 |
| food | 96 | 0.19 | 0-1.05 | cluster_s7 |
| food | 97 | 0.19 | 0-0.88 | cluster_s7 |
| food | 98 | 0.13 | 0-0.76 | cluster_s7 |
| food | 99 | 0.13 | 0-0.74 | cluster_s7 |
| food | 100 | 0.26 | 0.01-1.04 | cluster_s7 |
| food | 101 | 0.13 | 0-0.73 | cluster_s7 |
| food | 102 | 0.06 | 0-0.74 | cluster_s7 |
| food | 103 | 0.06 | 0-0.52 | cluster_s7 |
| food | 104 | 0.06 | 0-0.65 | cluster_s7 |
| food | 105 | 0.06 | 0-1 | cluster_s7 |
| food | 106 | 0.06 | 0-0.55 | cluster_s7 |
| food | 107 | 0.38 | 0.04-1.22 | cluster_s7 |
| food | 108 | 0.06 | 0-0.63 | cluster_s7 |
| food | 109 | 0.06 | 0-0.52 | cluster_s7 |
| food | 110 | 0.13 | 0-0.78 | cluster_s7 |
| food | 111 | 0.06 | 0-0.58 | cluster_s7 |
| food | 112 | 0.96 | 0.29-2.21 | cluster_s7 |
| food | 113 | 0.06 | 0-0.67 | cluster_s7 |
| food | 114 | 0.06 | 0-0.59 | cluster_s7 |
| food | 115 | 0.06 | 0-0.74 | cluster_s7 |
| food | 116 | 0.06 | 0-0.66 | cluster_s7 |
| food | 117 | 0.06 | 0-0.51 | cluster_s7 |
| food | 118 | 0.07 | 0-0.62 | cluster_s7 |
| food | 119 | 0.06 | 0-0.58 | cluster_s7 |
| food | 120 | 0.06 | 0-0.64 | cluster_s7 |
| food | 121 | 0.06 | 0-0.57 | cluster_s7 |
| food | 122 | 0.06 | 0-0.66 | cluster_s7 |
| food | 123 | 0.06 | 0-0.6 | cluster_s7 |
| food | 124 | 0.06 | 0-0.54 | cluster_s7 |
| food | 125 | 0.06 | 0-0.5 | cluster_s7 |
| food | 126 | 0.06 | 0-0.64 | cluster_s7 |
| food | 127 | 0.06 | 0-0.56 | cluster_s7 |
| food | 128 | 0.06 | 0-0.59 | cluster_s7 |
| food | 129 | 0.06 | 0-0.58 | cluster_s7 |
| food | 130 | 0.06 | 0-0.64 | cluster_s7 |
| food | 131 | 0.06 | 0-0.8 | cluster_s7 |
| food | 132 | 0.06 | 0-0.6 | cluster_s7 |
| food | 133 | 0.06 | 0-0.67 | cluster_s7 |
| food | 134 | 0.07 | 0-0.6 | cluster_s7 |
| food | 135 | 0.13 | 0-0.78 | cluster_s7 |
| food | 136 | 0.06 | 0-0.56 | cluster_s7 |
| food | 137 | 0.06 | 0-0.63 | cluster_s7 |
| food | 138 | 0.38 | 0.06-1.2 | cluster_s7 |
| food | 139 | 0.64 | 0.16-1.61 | cluster_s7 |
| food | 140 | 0.19 | 0-0.92 | cluster_s7 |
| food | 141 | 0.13 | 0-0.82 | cluster_s7 |
| food | 142 | 0.13 | 0-0.83 | cluster_s7 |
| food | 143 | 0.13 | 0-0.87 | cluster_s7 |
| food | 144 | 0.13 | 0-0.83 | cluster_s7 |
| food | 145 | 1.79 | 0.56-3.51 | cluster_s7 |
| food | 146 | 0.13 | 0-0.75 | cluster_s7 |
| food | 147 | 0.32 | 0.03-1.08 | cluster_s7 |
| food | 148 | 0.19 | 0-0.93 | cluster_s7 |
| food | 149 | 0.19 | 0.01-0.94 | cluster_s7 |
| food | 150 | 0.19 | 0.01-0.93 | cluster_s7 |
| food | 151 | 0.13 | 0-0.75 | cluster_s7 |
| food | 152 | 0.13 | 0-0.82 | cluster_s7 |
| food | 153 | 0.13 | 0-0.72 | cluster_s7 |
| food | 154 | 0.13 | 0-0.68 | cluster_s7 |
| food | 155 | 0.13 | 0-0.7 | cluster_s7 |
| food | 156 | 0.39 | 0.03-1.18 | cluster_s7 |
| food | 157 | 0.13 | 0-0.8 | cluster_s7 |
| food | 158 | 0.38 | 0.05-1.25 | cluster_s7 |
| food | 159 | 0.26 | 0.01-1.05 | cluster_s7 |
| food | 160 | 0.13 | 0-0.7 | cluster_s7 |
| food | 161 | 0.13 | 0-0.85 | cluster_s7 |
| food | 162 | 0.13 | 0-0.71 | cluster_s7 |
| food | 163 | 0.19 | 0.01-1.05 | cluster_s7 |
| food | 164 | 0.13 | 0-0.73 | cluster_s7 |
| food | 165 | 0.13 | 0-0.86 | cluster_s7 |
| food | 166 | 0.13 | 0-0.83 | cluster_s7 |
| food | 167 | 0.13 | 0-0.78 | cluster_s7 |
| food | 168 | 0.19 | 0-0.89 | cluster_s7 |
| food | 169 | 0.13 | 0-0.87 | cluster_s7 |
| food | 170 | 0.13 | 0-0.87 | cluster_s7 |
| food | 171 | 0.13 | 0-0.94 | cluster_s7 |
| food | 172 | 0.19 | 0-0.91 | cluster_s7 |
| food | 173 | 0.38 | 0.04-1.24 | cluster_s7 |
| food | 174 | 0.19 | 0.01-0.87 | cluster_s7 |
| food | 175 | 0.13 | 0-1.03 | cluster_s7 |
| food | 176 | 0.13 | 0-0.68 | cluster_s7 |
| food | 177 | 0.13 | 0-0.8 | cluster_s7 |
| food | 178 | 0.26 | 0.02-1 | cluster_s7 |
| food | 179 | 0.19 | 0-0.79 | cluster_s7 |
| food | 180 | 0.13 | 0-0.69 | cluster_s7 |
| food | 181 | 0.13 | 0-0.7 | cluster_s7 |
| food | 182 | 0.13 | 0-0.74 | cluster_s7 |
| food | 183 | 0.13 | 0-0.75 | cluster_s7 |
| food | 184 | 0.32 | 0.02-1.32 | cluster_s7 |
| food | 185 | 0.32 | 0.03-1.13 | cluster_s7 |
| food | 186 | 0.13 | 0-0.91 | cluster_s7 |
| food | 187 | 0.13 | 0-0.79 | cluster_s7 |
| food | 188 | 0.13 | 0-0.85 | cluster_s7 |
| food | 189 | 0.13 | 0-0.72 | cluster_s7 |
| food | 190 | 0.26 | 0.01-1.03 | cluster_s7 |
| food | 191 | 0.13 | 0-0.83 | cluster_s7 |
| food | 192 | 0.13 | 0-0.76 | cluster_s7 |
| food | 193 | 0.13 | 0-1.07 | cluster_s7 |
| food | 194 | 0.13 | 0-0.8 | cluster_s7 |
| food | 195 | 0.06 | 0-0.58 | cluster_s7 |
| food | 196 | 0.06 | 0-0.67 | cluster_s7 |
| food | 197 | 0.06 | 0-0.64 | cluster_s7 |
| food | 198 | 0.06 | 0-0.64 | cluster_s7 |
| food | 199 | 0.06 | 0-0.54 | cluster_s7 |
| food | 200 | 0.06 | 0-0.64 | cluster_s7 |
| food | 201 | 0.06 | 0-0.6 | cluster_s7 |
| food | 202 | 0.06 | 0-0.58 | cluster_s7 |
| food | 203 | 0.06 | 0-0.6 | cluster_s7 |
| food | 204 | 0.06 | 0-0.59 | cluster_s7 |
| food | 205 | 0.06 | 0-0.69 | cluster_s7 |
| food | 206 | 0.06 | 0-0.56 | cluster_s7 |
| food | 207 | 0.06 | 0-0.63 | cluster_s7 |
| food | 208 | 0.06 | 0-0.66 | cluster_s7 |
| food | 209 | 0.06 | 0-0.59 | cluster_s7 |
| food | 210 | 0.06 | 0-0.5 | cluster_s7 |
| food | 211 | 0.07 | 0-0.7 | cluster_s7 |
| food | 212 | 0.06 | 0-0.64 | cluster_s7 |
| food | 213 | 1.28 | 0.51-2.67 | cluster_s7 |
| food | 214 | 0.06 | 0-0.63 | cluster_s7 |
| food | 215 | 0.25 | 0.02-1.02 | cluster_s7 |
| food | 216 | 0.06 | 0-0.64 | cluster_s7 |
| food | 217 | 0.06 | 0-0.58 | cluster_s7 |
| food | 218 | 0.38 | 0.05-1.14 | cluster_s7 |
| food | 219 | 0.13 | 0-0.81 | cluster_s7 |
| food | 220 | 0.13 | 0-0.65 | cluster_s7 |
| food | 221 | 0.13 | 0-0.8 | cluster_s7 |
| food | 222 | 0.45 | 0.05-1.47 | cluster_s7 |
| food | 223 | 0.13 | 0-0.76 | cluster_s7 |
| food | 224 | 0.13 | 0-0.89 | cluster_s7 |
| food | 225 | 0.13 | 0-0.7 | cluster_s7 |
| food | 226 | 0.13 | 0-0.74 | cluster_s7 |
| food | 227 | 0.13 | 0-0.83 | cluster_s7 |
| food | 228 | 0.19 | 0.01-0.93 | cluster_s7 |
| food | 229 | 0.19 | 0-0.86 | cluster_s7 |
| food | 230 | 0.13 | 0-0.8 | cluster_s7 |
| food | 231 | 0.13 | 0-0.84 | cluster_s7 |
| food | 232 | 0.19 | 0-0.94 | cluster_s7 |
| food | 233 | 0.13 | 0-0.72 | cluster_s7 |
| food | 234 | 0.13 | 0-0.93 | cluster_s7 |
| food | 235 | 0.13 | 0-0.86 | cluster_s7 |
| food | 236 | 0.19 | 0.01-0.84 | cluster_s7 |
| food | 237 | 0.13 | 0-0.85 | cluster_s7 |
| food | 238 | 0.13 | 0-0.73 | cluster_s7 |
| food | 239 | 0.26 | 0.01-0.96 | cluster_s7 |
| food | 240 | 0.13 | 0-0.75 | cluster_s7 |
| food | 241 | 0.13 | 0-0.79 | cluster_s7 |
| food | 242 | 0.13 | 0-0.86 | cluster_s7 |
| food | 243 | 0.13 | 0-0.69 | cluster_s7 |
| food | 244 | 0.13 | 0-0.8 | cluster_s7 |
| food | 245 | 0.13 | 0-0.94 | cluster_s7 |
| food | 246 | 0.13 | 0-0.81 | cluster_s7 |
| food | 247 | 0.13 | 0-0.83 | cluster_s7 |
| food | 248 | 0.13 | 0-0.9 | cluster_s7 |
| food | 249 | 0.13 | 0-0.77 | cluster_s7 |
| food | 250 | 0.06 | 0-0.54 | cluster_s7 |
| food | 251 | 0.06 | 0-0.66 | cluster_s7 |
| food | 252 | 0.06 | 0-0.67 | cluster_s7 |
| food | 253 | 0.32 | 0.03-1.05 | cluster_s7 |
| food | 254 | 0.06 | 0-0.6 | cluster_s7 |
| food | 255 | 0.06 | 0-0.69 | cluster_s7 |
| food | 256 | 0.06 | 0-0.65 | cluster_s7 |
| food | 257 | 0.06 | 0-0.66 | cluster_s7 |
| food | 258 | 0.06 | 0-0.54 | cluster_s7 |
| food | 259 | 0.06 | 0-0.6 | cluster_s7 |
| food | 260 | 0.06 | 0-0.66 | cluster_s7 |
| food | 261 | 0.06 | 0-0.54 | cluster_s7 |
| food | 262 | 0.06 | 0-0.62 | cluster_s7 |
| food | 263 | 0.06 | 0-0.59 | cluster_s7 |
| food | 264 | 0.13 | 0-0.82 | cluster_s7 |
| food | 265 | 0.06 | 0-0.65 | cluster_s7 |
| food | 266 | 0.06 | 0-0.72 | cluster_s7 |
| food | 267 | 0.06 | 0-0.61 | cluster_s7 |
| food | 268 | 0.06 | 0-0.59 | cluster_s7 |
| food | 269 | 0.06 | 0-0.57 | cluster_s7 |
| food | 270 | 0.13 | 0-0.92 | cluster_s7 |
| food | 271 | 0.06 | 0-0.75 | cluster_s7 |
| food | 272 | 0.13 | 0-0.8 | cluster_s7 |
| food | 273 | 0.13 | 0-0.69 | cluster_s7 |
| food | 274 | 1.40 | 0.41-2.75 | cluster_s7 |
| food | 275 | 0.13 | 0-0.76 | cluster_s7 |
| food | 276 | 0.13 | 0-0.72 | cluster_s7 |
| food | 277 | 0.13 | 0-0.82 | cluster_s7 |
| food | 278 | 0.13 | 0-0.75 | cluster_s7 |
| food | 279 | 0.13 | 0-0.8 | cluster_s7 |
| food | 280 | 0.13 | 0-0.96 | cluster_s7 |
| food | 281 | 0.13 | 0-0.87 | cluster_s7 |
| food | 282 | 0.13 | 0-0.76 | cluster_s7 |
| food | 283 | 0.13 | 0-0.79 | cluster_s7 |
| food | 284 | 0.13 | 0-0.7 | cluster_s7 |
| food | 285 | 0.13 | 0-0.82 | cluster_s7 |
| food | 286 | 0.13 | 0-0.78 | cluster_s7 |
| food | 287 | 0.13 | 0-0.81 | cluster_s7 |
| food | 288 | 0.19 | 0-0.85 | cluster_s7 |
| food | 289 | 0.13 | 0-0.83 | cluster_s7 |
| food | 290 | 0.19 | 0-0.88 | cluster_s7 |
| food | 291 | 0.06 | 0-0.6 | cluster_s7 |
| food | 292 | 0.06 | 0-0.71 | cluster_s7 |
| food | 293 | 0.45 | 0.08-1.2 | cluster_s7 |
| food | 294 | 0.06 | 0-0.64 | cluster_s7 |
| food | 295 | 0.06 | 0-0.72 | cluster_s7 |
| food | 296 | 0.06 | 0-0.64 | cluster_s7 |
| food | 297 | 0.06 | 0-0.6 | cluster_s7 |
| food | 298 | 0.06 | 0-0.56 | cluster_s7 |
| food | 299 | 0.06 | 0-0.63 | cluster_s7 |
| food | 300 | 0.06 | 0-0.58 | cluster_s7 |
| food | 301 | 0.06 | 0-0.57 | cluster_s7 |
| food | 302 | 0.06 | 0-0.62 | cluster_s7 |
| food | 303 | 0.06 | 0-0.63 | cluster_s7 |
| food | 304 | 0.13 | 0-1.01 | cluster_s7 |
| food | 305 | 0.13 | 0-0.65 | cluster_s7 |
| food | 306 | 0.32 | 0.03-1.13 | cluster_s7 |
| food | 307 | 0.26 | 0.01-0.98 | cluster_s7 |
| food | 308 | 0.19 | 0-0.79 | cluster_s7 |
| food | 309 | 0.19 | 0-0.87 | cluster_s7 |
| food | 310 | 0.13 | 0-0.72 | cluster_s7 |
| food | 311 | 0.13 | 0-0.81 | cluster_s7 |
| food | 312 | 0.13 | 0-0.7 | cluster_s7 |
| food | 313 | 0.13 | 0-0.83 | cluster_s7 |
| food | 314 | 0.13 | 0-0.81 | cluster_s7 |
| food | 315 | 0.13 | 0-0.71 | cluster_s7 |
| food | 316 | 0.13 | 0-0.79 | cluster_s7 |
| food | 317 | 0.25 | 0.01-1.03 | cluster_s7 |
| food | 318 | 0.19 | 0.01-0.82 | cluster_s7 |
| food | 319 | 0.13 | 0-1.02 | cluster_s7 |
| food | 320 | 0.52 | 0.04-1.73 | cluster_s7 |
| food | 321 | 0.13 | 0-0.77 | cluster_s7 |
| food | 322 | 0.13 | 0-0.67 | cluster_s7 |
| food | 323 | 0.13 | 0-0.85 | cluster_s7 |
| food | 324 | 0.25 | 0.01-1.12 | cluster_s7 |
| food | 325 | 0.13 | 0-0.7 | cluster_s7 |
| food | 326 | 0.13 | 0-0.8 | cluster_s7 |
| food | 327 | 0.26 | 0.01-1.02 | cluster_s7 |
| food | 328 | 0.06 | 0-0.58 | cluster_s7 |
| food | 329 | 0.06 | 0-0.66 | cluster_s7 |
| food | 330 | 0.06 | 0-0.73 | cluster_s7 |
| food | 331 | 0.06 | 0-0.62 | cluster_s7 |
| food | 332 | 0.06 | 0-0.69 | cluster_s7 |
| food | 333 | 0.06 | 0-0.63 | cluster_s7 |
| food | 334 | 0.06 | 0-0.56 | cluster_s7 |
| food | 335 | 0.06 | 0-0.67 | cluster_s7 |
| food | 336 | 0.19 | 0-0.94 | cluster_s7 |
| food | 337 | 0.06 | 0-0.5 | cluster_s7 |
| food | 338 | 0.06 | 0-0.66 | cluster_s7 |
| food | 339 | 0.06 | 0-0.58 | cluster_s7 |
| food | 340 | 0.06 | 0-0.62 | cluster_s7 |
| food | 341 | 0.07 | 0-0.61 | cluster_s7 |
| food | 342 | 0.13 | 0-0.75 | cluster_s7 |
| food | 343 | 0.06 | 0-0.69 | cluster_s7 |
| food | 344 | 0.07 | 0-0.67 | cluster_s7 |
| food | 345 | 0.06 | 0-0.63 | cluster_s7 |
| food | 346 | 0.06 | 0-0.68 | cluster_s7 |
| food | 347 | 0.06 | 0-0.61 | cluster_s7 |
| food | 348 | 0.06 | 0-0.53 | cluster_s7 |
| food | 349 | 0.06 | 0-0.52 | cluster_s7 |
| food | 350 | 0.13 | 0-0.7 | cluster_s7 |
| food | 351 | 0.06 | 0-0.72 | cluster_s7 |
| food | 352 | 0.06 | 0-0.57 | cluster_s7 |
| food | 353 | 0.06 | 0-0.68 | cluster_s7 |
| food | 354 | 0.06 | 0-0.62 | cluster_s7 |
| food | 355 | 0.13 | 0-0.71 | cluster_s7 |
| food | 356 | 0.06 | 0-0.77 | cluster_s7 |
| food | 357 | 0.06 | 0-0.62 | cluster_s7 |
| food | 358 | 0.06 | 0-0.62 | cluster_s7 |
| food | 359 | 0.13 | 0-1.04 | cluster_s7 |
| food | 360 | 0.06 | 0-0.57 | cluster_s7 |
| food | 361 | 0.06 | 0-0.65 | cluster_s7 |
| food | 362 | 0.06 | 0-0.63 | cluster_s7 |
| food | 363 | 0.06 | 0-0.58 | cluster_s7 |
| food | 364 | 0.07 | 0-0.62 | cluster_s7 |
| food | 365 | 0.06 | 0-0.73 | cluster_s7 |
| food | 366 | 0.06 | 0-0.57 | cluster_s7 |
| food | 367 | 0.06 | 0-0.6 | cluster_s7 |
| food | 368 | 0.06 | 0-0.59 | cluster_s7 |
| food | 369 | 0.06 | 0-0.53 | cluster_s7 |
| food | 370 | 0.06 | 0-0.54 | cluster_s7 |
| food | 371 | 0.06 | 0-0.63 | cluster_s7 |
| food | 372 | 0.13 | 0-0.81 | cluster_s7 |
| food | 373 | 0.32 | 0.03-1.03 | cluster_s7 |
| food | 374 | 0.26 | 0.01-0.97 | cluster_s7 |
| food | 375 | 0.13 | 0-0.68 | cluster_s7 |
| food | 376 | 0.13 | 0-0.72 | cluster_s7 |
| food | 377 | 0.32 | 0.03-1.08 | cluster_s7 |
| food | 378 | 0.13 | 0-0.72 | cluster_s7 |
| food | 379 | 0.13 | 0-0.76 | cluster_s7 |
| food | 380 | 0.19 | 0-0.78 | cluster_s7 |
| food | 381 | 0.19 | 0.01-0.96 | cluster_s7 |
| food | 382 | 0.25 | 0.02-1.04 | cluster_s7 |
| food | 383 | 0.13 | 0-0.8 | cluster_s7 |
| food | 384 | 0.13 | 0-0.75 | cluster_s7 |
| food | 385 | 0.13 | 0-0.73 | cluster_s7 |
| food | 386 | 0.13 | 0-0.71 | cluster_s7 |
| food | 387 | 0.13 | 0-0.75 | cluster_s7 |
| food | 388 | 0.13 | 0-0.67 | cluster_s7 |
| food | 389 | 0.06 | 0-0.67 | cluster_s7 |
| food | 390 | 0.06 | 0-0.63 | cluster_s7 |
| food | 391 | 0.06 | 0-0.64 | cluster_s7 |
| food | 392 | 0.06 | 0-0.56 | cluster_s7 |
| food | 393 | 0.06 | 0-0.52 | cluster_s7 |
| food | 394 | 0.06 | 0-0.6 | cluster_s7 |
| food | 395 | 0.06 | 0-0.51 | cluster_s7 |
| food | 396 | 0.06 | 0-0.52 | cluster_s7 |
| food | 397 | 0.06 | 0-0.61 | cluster_s7 |
| food | 398 | 0.06 | 0-0.65 | cluster_s7 |
| food | 399 | 0.06 | 0-0.65 | cluster_s7 |
| food | 400 | 0.06 | 0-0.59 | cluster_s7 |
| food | 401 | 0.06 | 0-0.73 | cluster_s7 |
| food | 402 | 0.06 | 0-0.77 | cluster_s7 |
| food | 403 | 0.06 | 0-0.64 | cluster_s7 |
| food | 404 | 0.06 | 0-0.72 | cluster_s7 |
| food | 405 | 0.06 | 0-0.55 | cluster_s7 |
| food | 406 | 0.06 | 0-0.63 | cluster_s7 |
| food | 407 | 0.06 | 0-0.52 | cluster_s7 |
| food | 408 | 0.06 | 0-0.55 | cluster_s7 |
| food | 409 | 0.06 | 0-0.59 | cluster_s7 |
| food | 410 | 0.06 | 0-0.7 | cluster_s7 |
| food | 411 | 0.07 | 0-0.58 | cluster_s7 |
| food | 412 | 0.06 | 0-0.61 | cluster_s7 |
| food | 413 | 0.06 | 0-0.72 | cluster_s7 |
| food | 414 | 0.06 | 0-0.62 | cluster_s7 |
| food | 415 | 0.06 | 0-0.89 | cluster_s7 |
| food | 416 | 0.45 | 0.04-1.41 | cluster_s7 |
| food | 417 | 0.07 | 0-0.58 | cluster_s7 |
| food | 418 | 0.06 | 0-0.61 | cluster_s7 |
| food | 419 | 0.06 | 0-0.63 | cluster_s7 |
| food | 420 | 0.06 | 0-0.54 | cluster_s7 |
| food | 421 | 0.06 | 0-0.87 | cluster_s7 |
| food | 422 | 0.06 | 0-0.72 | cluster_s7 |
| food | 423 | 0.06 | 0-0.57 | cluster_s7 |
| food | 424 | 0.06 | 0-0.57 | cluster_s7 |
| food | 425 | 0.13 | 0-0.75 | cluster_s7 |
| food | 426 | 0.06 | 0-0.69 | cluster_s7 |
| food | 427 | 0.06 | 0-0.78 | cluster_s7 |
| food | 428 | 0.06 | 0-0.62 | cluster_s7 |
| food | 429 | 0.06 | 0-0.56 | cluster_s7 |
| food | 430 | 0.06 | 0-0.58 | cluster_s7 |
| food | 431 | 0.06 | 0-0.71 | cluster_s7 |
| food | 432 | 0.06 | 0-0.66 | cluster_s7 |
| food | 433 | 0.13 | 0-0.74 | cluster_s7 |
| food | 434 | 0.13 | 0-0.82 | cluster_s7 |
| food | 435 | 0.13 | 0-0.81 | cluster_s7 |
| food | 436 | 0.13 | 0-0.7 | cluster_s7 |
| food | 437 | 0.13 | 0-0.75 | cluster_s7 |
| food | 438 | 0.13 | 0-0.73 | cluster_s7 |
| food | 439 | 0.13 | 0-0.71 | cluster_s7 |
| food | 440 | 0.13 | 0-0.68 | cluster_s7 |
| food | 441 | 0.13 | 0-0.71 | cluster_s7 |
| food | 442 | 0.13 | 0-0.86 | cluster_s7 |
| food | 443 | 0.13 | 0-1.02 | cluster_s7 |
| food | 444 | 0.13 | 0-0.63 | cluster_s7 |
| food | 445 | 0.13 | 0-0.76 | cluster_s7 |
| food | 446 | 0.13 | 0-0.77 | cluster_s7 |
| food | 447 | 0.13 | 0-0.81 | cluster_s7 |
| food | 448 | 0.19 | 0-0.89 | cluster_s7 |
| food | 449 | 0.13 | 0-0.74 | cluster_s7 |
| food | 450 | 0.13 | 0-0.65 | cluster_s7 |
| food | 451 | 0.06 | 0-0.7 | cluster_s7 |
| food | 452 | 0.06 | 0-0.58 | cluster_s7 |
| food | 453 | 0.06 | 0-0.71 | cluster_s7 |
| food | 454 | 0.06 | 0-0.57 | cluster_s7 |
| food | 455 | 0.06 | 0-0.52 | cluster_s7 |
| food | 456 | 0.06 | 0-0.54 | cluster_s7 |
| food | 457 | 0.06 | 0-0.6 | cluster_s7 |
| food | 458 | 0.06 | 0-0.63 | cluster_s7 |
| food | 459 | 0.06 | 0-0.6 | cluster_s7 |
| food | 460 | 0.06 | 0-0.52 | cluster_s7 |
| food | 461 | 0.06 | 0-0.55 | cluster_s7 |
| food | 462 | 0.06 | 0-0.58 | cluster_s7 |
| food | 463 | 0.06 | 0-0.58 | cluster_s7 |
| food | 464 | 0.06 | 0-0.61 | cluster_s7 |
| food | 465 | 0.06 | 0-0.75 | cluster_s7 |
| food | 466 | 0.06 | 0-0.54 | cluster_s7 |
| food | 467 | 0.06 | 0-0.61 | cluster_s7 |
| food | 468 | 0.06 | 0-0.75 | cluster_s7 |
| food | 469 | 0.06 | 0-0.65 | cluster_s7 |
| food | 470 | 0.06 | 0-0.71 | cluster_s7 |
| food | 471 | 0.06 | 0-0.61 | cluster_s7 |
| food | 472 | 0.06 | 0-0.62 | cluster_s7 |
| food | 473 | 0.06 | 0-0.62 | cluster_s7 |
| food | 474 | 0.06 | 0-0.65 | cluster_s7 |
| food | 475 | 0.06 | 0-0.59 | cluster_s7 |
| food | 476 | 0.06 | 0-0.63 | cluster_s7 |
| food | 477 | 0.06 | 0-0.61 | cluster_s7 |
| food | 478 | 0.06 | 0-0.61 | cluster_s7 |
| food | 479 | 0.06 | 0-0.67 | cluster_s7 |
| food | 480 | 0.06 | 0-0.56 | cluster_s7 |
| food | 481 | 0.06 | 0-0.51 | cluster_s7 |
| food | 482 | 0.06 | 0-0.74 | cluster_s7 |
| food | 483 | 0.06 | 0-0.66 | cluster_s7 |
| food | 484 | 0.06 | 0-0.52 | cluster_s7 |
| food | 485 | 0.06 | 0-0.56 | cluster_s7 |
| food | 486 | 0.06 | 0-0.58 | cluster_s7 |
| food | 487 | 0.06 | 0-0.82 | cluster_s7 |
| food | 488 | 0.06 | 0-0.53 | cluster_s7 |
| food | 489 | 0.06 | 0-0.66 | cluster_s7 |
| food | 490 | 0.06 | 0-0.7 | cluster_s7 |
| food | 491 | 0.06 | 0-0.59 | cluster_s7 |
| food | 492 | 0.06 | 0-0.53 | cluster_s7 |
| food | 493 | 0.06 | 0-0.48 | cluster_s7 |
| food | 494 | 0.06 | 0-0.66 | cluster_s7 |
| food | 495 | 0.06 | 0-0.58 | cluster_s7 |
| food | 496 | 0.06 | 0-0.59 | cluster_s7 |
| food | 497 | 0.06 | 0-0.64 | cluster_s7 |
| food | 498 | 0.06 | 0-0.57 | cluster_s7 |
| food | 499 | 0.06 | 0-0.62 | cluster_s7 |
| food | 500 | 0.06 | 0-0.61 | cluster_s7 |
| food | 501 | 0.06 | 0-0.51 | cluster_s7 |
| food | 502 | 0.06 | 0-0.69 | cluster_s7 |
| food | 503 | 0.07 | 0-0.67 | cluster_s7 |
| food | 504 | 0.06 | 0-0.64 | cluster_s7 |
| food | 505 | 0.06 | 0-0.72 | cluster_s7 |
| food | 506 | 0.06 | 0-0.61 | cluster_s7 |
| food | 507 | 0.06 | 0-0.69 | cluster_s7 |
| food | 508 | 0.06 | 0-0.61 | cluster_s7 |
| food | 509 | 0.06 | 0-0.62 | cluster_s7 |
| food | 510 | 0.07 | 0-0.58 | cluster_s7 |
| food | 511 | 0.06 | 0-0.6 | cluster_s7 |
| food | 512 | 0.06 | 0-0.51 | cluster_s7 |
| food | 513 | 0.06 | 0-0.53 | cluster_s7 |
| food | 514 | 0.06 | 0-0.72 | cluster_s7 |
| food | 515 | 0.13 | 0-0.9 | cluster_s7 |
| food | 516 | 0.38 | 0.05-1.24 | cluster_s7 |
| food | 517 | 0.13 | 0-0.83 | cluster_s7 |
| food | 518 | 0.13 | 0-0.85 | cluster_s7 |
| food | 519 | 0.13 | 0-0.84 | cluster_s7 |
| food | 520 | 0.13 | 0-0.69 | cluster_s7 |
| food | 521 | 0.13 | 0-0.71 | cluster_s7 |
| food | 522 | 0.13 | 0-0.74 | cluster_s7 |
| food | 523 | 0.13 | 0-0.63 | cluster_s7 |
| food | 524 | 0.13 | 0-0.74 | cluster_s7 |
| food | 525 | 0.13 | 0-1.12 | cluster_s7 |
| food | 526 | 0.32 | 0.01-1.32 | cluster_s7 |
| food | 527 | 0.19 | 0-0.82 | cluster_s7 |
| food | 528 | 0.19 | 0-0.77 | cluster_s7 |
| food | 529 | 0.13 | 0-0.82 | cluster_s7 |
| food | 530 | 0.77 | 0.23-1.88 | cluster_s7 |
| food | 531 | 0.13 | 0-0.82 | cluster_s7 |
| food | 532 | 0.13 | 0-0.79 | cluster_s7 |
| food | 533 | 0.19 | 0-0.87 | cluster_s7 |
| food | 534 | 0.13 | 0-0.71 | cluster_s7 |
| food | 535 | 0.13 | 0-0.79 | cluster_s7 |
| food | 536 | 0.13 | 0-0.76 | cluster_s7 |
| food | 537 | 0.13 | 0-0.75 | cluster_s7 |
| food | 538 | 0.13 | 0-0.75 | cluster_s7 |
| food | 539 | 0.13 | 0-0.98 | cluster_s7 |
| food | 540 | 0.13 | 0-0.78 | cluster_s7 |
| food | 541 | 0.13 | 0-0.8 | cluster_s7 |
| food | 542 | 0.19 | 0-1.09 | cluster_s7 |
| food | 543 | 0.13 | 0-0.75 | cluster_s7 |
| food | 544 | 0.06 | 0-0.62 | cluster_s7 |
| food | 545 | 0.07 | 0-0.6 | cluster_s7 |
| food | 546 | 0.06 | 0-0.76 | cluster_s7 |
| food | 547 | 0.06 | 0-0.58 | cluster_s7 |
| food | 548 | 0.06 | 0-0.72 | cluster_s7 |
| food | 549 | 0.06 | 0-0.63 | cluster_s7 |
| food | 550 | 0.06 | 0-0.64 | cluster_s7 |
| food | 551 | 0.06 | 0-0.57 | cluster_s7 |
| food | 552 | 0.06 | 0-0.59 | cluster_s7 |
| food | 553 | 0.06 | 0-0.5 | cluster_s7 |
| food | 554 | 0.06 | 0-0.63 | cluster_s7 |
| food | 555 | 0.06 | 0-0.69 | cluster_s7 |
| food | 556 | 0.06 | 0-0.68 | cluster_s7 |
| food | 557 | 0.06 | 0-0.6 | cluster_s7 |
| food | 558 | 0.06 | 0-0.55 | cluster_s7 |
| food | 559 | 0.06 | 0-0.57 | cluster_s7 |
| food | 560 | 0.06 | 0-0.61 | cluster_s7 |
| food | 561 | 0.06 | 0-0.7 | cluster_s7 |
| food | 562 | 0.06 | 0-0.64 | cluster_s7 |
| food | 563 | 0.06 | 0-0.67 | cluster_s7 |
| food | 564 | 0.06 | 0-0.58 | cluster_s7 |
| food | 565 | 0.06 | 0-0.54 | cluster_s7 |
| food | 566 | 0.06 | 0-0.5 | cluster_s7 |
| food | 567 | 0.06 | 0-0.68 | cluster_s7 |
| food | 568 | 0.06 | 0-0.76 | cluster_s7 |
| food | 569 | 0.06 | 0-0.75 | cluster_s7 |
| food | 570 | 0.06 | 0-0.66 | cluster_s7 |
| food | 571 | 0.06 | 0-0.51 | cluster_s7 |
| food | 572 | 0.06 | 0-0.68 | cluster_s7 |
| food | 573 | 0.06 | 0-0.74 | cluster_s7 |
| food | 574 | 0.06 | 0-0.61 | cluster_s7 |
| food | 575 | 0.06 | 0-0.5 | cluster_s7 |
| food | 576 | 0.06 | 0-0.57 | cluster_s7 |
| food | 577 | 0.06 | 0-0.56 | cluster_s7 |
| food | 578 | 0.06 | 0-0.61 | cluster_s7 |
| food | 579 | 0.06 | 0-0.54 | cluster_s7 |
| food | 580 | 0.06 | 0-0.55 | cluster_s7 |
| food | 581 | 0.06 | 0-0.62 | cluster_s7 |
| food | 582 | 0.06 | 0-0.54 | cluster_s7 |
| food | 583 | 0.06 | 0-0.69 | cluster_s7 |
| food | 584 | 0.06 | 0-0.56 | cluster_s7 |
| food | 585 | 0.06 | 0-0.55 | cluster_s7 |
| food | 586 | 0.06 | 0-0.64 | cluster_s7 |
| food | 587 | 0.06 | 0-0.49 | cluster_s7 |
| food | 588 | 0.06 | 0-0.64 | cluster_s7 |
| food | 589 | 0.06 | 0-0.63 | cluster_s7 |
| food | 590 | 0.06 | 0-0.51 | cluster_s7 |
| food | 591 | 0.06 | 0-0.62 | cluster_s7 |
| food | 592 | 0.06 | 0-0.57 | cluster_s7 |
| food | 593 | 0.06 | 0-0.52 | cluster_s7 |
| food | 594 | 0.06 | 0-0.72 | cluster_s7 |
| food | 595 | 0.06 | 0-0.6 | cluster_s7 |
| food | 596 | 0.06 | 0-0.62 | cluster_s7 |
| food | 597 | 0.06 | 0-0.64 | cluster_s7 |
| food | 598 | 0.06 | 0-0.58 | cluster_s7 |
| food | 599 | 0.06 | 0-0.69 | cluster_s7 |
| food | 600 | 0.07 | 0-0.57 | cluster_s7 |
| food | 601 | 0.06 | 0-0.7 | cluster_s7 |
| food | 602 | 0.06 | 0-0.6 | cluster_s7 |
| food | 603 | 0.06 | 0-0.57 | cluster_s7 |
| food | 604 | 0.06 | 0-0.6 | cluster_s7 |
| food | 605 | 0.06 | 0-0.5 | cluster_s7 |
| food | 606 | 0.06 | 0-0.58 | cluster_s7 |
| food | 607 | 0.06 | 0-0.61 | cluster_s7 |
| food | 608 | 0.06 | 0-0.61 | cluster_s7 |
| food | 609 | 0.06 | 0-0.64 | cluster_s7 |
| food | 610 | 0.06 | 0-0.62 | cluster_s7 |
| food | 611 | 0.13 | 0-0.76 | cluster_s7 |
| food | 612 | 0.06 | 0-0.74 | cluster_s7 |
| food | 613 | 1.15 | 0.4-2.97 | cluster_s7 |
| food | 614 | 0.06 | 0-0.58 | cluster_s7 |
| food | 615 | 0.26 | 0.02-1.01 | cluster_s7 |
| food | 616 | 0.13 | 0-0.92 | cluster_s7 |
| food | 617 | 0.13 | 0-0.88 | cluster_s7 |
| food | 618 | 0.13 | 0-0.7 | cluster_s7 |
| food | 619 | 0.13 | 0-0.82 | cluster_s7 |
| food | 620 | 0.13 | 0-0.92 | cluster_s7 |
| food | 621 | 0.13 | 0-0.83 | cluster_s7 |
| food | 622 | 0.13 | 0-0.76 | cluster_s7 |
| food | 623 | 0.51 | 0.08-1.61 | cluster_s7 |
| food | 624 | 0.13 | 0-0.68 | cluster_s7 |
| food | 625 | 0.25 | 0.02-0.98 | cluster_s7 |
| food | 626 | 0.13 | 0-0.72 | cluster_s7 |
| food | 627 | 0.13 | 0-0.88 | cluster_s7 |
| food | 628 | 0.13 | 0-0.7 | cluster_s7 |
| food | 629 | 0.13 | 0-0.72 | cluster_s7 |
| food | 630 | 0.13 | 0-0.94 | cluster_s7 |
| food | 631 | 0.13 | 0-0.74 | cluster_s7 |
| food | 632 | 0.13 | 0-0.77 | cluster_s7 |
| food | 633 | 0.19 | 0.01-0.89 | cluster_s7 |
| food | 634 | 0.13 | 0-0.87 | cluster_s7 |
| food | 635 | 0.13 | 0-0.86 | cluster_s7 |
| food | 636 | 0.25 | 0.01-0.97 | cluster_s7 |
| food | 637 | 0.06 | 0-0.62 | cluster_s7 |
| food | 638 | 0.06 | 0-0.62 | cluster_s7 |
| food | 639 | 0.06 | 0-0.62 | cluster_s7 |
| food | 640 | 0.07 | 0-0.73 | cluster_s7 |
| food | 641 | 0.06 | 0-0.64 | cluster_s7 |
| food | 642 | 0.06 | 0-0.7 | cluster_s7 |
| food | 643 | 0.06 | 0-0.58 | cluster_s7 |
| food | 644 | 0.06 | 0-0.5 | cluster_s7 |
| food | 645 | 0.06 | 0-0.69 | cluster_s7 |
| food | 646 | 0.06 | 0-0.59 | cluster_s7 |
| food | 647 | 0.06 | 0-0.63 | cluster_s7 |
| food | 648 | 0.06 | 0-0.54 | cluster_s7 |
| food | 649 | 0.06 | 0-0.64 | cluster_s7 |
| food | 650 | 0.06 | 0-0.67 | cluster_s7 |
| food | 651 | 0.06 | 0-0.65 | cluster_s7 |
| food | 652 | 0.06 | 0-0.67 | cluster_s7 |
| food | 653 | 0.06 | 0-0.65 | cluster_s7 |
| food | 654 | 0.06 | 0-0.55 | cluster_s7 |
| food | 655 | 0.06 | 0-0.62 | cluster_s7 |
| food | 656 | 0.06 | 0-0.65 | cluster_s7 |
| food | 657 | 0.06 | 0-0.69 | cluster_s7 |
| food | 658 | 0.06 | 0-0.72 | cluster_s7 |
| food | 659 | 0.06 | 0-0.5 | cluster_s7 |
| food | 660 | 0.06 | 0-0.53 | cluster_s7 |
| food | 661 | 0.06 | 0-0.52 | cluster_s7 |
| food | 662 | 0.06 | 0-0.57 | cluster_s7 |
| food | 663 | 0.06 | 0-0.58 | cluster_s7 |
| food | 664 | 0.06 | 0-0.59 | cluster_s7 |
| food | 665 | 0.06 | 0-0.63 | cluster_s7 |
| food | 666 | 0.06 | 0-0.56 | cluster_s7 |
| food | 667 | 0.06 | 0-0.7 | cluster_s7 |
| food | 668 | 0.06 | 0-0.66 | cluster_s7 |
| food | 669 | 0.06 | 0-0.63 | cluster_s7 |
| food | 670 | 0.06 | 0-0.7 | cluster_s7 |
| food | 671 | 0.06 | 0-0.72 | cluster_s7 |
| food | 672 | 0.06 | 0-0.64 | cluster_s7 |
| food | 673 | 0.06 | 0-0.66 | cluster_s7 |
| food | 674 | 0.06 | 0-0.61 | cluster_s7 |
| food | 675 | 0.13 | 0-0.8 | cluster_s7 |
| food | 676 | 0.13 | 0-0.78 | cluster_s7 |
| food | 677 | 0.13 | 0-0.76 | cluster_s7 |
| food | 678 | 0.13 | 0-0.89 | cluster_s7 |
| food | 679 | 0.13 | 0-0.76 | cluster_s7 |
| food | 680 | 0.13 | 0-0.81 | cluster_s7 |
| food | 681 | 0.13 | 0-0.88 | cluster_s7 |
| food | 682 | 0.13 | 0-0.76 | cluster_s7 |
| food | 683 | 0.13 | 0-0.75 | cluster_s7 |
| food | 684 | 0.13 | 0-0.78 | cluster_s7 |
| food | 685 | 0.13 | 0-0.75 | cluster_s7 |
| food | 686 | 0.13 | 0-0.97 | cluster_s7 |
| food | 687 | 0.13 | 0-0.7 | cluster_s7 |
| food | 688 | 0.13 | 0-0.72 | cluster_s7 |
| food | 689 | 0.13 | 0-0.83 | cluster_s7 |
| food | 690 | 0.06 | 0-0.67 | cluster_s7 |
| food | 691 | 0.06 | 0-0.56 | cluster_s7 |
| food | 692 | 0.06 | 0-0.62 | cluster_s7 |
| food | 693 | 0.06 | 0-0.54 | cluster_s7 |
| food | 694 | 0.06 | 0-0.65 | cluster_s7 |
| food | 695 | 0.06 | 0-0.64 | cluster_s7 |
| food | 696 | 0.06 | 0-0.75 | cluster_s7 |
| food | 697 | 0.06 | 0-0.7 | cluster_s7 |
| food | 698 | 0.06 | 0-0.57 | cluster_s7 |
| food | 699 | 0.06 | 0-0.57 | cluster_s7 |
| food | 700 | 0.06 | 0-0.53 | cluster_s7 |
| food | 701 | 0.06 | 0-0.53 | cluster_s7 |
| food | 702 | 0.06 | 0-0.56 | cluster_s7 |
| food | 703 | 0.06 | 0-0.6 | cluster_s7 |
| food | 704 | 0.06 | 0-0.52 | cluster_s7 |
| food | 705 | 0.06 | 0-0.78 | cluster_s7 |
| food | 706 | 0.06 | 0-0.56 | cluster_s7 |
| food | 707 | 0.06 | 0-0.72 | cluster_s7 |
| food | 708 | 0.13 | 0-0.65 | cluster_s7 |
| food | 709 | 0.13 | 0-0.66 | cluster_s7 |
| food | 710 | 0.13 | 0-0.8 | cluster_s7 |
| food | 711 | 0.06 | 0-0.52 | cluster_s7 |
| food | 712 | 0.06 | 0-0.61 | cluster_s7 |
| food | 713 | 0.06 | 0-0.73 | cluster_s7 |
| food | 714 | 0.06 | 0-0.63 | cluster_s7 |
| food | 715 | 0.06 | 0-0.63 | cluster_s7 |
| food | 716 | 0.13 | 0-0.74 | cluster_s7 |
| food | 717 | 0.06 | 0-0.52 | cluster_s7 |
| food | 718 | 0.06 | 0-0.54 | cluster_s7 |
| food | 719 | 0.06 | 0-0.7 | cluster_s7 |
| food | 720 | 0.06 | 0-0.58 | cluster_s7 |
| food | 721 | 0.06 | 0-0.67 | cluster_s7 |
| food | 722 | 0.06 | 0-0.55 | cluster_s7 |
| food | 723 | 0.06 | 0-0.73 | cluster_s7 |
| food | 724 | 0.06 | 0-0.72 | cluster_s7 |
| food | 725 | 0.06 | 0-0.68 | cluster_s7 |
| food | 726 | 0.06 | 0-0.79 | cluster_s7 |
| food | 727 | 0.06 | 0-0.58 | cluster_s7 |
| food | 728 | 0.07 | 0-0.54 | cluster_s7 |
| food | 729 | 0.06 | 0-0.63 | cluster_s7 |
| food | 730 | 0.13 | 0-0.77 | cluster_s7 |
| food | 731 | 0.19 | 0.01-0.81 | cluster_s7 |
| food | 732 | 0.06 | 0-0.53 | cluster_s7 |
| food | 733 | 0.13 | 0-0.75 | cluster_s7 |
| food | 734 | 0.13 | 0-0.79 | cluster_s7 |
| food | 735 | 0.13 | 0-0.73 | cluster_s7 |
| food | 736 | 0.13 | 0-0.75 | cluster_s7 |
| food | 737 | 0.13 | 0-0.74 | cluster_s7 |
| food | 738 | 0.06 | 0-0.74 | cluster_s7 |
| food | 739 | 0.13 | 0-0.91 | cluster_s7 |
| food | 740 | 0.19 | 0.01-0.84 | cluster_s7 |
| food | 741 | 0.06 | 0-0.55 | cluster_s7 |
| food | 742 | 0.06 | 0-0.56 | cluster_s7 |
| food | 743 | 0.06 | 0-0.64 | cluster_s7 |
| food | 744 | 0.06 | 0-0.56 | cluster_s7 |
| food | 745 | 0.06 | 0-0.55 | cluster_s7 |
| food | 746 | 0.06 | 0-0.86 | cluster_s7 |
| food | 747 | 0.13 | 0-0.69 | cluster_s7 |
| food | 748 | 0.13 | 0-0.88 | cluster_s7 |
| food | 749 | 0.06 | 0-0.63 | cluster_s7 |
| food | 750 | 0.07 | 0-0.72 | cluster_s7 |
| food | 751 | 0.06 | 0-0.57 | cluster_s7 |
| food | 752 | 0.13 | 0-0.76 | cluster_s7 |
| food | 753 | 0.06 | 0-0.58 | cluster_s7 |
| food | 754 | 0.06 | 0-0.49 | cluster_s7 |
| food | 755 | 0.13 | 0-0.67 | cluster_s7 |
| food | 756 | 0.13 | 0-0.73 | cluster_s7 |
| food | 757 | 0.06 | 0-0.65 | cluster_s7 |
| food | 758 | 0.06 | 0-0.77 | cluster_s7 |
| food | 759 | 0.06 | 0-0.52 | cluster_s7 |
| food | 760 | 0.06 | 0-0.93 | cluster_s7 |
| food | 761 | 0.06 | 0-0.64 | cluster_s7 |
| food | 762 | 0.13 | 0-0.9 | cluster_s7 |
| food | 763 | 0.06 | 0-0.66 | cluster_s7 |
| food | 764 | 0.06 | 0-0.69 | cluster_s7 |
| food | 765 | 0.06 | 0-0.61 | cluster_s7 |
| food | 766 | 0.06 | 0-0.6 | cluster_s7 |
| food | 767 | 0.06 | 0-0.56 | cluster_s7 |
| food | 768 | 0.06 | 0-0.5 | cluster_s7 |
| food | 769 | 0.06 | 0-0.65 | cluster_s7 |
| food | 770 | 0.06 | 0-0.55 | cluster_s7 |
| food | 771 | 0.06 | 0-0.57 | cluster_s7 |
| food | 772 | 0.07 | 0-0.76 | cluster_s7 |
| food | 773 | 0.06 | 0-0.54 | cluster_s7 |
| food | 774 | 0.06 | 0-0.63 | cluster_s7 |
| food | 775 | 0.19 | 0.01-1.06 | cluster_s7 |
| food | 776 | 0.13 | 0-0.68 | cluster_s7 |
| food | 777 | 0.13 | 0-0.74 | cluster_s7 |
| food | 778 | 0.13 | 0-0.7 | cluster_s7 |
| food | 779 | 0.13 | 0-0.78 | cluster_s7 |
| food | 780 | 0.19 | 0.01-0.89 | cluster_s7 |
| food | 781 | 0.13 | 0-0.88 | cluster_s7 |
| food | 782 | 0.13 | 0-0.69 | cluster_s7 |
| food | 783 | 0.13 | 0-0.73 | cluster_s7 |
| food | 784 | 0.26 | 0.02-1 | cluster_s7 |
| food | 785 | 0.13 | 0-0.74 | cluster_s7 |
| food | 786 | 0.13 | 0-0.66 | cluster_s7 |
| food | 787 | 0.13 | 0-0.92 | cluster_s7 |
| food | 788 | 0.13 | 0-0.75 | cluster_s7 |
| food | 789 | 0.13 | 0-0.84 | cluster_s7 |
| food | 790 | 0.13 | 0-0.88 | cluster_s7 |
| food | 791 | 0.13 | 0-0.93 | cluster_s7 |
| food | 792 | 0.13 | 0-0.75 | cluster_s7 |
| food | 793 | 0.13 | 0-0.78 | cluster_s7 |
| food | 794 | 0.13 | 0-0.78 | cluster_s7 |
| food | 795 | 0.13 | 0-0.66 | cluster_s7 |
| food | 796 | 0.12 | 0-0.86 | cluster_s7 |
| human | 1 | 0.06 | 0-0.55 | cluster_s7 |
| human | 2 | 0.06 | 0-0.76 | cluster_s7 |
| human | 3 | 0.06 | 0-0.6 | cluster_s7 |
| human | 4 | 0.13 | 0-0.78 | cluster_s7 |
| human | 5 | 0.13 | 0-0.68 | cluster_s7 |
| human | 6 | 0.13 | 0-0.81 | cluster_s7 |
| human | 7 | 0.13 | 0-0.7 | cluster_s7 |
| human | 8 | 0.13 | 0-0.81 | cluster_s7 |
| human | 9 | 0.13 | 0-0.81 | cluster_s7 |
| human | 10 | 0.13 | 0-0.76 | cluster_s7 |
| human | 11 | 0.13 | 0-0.84 | cluster_s7 |
| human | 12 | 0.19 | 0-0.9 | cluster_s7 |
| human | 13 | 0.13 | 0-0.85 | cluster_s7 |
| human | 14 | 0.13 | 0-0.74 | cluster_s7 |
| human | 15 | 0.13 | 0-0.84 | cluster_s7 |
| human | 16 | 0.38 | 0.04-1.29 | cluster_s7 |
| human | 17 | 0.13 | 0-0.81 | cluster_s7 |
| human | 18 | 0.13 | 0-0.75 | cluster_s7 |
| human | 19 | 0.19 | 0-0.92 | cluster_s7 |
| human | 20 | 0.13 | 0-0.78 | cluster_s7 |
| human | 21 | 0.13 | 0-0.72 | cluster_s7 |
| human | 22 | 0.13 | 0-0.69 | cluster_s7 |
| human | 23 | 0.32 | 0.04-1.26 | cluster_s7 |
| human | 24 | 0.13 | 0-0.83 | cluster_s7 |
| human | 25 | 0.13 | 0-0.93 | cluster_s7 |
| human | 26 | 0.13 | 0-0.79 | cluster_s7 |
| human | 27 | 0.45 | 0.05-1.58 | cluster_s7 |
| human | 28 | 0.13 | 0-0.77 | cluster_s7 |
| human | 29 | 0.26 | 0.01-0.94 | cluster_s7 |
| human | 30 | 0.13 | 0-0.75 | cluster_s7 |
| human | 31 | 0.13 | 0-0.65 | cluster_s7 |
| human | 32 | 0.26 | 0.02-1 | cluster_s7 |
| human | 33 | 0.13 | 0-0.75 | cluster_s7 |
| human | 34 | 0.06 | 0-0.6 | cluster_s7 |
| human | 35 | 0.06 | 0-0.7 | cluster_s7 |
| human | 36 | 0.06 | 0-0.64 | cluster_s7 |
| human | 37 | 0.06 | 0-0.52 | cluster_s7 |
| human | 38 | 0.06 | 0-0.7 | cluster_s7 |
| human | 39 | 0.06 | 0-0.58 | cluster_s7 |
| human | 40 | 0.06 | 0-0.52 | cluster_s7 |
| human | 41 | 0.06 | 0-0.45 | cluster_s7 |
| human | 42 | 0.57 | 0.14-1.62 | cluster_s7 |
| human | 43 | 0.06 | 0-0.61 | cluster_s7 |
| human | 44 | 0.06 | 0-0.53 | cluster_s7 |
| human | 45 | 0.06 | 0-0.51 | cluster_s7 |
| human | 46 | 0.32 | 0.04-1.16 | cluster_s7 |
| human | 47 | 0.07 | 0-0.52 | cluster_s7 |
| human | 48 | 0.07 | 0-0.6 | cluster_s7 |
| human | 49 | 0.06 | 0-0.57 | cluster_s7 |
| human | 50 | 0.07 | 0-0.72 | cluster_s7 |
| human | 51 | 0.06 | 0-0.58 | cluster_s7 |
| human | 52 | 0.06 | 0-0.55 | cluster_s7 |
| human | 53 | 0.06 | 0-0.53 | cluster_s7 |
| human | 54 | 0.06 | 0-0.56 | cluster_s7 |
| human | 55 | 0.07 | 0-0.63 | cluster_s7 |
| human | 56 | 0.06 | 0-0.69 | cluster_s7 |
| human | 57 | 0.07 | 0-0.61 | cluster_s7 |
| human | 58 | 0.06 | 0-0.76 | cluster_s7 |
| human | 59 | 0.06 | 0-0.51 | cluster_s7 |
| human | 60 | 0.06 | 0-0.68 | cluster_s7 |
| human | 61 | 0.06 | 0-0.6 | cluster_s7 |
| human | 62 | 0.06 | 0-0.56 | cluster_s7 |
| human | 63 | 0.06 | 0-0.62 | cluster_s7 |
| human | 64 | 0.06 | 0-0.68 | cluster_s7 |
| human | 65 | 0.07 | 0-0.7 | cluster_s7 |
| human | 66 | 0.06 | 0-0.52 | cluster_s7 |
| human | 67 | 0.06 | 0-0.68 | cluster_s7 |
| human | 68 | 0.06 | 0-0.76 | cluster_s7 |
| human | 69 | 0.06 | 0-0.63 | cluster_s7 |
| human | 70 | 0.06 | 0-0.67 | cluster_s7 |
| human | 71 | 0.07 | 0-0.53 | cluster_s7 |
| human | 72 | 0.06 | 0-0.6 | cluster_s7 |
| human | 73 | 0.06 | 0-0.62 | cluster_s7 |
| human | 74 | 0.06 | 0-0.61 | cluster_s7 |
| human | 75 | 0.06 | 0-0.73 | cluster_s7 |
| human | 76 | 0.06 | 0-0.56 | cluster_s7 |
| human | 77 | 0.07 | 0-0.53 | cluster_s7 |
| human | 78 | 0.06 | 0-0.58 | cluster_s7 |
| human | 79 | 0.07 | 0-0.64 | cluster_s7 |
| human | 80 | 0.07 | 0-0.62 | cluster_s7 |
| human | 81 | 0.06 | 0-0.6 | cluster_s7 |
| human | 82 | 0.06 | 0-0.8 | cluster_s7 |
| human | 83 | 0.06 | 0-0.64 | cluster_s7 |
| human | 84 | 0.06 | 0-0.54 | cluster_s7 |
| human | 85 | 0.07 | 0-0.67 | cluster_s7 |
| human | 86 | 0.06 | 0-0.71 | cluster_s7 |
| human | 87 | 0.06 | 0-0.58 | cluster_s7 |
| human | 88 | 0.06 | 0-0.88 | cluster_s7 |
| human | 89 | 0.06 | 0-0.51 | cluster_s7 |
| human | 90 | 0.07 | 0-0.56 | cluster_s7 |
| human | 91 | 0.06 | 0-0.62 | cluster_s7 |
| human | 92 | 0.06 | 0-0.58 | cluster_s7 |
| human | 93 | 0.06 | 0-0.75 | cluster_s7 |
| human | 94 | 0.07 | 0-0.58 | cluster_s7 |
| human | 95 | 0.06 | 0-0.64 | cluster_s7 |
| human | 96 | 0.06 | 0-0.67 | cluster_s7 |
| human | 97 | 0.06 | 0-0.56 | cluster_s7 |
| human | 98 | 0.06 | 0-0.58 | cluster_s7 |
| human | 99 | 0.06 | 0-0.66 | cluster_s7 |
| human | 100 | 0.06 | 0-0.52 | cluster_s7 |
| human | 101 | 0.06 | 0-0.58 | cluster_s7 |
| human | 102 | 0.13 | 0-0.78 | cluster_s7 |
| human | 103 | 0.13 | 0-0.77 | cluster_s7 |
| human | 104 | 0.13 | 0-0.91 | cluster_s7 |
| human | 105 | 0.13 | 0-0.73 | cluster_s7 |
| human | 106 | 0.39 | 0.04-1.36 | cluster_s7 |
| human | 107 | 0.13 | 0-0.92 | cluster_s7 |
| human | 108 | 0.13 | 0-0.74 | cluster_s7 |
| human | 109 | 0.64 | 0.16-1.7 | cluster_s7 |
| human | 110 | 0.13 | 0-0.79 | cluster_s7 |
| human | 111 | 0.19 | 0.01-0.94 | cluster_s7 |
| human | 112 | 0.19 | 0-0.89 | cluster_s7 |
| human | 113 | 0.13 | 0-0.68 | cluster_s7 |
| human | 114 | 0.13 | 0-0.84 | cluster_s7 |
| human | 115 | 0.13 | 0-0.91 | cluster_s7 |
| human | 116 | 0.13 | 0-0.75 | cluster_s7 |
| human | 117 | 0.13 | 0-0.7 | cluster_s7 |
| human | 118 | 0.19 | 0-0.9 | cluster_s7 |
| human | 119 | 0.13 | 0-0.85 | cluster_s7 |
| human | 120 | 0.13 | 0-0.89 | cluster_s7 |
| human | 121 | 0.13 | 0-0.81 | cluster_s7 |
| human | 122 | 0.13 | 0-0.84 | cluster_s7 |
| human | 123 | 0.13 | 0-0.79 | cluster_s7 |
| human | 124 | 0.13 | 0-0.8 | cluster_s7 |
| human | 125 | 0.13 | 0-0.8 | cluster_s7 |
| human | 126 | 0.52 | 0.09-1.49 | cluster_s7 |
| human | 127 | 0.13 | 0-0.83 | cluster_s7 |
| human | 128 | 0.13 | 0-0.76 | cluster_s7 |
| human | 129 | 0.13 | 0-0.78 | cluster_s7 |
| human | 130 | 0.13 | 0-0.96 | cluster_s7 |
| human | 131 | 0.13 | 0-0.83 | cluster_s7 |
| human | 132 | 0.13 | 0-0.89 | cluster_s7 |
| human | 133 | 0.13 | 0-0.91 | cluster_s7 |
| human | 134 | 0.13 | 0-0.7 | cluster_s7 |
| human | 135 | 0.19 | 0.01-0.95 | cluster_s7 |
| human | 136 | 0.26 | 0.02-1.3 | cluster_s7 |
| human | 137 | 0.13 | 0-0.82 | cluster_s7 |
| human | 138 | 0.06 | 0-0.72 | cluster_s7 |
| human | 139 | 0.06 | 0-0.54 | cluster_s7 |
| human | 140 | 0.06 | 0-0.58 | cluster_s7 |
| human | 141 | 0.07 | 0-0.7 | cluster_s7 |
| human | 142 | 0.06 | 0-0.6 | cluster_s7 |
| human | 143 | 0.06 | 0-0.59 | cluster_s7 |
| human | 144 | 0.06 | 0-0.79 | cluster_s7 |
| human | 145 | 0.07 | 0-0.6 | cluster_s7 |
| human | 146 | 0.06 | 0-0.54 | cluster_s7 |
| human | 147 | 0.06 | 0-0.63 | cluster_s7 |
| human | 148 | 0.06 | 0-0.5 | cluster_s7 |
| human | 149 | 0.06 | 0-0.55 | cluster_s7 |
| human | 150 | 0.06 | 0-0.54 | cluster_s7 |
| human | 151 | 0.06 | 0-0.58 | cluster_s7 |
| human | 152 | 0.06 | 0-0.62 | cluster_s7 |
| human | 153 | 0.06 | 0-0.71 | cluster_s7 |
| human | 154 | 0.06 | 0-0.68 | cluster_s7 |
| human | 155 | 0.07 | 0-0.68 | cluster_s7 |
| human | 156 | 0.06 | 0-0.8 | cluster_s7 |
| human | 157 | 0.06 | 0-0.63 | cluster_s7 |
| human | 158 | 0.06 | 0-0.56 | cluster_s7 |
| human | 159 | 0.06 | 0-0.6 | cluster_s7 |
| human | 160 | 0.06 | 0-0.54 | cluster_s7 |
| human | 161 | 0.06 | 0-0.58 | cluster_s7 |
| human | 162 | 0.07 | 0-0.6 | cluster_s7 |
| human | 163 | 0.13 | 0-0.77 | cluster_s7 |
| human | 164 | 0.06 | 0-0.77 | cluster_s7 |
| human | 165 | 0.06 | 0-0.62 | cluster_s7 |
| human | 166 | 0.06 | 0-0.61 | cluster_s7 |
| human | 167 | 0.07 | 0-0.81 | cluster_s7 |
| human | 168 | 0.06 | 0-0.58 | cluster_s7 |
| human | 169 | 0.06 | 0-0.53 | cluster_s7 |
| human | 170 | 0.06 | 0-0.52 | cluster_s7 |
| human | 171 | 0.06 | 0-0.64 | cluster_s7 |
| human | 172 | 0.06 | 0-0.53 | cluster_s7 |
| human | 173 | 0.06 | 0-0.58 | cluster_s7 |
| human | 174 | 0.06 | 0-0.56 | cluster_s7 |
| human | 175 | 0.06 | 0-0.6 | cluster_s7 |
| human | 176 | 0.06 | 0-0.7 | cluster_s7 |
| human | 177 | 0.07 | 0-0.56 | cluster_s7 |
| human | 178 | 0.06 | 0-0.71 | cluster_s7 |
| human | 179 | 0.06 | 0-0.72 | cluster_s7 |
| human | 180 | 0.06 | 0-0.6 | cluster_s7 |
| human | 181 | 0.06 | 0-0.62 | cluster_s7 |
| human | 182 | 0.07 | 0-0.66 | cluster_s7 |
| human | 183 | 0.06 | 0-0.58 | cluster_s7 |
| human | 184 | 0.13 | 0-0.82 | cluster_s7 |
| human | 185 | 0.07 | 0-0.52 | cluster_s7 |
| human | 186 | 0.06 | 0-0.57 | cluster_s7 |
| human | 187 | 0.07 | 0-0.51 | cluster_s7 |
| human | 188 | 0.06 | 0-0.55 | cluster_s7 |
| human | 189 | 0.06 | 0-0.65 | cluster_s7 |
| human | 190 | 0.06 | 0-0.6 | cluster_s7 |
| human | 191 | 0.06 | 0-0.56 | cluster_s7 |
| human | 192 | 0.06 | 0-0.64 | cluster_s7 |
| human | 193 | 0.06 | 0-0.74 | cluster_s7 |
| human | 194 | 0.06 | 0-0.53 | cluster_s7 |
| human | 195 | 0.13 | 0-0.94 | cluster_s7 |
| human | 196 | 0.13 | 0-0.72 | cluster_s7 |
| human | 197 | 0.20 | 0.01-0.86 | cluster_s7 |
| human | 198 | 0.13 | 0-0.68 | cluster_s7 |
| human | 199 | 0.13 | 0-0.93 | cluster_s7 |
| human | 200 | 0.13 | 0-0.8 | cluster_s7 |
| human | 201 | 0.13 | 0-0.84 | cluster_s7 |
| human | 202 | 0.13 | 0-0.77 | cluster_s7 |
| human | 203 | 0.19 | 0-0.84 | cluster_s7 |
| human | 204 | 0.13 | 0-0.82 | cluster_s7 |
| human | 205 | 0.13 | 0-0.81 | cluster_s7 |
| human | 206 | 0.13 | 0-0.98 | cluster_s7 |
| human | 207 | 0.13 | 0-0.77 | cluster_s7 |
| human | 208 | 1.09 | 0.35-2.38 | cluster_s7 |
| human | 209 | 0.19 | 0-0.97 | cluster_s7 |
| human | 210 | 0.13 | 0-0.67 | cluster_s7 |
| human | 211 | 0.13 | 0-0.79 | cluster_s7 |
| human | 212 | 0.13 | 0-0.68 | cluster_s7 |
| human | 213 | 0.45 | 0.05-1.65 | cluster_s7 |
| human | 214 | 0.13 | 0-0.66 | cluster_s7 |
| human | 215 | 0.13 | 0-0.9 | cluster_s7 |
| human | 216 | 0.13 | 0-0.81 | cluster_s7 |
| human | 217 | 0.13 | 0-0.98 | cluster_s7 |
| human | 218 | 0.06 | 0-0.67 | cluster_s7 |
| human | 219 | 0.06 | 0-0.64 | cluster_s7 |
| human | 220 | 0.06 | 0-0.58 | cluster_s7 |
| human | 221 | 0.06 | 0-0.52 | cluster_s7 |
| human | 222 | 0.06 | 0-0.66 | cluster_s7 |
| human | 223 | 0.06 | 0-0.6 | cluster_s7 |
| human | 224 | 0.07 | 0-0.72 | cluster_s7 |
| human | 225 | 0.06 | 0-0.61 | cluster_s7 |
| human | 226 | 0.06 | 0-0.59 | cluster_s7 |
| human | 227 | 0.07 | 0-0.57 | cluster_s7 |
| human | 228 | 0.07 | 0-1.03 | cluster_s7 |
| human | 229 | 0.06 | 0-0.73 | cluster_s7 |
| human | 230 | 0.06 | 0-0.73 | cluster_s7 |
| human | 231 | 0.07 | 0-0.66 | cluster_s7 |
| human | 232 | 0.07 | 0-0.58 | cluster_s7 |
| human | 233 | 0.06 | 0-0.8 | cluster_s7 |
| human | 234 | 0.06 | 0-0.59 | cluster_s7 |
| human | 235 | 0.06 | 0-0.58 | cluster_s7 |
| human | 236 | 0.06 | 0-0.59 | cluster_s7 |
| human | 237 | 0.06 | 0-0.56 | cluster_s7 |
| human | 238 | 0.06 | 0-0.61 | cluster_s7 |
| human | 239 | 0.06 | 0-0.62 | cluster_s7 |
| human | 240 | 0.07 | 0-0.55 | cluster_s7 |
| human | 241 | 0.06 | 0-0.55 | cluster_s7 |
| human | 242 | 0.06 | 0-0.7 | cluster_s7 |
| human | 243 | 0.06 | 0-0.74 | cluster_s7 |
| human | 244 | 0.06 | 0-0.57 | cluster_s7 |
| human | 245 | 0.06 | 0-0.58 | cluster_s7 |
| human | 246 | 0.06 | 0-0.63 | cluster_s7 |
| human | 247 | 0.06 | 0-0.55 | cluster_s7 |
| human | 248 | 0.06 | 0-0.71 | cluster_s7 |
| human | 249 | 0.07 | 0-0.63 | cluster_s7 |
| human | 250 | 0.13 | 0-0.78 | cluster_s7 |
| human | 251 | 0.13 | 0-0.88 | cluster_s7 |
| human | 252 | 0.13 | 0-0.86 | cluster_s7 |
| human | 253 | 0.58 | 0.1-1.53 | cluster_s7 |
| human | 254 | 0.13 | 0-0.7 | cluster_s7 |
| human | 255 | 0.13 | 0-0.87 | cluster_s7 |
| human | 256 | 0.13 | 0-0.7 | cluster_s7 |
| human | 257 | 0.13 | 0-0.87 | cluster_s7 |
| human | 258 | 0.13 | 0-0.83 | cluster_s7 |
| human | 259 | 0.45 | 0.06-1.63 | cluster_s7 |
| human | 260 | 0.13 | 0-0.87 | cluster_s7 |
| human | 261 | 0.13 | 0-0.78 | cluster_s7 |
| human | 262 | 0.13 | 0-0.74 | cluster_s7 |
| human | 263 | 0.13 | 0-0.81 | cluster_s7 |
| human | 264 | 0.20 | 0-0.9 | cluster_s7 |
| human | 265 | 0.13 | 0-0.84 | cluster_s7 |
| human | 266 | 0.19 | 0.01-0.93 | cluster_s7 |
| human | 267 | 0.13 | 0-0.78 | cluster_s7 |
| human | 268 | 0.13 | 0-0.82 | cluster_s7 |
| human | 269 | 0.13 | 0-0.77 | cluster_s7 |
| human | 270 | 0.13 | 0-0.72 | cluster_s7 |
| human | 271 | 0.26 | 0.02-1 | cluster_s7 |
| human | 272 | 0.06 | 0-0.6 | cluster_s7 |
| human | 273 | 0.06 | 0-0.71 | cluster_s7 |
| human | 274 | 0.06 | 0-0.64 | cluster_s7 |
| human | 275 | 0.06 | 0-0.59 | cluster_s7 |
| human | 276 | 0.06 | 0-0.6 | cluster_s7 |
| human | 277 | 0.07 | 0-0.7 | cluster_s7 |
| human | 278 | 0.07 | 0-0.63 | cluster_s7 |
| human | 279 | 0.06 | 0-0.63 | cluster_s7 |
| human | 280 | 0.06 | 0-0.5 | cluster_s7 |
| human | 281 | 0.06 | 0-0.56 | cluster_s7 |
| human | 282 | 0.06 | 0-0.81 | cluster_s7 |
| human | 283 | 0.06 | 0-0.54 | cluster_s7 |
| human | 284 | 0.06 | 0-0.56 | cluster_s7 |
| human | 285 | 0.06 | 0-0.63 | cluster_s7 |
| human | 286 | 0.06 | 0-0.6 | cluster_s7 |
| human | 287 | 0.06 | 0-0.87 | cluster_s7 |
| human | 288 | 0.06 | 0-0.67 | cluster_s7 |
| human | 289 | 0.06 | 0-0.63 | cluster_s7 |
| human | 290 | 0.07 | 0-0.59 | cluster_s7 |
| human | 291 | 0.19 | 0.01-0.99 | cluster_s7 |
| human | 292 | 0.13 | 0-0.89 | cluster_s7 |
| human | 293 | 0.32 | 0.02-1.07 | cluster_s7 |
| human | 294 | 1.10 | 0.38-2.67 | cluster_s7 |
| human | 295 | 0.13 | 0-0.9 | cluster_s7 |
| human | 296 | 0.13 | 0-0.69 | cluster_s7 |
| human | 297 | 0.13 | 0-0.72 | cluster_s7 |
| human | 298 | 0.13 | 0-0.84 | cluster_s7 |
| human | 299 | 0.26 | 0.01-1.14 | cluster_s7 |
| human | 300 | 0.13 | 0-0.71 | cluster_s7 |
| human | 301 | 0.13 | 0-0.67 | cluster_s7 |
| human | 302 | 0.13 | 0-0.67 | cluster_s7 |
| human | 303 | 0.13 | 0-0.75 | cluster_s7 |
| human | 304 | 0.06 | 0-0.57 | cluster_s7 |
| human | 305 | 0.07 | 0-0.63 | cluster_s7 |
| human | 306 | 0.13 | 0-0.77 | cluster_s7 |
| human | 307 | 0.06 | 0-0.59 | cluster_s7 |
| human | 308 | 0.06 | 0-0.6 | cluster_s7 |
| human | 309 | 0.07 | 0-0.6 | cluster_s7 |
| human | 310 | 0.06 | 0-0.61 | cluster_s7 |
| human | 311 | 0.06 | 0-0.68 | cluster_s7 |
| human | 312 | 0.06 | 0-0.61 | cluster_s7 |
| human | 313 | 0.06 | 0-0.53 | cluster_s7 |
| human | 314 | 0.06 | 0-0.82 | cluster_s7 |
| human | 315 | 0.06 | 0-0.6 | cluster_s7 |
| human | 316 | 0.07 | 0-0.6 | cluster_s7 |
| human | 317 | 0.06 | 0-0.56 | cluster_s7 |
| human | 318 | 0.06 | 0-0.69 | cluster_s7 |
| human | 319 | 0.06 | 0-0.62 | cluster_s7 |
| human | 320 | 0.06 | 0-0.83 | cluster_s7 |
| human | 321 | 0.06 | 0-0.62 | cluster_s7 |
| human | 322 | 0.06 | 0-0.59 | cluster_s7 |
| human | 323 | 0.06 | 0-0.63 | cluster_s7 |
| human | 324 | 0.06 | 0-0.62 | cluster_s7 |
| human | 325 | 0.06 | 0-0.6 | cluster_s7 |
| human | 326 | 0.06 | 0-0.72 | cluster_s7 |
| human | 327 | 3.28 | 1.9-5.32 | cluster_s7 |
| human | 328 | 0.32 | 0.04-1.19 | cluster_s7 |
| human | 329 | 0.13 | 0-0.77 | cluster_s7 |
| human | 330 | 0.13 | 0-0.95 | cluster_s7 |
| human | 331 | 0.13 | 0-0.86 | cluster_s7 |
| human | 332 | 0.13 | 0-0.92 | cluster_s7 |
| human | 333 | 0.26 | 0.01-1.02 | cluster_s7 |
| human | 334 | 0.38 | 0.05-1.23 | cluster_s7 |
| human | 335 | 0.45 | 0.03-1.3 | cluster_s7 |
| human | 336 | 0.65 | 0.13-1.8 | cluster_s7 |
| human | 337 | 0.13 | 0-0.77 | cluster_s7 |
| human | 338 | 0.13 | 0-0.84 | cluster_s7 |
| human | 339 | 0.39 | 0.05-1.26 | cluster_s7 |
| human | 340 | 0.19 | 0-0.86 | cluster_s7 |
| human | 341 | 0.26 | 0.02-1 | cluster_s7 |
| human | 342 | 0.13 | 0-0.76 | cluster_s7 |
| human | 343 | 0.19 | 0-1.08 | cluster_s7 |
| human | 344 | 0.13 | 0-0.98 | cluster_s7 |
| human | 345 | 0.13 | 0-0.72 | cluster_s7 |
| human | 346 | 0.13 | 0-0.95 | cluster_s7 |
| human | 347 | 0.13 | 0-0.79 | cluster_s7 |
| human | 348 | 0.19 | 0-0.92 | cluster_s7 |
| human | 349 | 0.13 | 0-0.71 | cluster_s7 |
| human | 350 | 0.19 | 0-0.98 | cluster_s7 |
| human | 351 | 0.13 | 0-0.74 | cluster_s7 |
| human | 352 | 0.13 | 0-0.69 | cluster_s7 |
| human | 353 | 0.13 | 0-0.81 | cluster_s7 |
| human | 354 | 0.13 | 0-0.8 | cluster_s7 |
| human | 355 | 0.13 | 0-0.94 | cluster_s7 |
| human | 356 | 0.13 | 0-0.83 | cluster_s7 |
| human | 357 | 0.13 | 0-0.69 | cluster_s7 |
| human | 358 | 0.13 | 0-0.77 | cluster_s7 |
| human | 359 | 0.13 | 0-0.76 | cluster_s7 |
| human | 360 | 0.13 | 0-0.91 | cluster_s7 |
| human | 361 | 0.13 | 0-0.96 | cluster_s7 |
| human | 362 | 0.13 | 0-0.68 | cluster_s7 |
| human | 363 | 0.13 | 0-0.92 | cluster_s7 |
| human | 364 | 0.13 | 0-0.72 | cluster_s7 |
| human | 365 | 0.13 | 0-0.79 | cluster_s7 |
| human | 366 | 0.13 | 0-0.84 | cluster_s7 |
| human | 367 | 0.13 | 0-0.75 | cluster_s7 |
| human | 368 | 0.13 | 0-0.75 | cluster_s7 |
| human | 369 | 0.13 | 0-0.85 | cluster_s7 |
| human | 370 | 0.13 | 0-0.81 | cluster_s7 |
| human | 371 | 0.13 | 0-0.74 | cluster_s7 |
| human | 372 | 0.06 | 0-0.67 | cluster_s7 |
| human | 373 | 0.07 | 0-0.56 | cluster_s7 |
| human | 374 | 0.06 | 0-0.68 | cluster_s7 |
| human | 375 | 0.06 | 0-0.53 | cluster_s7 |
| human | 376 | 0.06 | 0-0.73 | cluster_s7 |
| human | 377 | 0.07 | 0-0.9 | cluster_s7 |
| human | 378 | 0.06 | 0-0.61 | cluster_s7 |
| human | 379 | 0.07 | 0-0.63 | cluster_s7 |
| human | 380 | 0.06 | 0-0.7 | cluster_s7 |
| human | 381 | 0.07 | 0-0.65 | cluster_s7 |
| human | 382 | 0.06 | 0-0.65 | cluster_s7 |
| human | 383 | 0.06 | 0-0.64 | cluster_s7 |
| human | 384 | 0.07 | 0-0.63 | cluster_s7 |
| human | 385 | 0.06 | 0-0.56 | cluster_s7 |
| human | 386 | 0.06 | 0-0.55 | cluster_s7 |
| human | 387 | 0.07 | 0-0.67 | cluster_s7 |
| human | 388 | 0.06 | 0-0.79 | cluster_s7 |
| human | 389 | 0.19 | 0-0.9 | cluster_s7 |
| human | 390 | 0.13 | 0-0.82 | cluster_s7 |
| human | 391 | 0.26 | 0.01-1.12 | cluster_s7 |
| human | 392 | 0.13 | 0-0.75 | cluster_s7 |
| human | 393 | 0.13 | 0-0.86 | cluster_s7 |
| human | 394 | 0.32 | 0.03-1.26 | cluster_s7 |
| human | 395 | 0.13 | 0-0.86 | cluster_s7 |
| human | 396 | 0.19 | 0-0.98 | cluster_s7 |
| human | 397 | 0.13 | 0-0.79 | cluster_s7 |
| human | 398 | 0.13 | 0-0.78 | cluster_s7 |
| human | 399 | 0.13 | 0-0.81 | cluster_s7 |
| human | 400 | 0.13 | 0-0.7 | cluster_s7 |
| human | 401 | 0.19 | 0-0.96 | cluster_s7 |
| human | 402 | 0.13 | 0-0.72 | cluster_s7 |
| human | 403 | 0.13 | 0-0.92 | cluster_s7 |
| human | 404 | 0.13 | 0-0.74 | cluster_s7 |
| human | 405 | 0.13 | 0-0.74 | cluster_s7 |
| human | 406 | 0.13 | 0-0.82 | cluster_s7 |
| human | 407 | 0.13 | 0-0.7 | cluster_s7 |
| human | 408 | 0.13 | 0-0.72 | cluster_s7 |
| human | 409 | 0.13 | 0-0.8 | cluster_s7 |
| human | 410 | 0.19 | 0.01-1.1 | cluster_s7 |
| human | 411 | 0.19 | 0-1.05 | cluster_s7 |
| human | 412 | 0.26 | 0.01-1.09 | cluster_s7 |
| human | 413 | 0.13 | 0-0.96 | cluster_s7 |
| human | 414 | 0.19 | 0-1.07 | cluster_s7 |
| human | 415 | 0.13 | 0-0.81 | cluster_s7 |
| human | 416 | 0.19 | 0-0.94 | cluster_s7 |
| human | 417 | 0.13 | 0-0.77 | cluster_s7 |
| human | 418 | 0.13 | 0-0.84 | cluster_s7 |
| human | 419 | 0.13 | 0-0.72 | cluster_s7 |
| human | 420 | 0.13 | 0-1.1 | cluster_s7 |
| human | 421 | 0.13 | 0-0.71 | cluster_s7 |
| human | 422 | 0.13 | 0-0.87 | cluster_s7 |
| human | 423 | 0.13 | 0-0.8 | cluster_s7 |
| human | 424 | 0.13 | 0-0.73 | cluster_s7 |
| human | 425 | 0.13 | 0-0.68 | cluster_s7 |
| human | 426 | 0.13 | 0-0.79 | cluster_s7 |
| human | 427 | 0.13 | 0-0.74 | cluster_s7 |
| human | 428 | 0.13 | 0-0.73 | cluster_s7 |
| human | 429 | 0.13 | 0-0.74 | cluster_s7 |
| human | 430 | 0.13 | 0-0.86 | cluster_s7 |
| human | 431 | 0.19 | 0.01-0.82 | cluster_s7 |
| human | 432 | 0.13 | 0-0.7 | cluster_s7 |
| human | 433 | 0.06 | 0-0.73 | cluster_s7 |
| human | 434 | 0.06 | 0-0.64 | cluster_s7 |
| human | 435 | 0.06 | 0-0.55 | cluster_s7 |
| human | 436 | 0.07 | 0-0.75 | cluster_s7 |
| human | 437 | 0.07 | 0-0.7 | cluster_s7 |
| human | 438 | 0.06 | 0-0.57 | cluster_s7 |
| human | 439 | 0.06 | 0-0.61 | cluster_s7 |
| human | 440 | 0.06 | 0-0.6 | cluster_s7 |
| human | 441 | 0.06 | 0-0.75 | cluster_s7 |
| human | 442 | 0.06 | 0-0.53 | cluster_s7 |
| human | 443 | 0.06 | 0-0.56 | cluster_s7 |
| human | 444 | 0.06 | 0-0.57 | cluster_s7 |
| human | 445 | 0.06 | 0-0.63 | cluster_s7 |
| human | 446 | 0.06 | 0-0.7 | cluster_s7 |
| human | 447 | 0.07 | 0-0.76 | cluster_s7 |
| human | 448 | 0.06 | 0-0.75 | cluster_s7 |
| human | 449 | 0.06 | 0-0.6 | cluster_s7 |
| human | 450 | 0.06 | 0-0.71 | cluster_s7 |
| human | 451 | 0.32 | 0.03-1.09 | cluster_s7 |
| human | 452 | 0.13 | 0-0.75 | cluster_s7 |
| human | 453 | 0.13 | 0-0.7 | cluster_s7 |
| human | 454 | 0.13 | 0-0.7 | cluster_s7 |
| human | 455 | 0.32 | 0.02-1.29 | cluster_s7 |
| human | 456 | 0.13 | 0-0.78 | cluster_s7 |
| human | 457 | 0.13 | 0-0.7 | cluster_s7 |
| human | 458 | 0.13 | 0-0.92 | cluster_s7 |
| human | 459 | 0.13 | 0-0.82 | cluster_s7 |
| human | 460 | 0.13 | 0-0.88 | cluster_s7 |
| human | 461 | 0.13 | 0-0.78 | cluster_s7 |
| human | 462 | 0.58 | 0.1-1.71 | cluster_s7 |
| human | 463 | 0.13 | 0-0.72 | cluster_s7 |
| human | 464 | 0.13 | 0-0.69 | cluster_s7 |
| human | 465 | 0.13 | 0-0.81 | cluster_s7 |
| human | 466 | 0.13 | 0-0.81 | cluster_s7 |
| human | 467 | 0.13 | 0-0.66 | cluster_s7 |
| human | 468 | 0.13 | 0-0.71 | cluster_s7 |
| human | 469 | 0.13 | 0-1.21 | cluster_s7 |
| human | 470 | 0.13 | 0-0.78 | cluster_s7 |
| human | 471 | 0.13 | 0-0.86 | cluster_s7 |
| human | 472 | 0.13 | 0-0.86 | cluster_s7 |
| human | 473 | 0.13 | 0-0.85 | cluster_s7 |
| human | 474 | 0.13 | 0-0.79 | cluster_s7 |
| human | 475 | 0.13 | 0-0.89 | cluster_s7 |
| human | 476 | 0.13 | 0-0.84 | cluster_s7 |
| human | 477 | 0.19 | 0-1.05 | cluster_s7 |
| human | 478 | 0.13 | 0-0.88 | cluster_s7 |
| human | 479 | 0.13 | 0-0.83 | cluster_s7 |
| human | 480 | 0.13 | 0-0.8 | cluster_s7 |
| human | 481 | 0.13 | 0-0.82 | cluster_s7 |
| human | 482 | 0.13 | 0-0.71 | cluster_s7 |
| human | 483 | 0.13 | 0-0.99 | cluster_s7 |
| human | 484 | 0.13 | 0-0.76 | cluster_s7 |
| human | 485 | 0.13 | 0-0.66 | cluster_s7 |
| human | 486 | 0.13 | 0-0.71 | cluster_s7 |
| human | 487 | 0.13 | 0-0.73 | cluster_s7 |
| human | 488 | 0.13 | 0-0.85 | cluster_s7 |
| human | 489 | 0.13 | 0-0.76 | cluster_s7 |
| human | 490 | 0.13 | 0-0.73 | cluster_s7 |
| human | 491 | 0.19 | 0-0.93 | cluster_s7 |
| human | 492 | 0.13 | 0-0.74 | cluster_s7 |
| human | 493 | 0.26 | 0.01-1.02 | cluster_s7 |
| human | 494 | 0.13 | 0-0.79 | cluster_s7 |
| human | 495 | 0.13 | 0-0.81 | cluster_s7 |
| human | 496 | 0.13 | 0-0.87 | cluster_s7 |
| human | 497 | 0.13 | 0-0.89 | cluster_s7 |
| human | 498 | 0.13 | 0-0.73 | cluster_s7 |
| human | 499 | 0.19 | 0.01-0.93 | cluster_s7 |
| human | 500 | 0.13 | 0-1 | cluster_s7 |
| human | 501 | 0.13 | 0-0.74 | cluster_s7 |
| human | 502 | 0.13 | 0-0.82 | cluster_s7 |
| human | 503 | 0.13 | 0-0.79 | cluster_s7 |
| human | 504 | 0.13 | 0-0.71 | cluster_s7 |
| human | 505 | 0.13 | 0-0.68 | cluster_s7 |
| human | 506 | 0.13 | 0-0.83 | cluster_s7 |
| human | 507 | 0.13 | 0-0.76 | cluster_s7 |
| human | 508 | 0.13 | 0-0.76 | cluster_s7 |
| human | 509 | 0.13 | 0-0.87 | cluster_s7 |
| human | 510 | 0.13 | 0-0.68 | cluster_s7 |
| human | 511 | 0.13 | 0-0.74 | cluster_s7 |
| human | 512 | 0.13 | 0-0.73 | cluster_s7 |
| human | 513 | 0.13 | 0-0.86 | cluster_s7 |
| human | 514 | 0.13 | 0-0.8 | cluster_s7 |
| human | 515 | 0.06 | 0-0.54 | cluster_s7 |
| human | 516 | 0.06 | 0-0.68 | cluster_s7 |
| human | 517 | 0.06 | 0-0.57 | cluster_s7 |
| human | 518 | 0.06 | 0-0.7 | cluster_s7 |
| human | 519 | 0.06 | 0-0.57 | cluster_s7 |
| human | 520 | 0.06 | 0-0.57 | cluster_s7 |
| human | 521 | 0.06 | 0-0.66 | cluster_s7 |
| human | 522 | 0.07 | 0-0.54 | cluster_s7 |
| human | 523 | 0.06 | 0-0.52 | cluster_s7 |
| human | 524 | 0.06 | 0-0.68 | cluster_s7 |
| human | 525 | 0.06 | 0-0.6 | cluster_s7 |
| human | 526 | 0.07 | 0-0.58 | cluster_s7 |
| human | 527 | 0.06 | 0-0.67 | cluster_s7 |
| human | 528 | 0.07 | 0-1.05 | cluster_s7 |
| human | 529 | 0.06 | 0-0.62 | cluster_s7 |
| human | 530 | 0.07 | 0-0.61 | cluster_s7 |
| human | 531 | 0.06 | 0-0.6 | cluster_s7 |
| human | 532 | 0.06 | 0-0.62 | cluster_s7 |
| human | 533 | 0.06 | 0-0.6 | cluster_s7 |
| human | 534 | 0.06 | 0-0.55 | cluster_s7 |
| human | 535 | 0.06 | 0-0.59 | cluster_s7 |
| human | 536 | 0.06 | 0-0.54 | cluster_s7 |
| human | 537 | 0.06 | 0-0.68 | cluster_s7 |
| human | 538 | 0.07 | 0-0.62 | cluster_s7 |
| human | 539 | 0.06 | 0-0.66 | cluster_s7 |
| human | 540 | 0.06 | 0-0.6 | cluster_s7 |
| human | 541 | 0.06 | 0-0.72 | cluster_s7 |
| human | 542 | 0.06 | 0-0.58 | cluster_s7 |
| human | 543 | 0.07 | 0-0.75 | cluster_s7 |
| human | 544 | 0.13 | 0-0.76 | cluster_s7 |
| human | 545 | 0.13 | 0-0.74 | cluster_s7 |
| human | 546 | 0.13 | 0-0.83 | cluster_s7 |
| human | 547 | 0.64 | 0.13-1.74 | cluster_s7 |
| human | 548 | 0.33 | 0.03-1.22 | cluster_s7 |
| human | 549 | 0.19 | 0-1.18 | cluster_s7 |
| human | 550 | 0.13 | 0-0.8 | cluster_s7 |
| human | 551 | 0.13 | 0-0.77 | cluster_s7 |
| human | 552 | 0.13 | 0-1.02 | cluster_s7 |
| human | 553 | 0.13 | 0-0.78 | cluster_s7 |
| human | 554 | 0.13 | 0-0.79 | cluster_s7 |
| human | 555 | 0.13 | 0-0.83 | cluster_s7 |
| human | 556 | 0.13 | 0-0.72 | cluster_s7 |
| human | 557 | 0.13 | 0-0.76 | cluster_s7 |
| human | 558 | 0.13 | 0-0.74 | cluster_s7 |
| human | 559 | 0.13 | 0-0.68 | cluster_s7 |
| human | 560 | 0.13 | 0-0.81 | cluster_s7 |
| human | 561 | 0.13 | 0-0.8 | cluster_s7 |
| human | 562 | 0.13 | 0-0.79 | cluster_s7 |
| human | 563 | 0.13 | 0-0.75 | cluster_s7 |
| human | 564 | 0.13 | 0-1 | cluster_s7 |
| human | 565 | 0.19 | 0-0.81 | cluster_s7 |
| human | 566 | 0.13 | 0-0.82 | cluster_s7 |
| human | 567 | 0.13 | 0-0.76 | cluster_s7 |
| human | 568 | 0.13 | 0-0.84 | cluster_s7 |
| human | 569 | 0.13 | 0-0.82 | cluster_s7 |
| human | 570 | 0.13 | 0-0.79 | cluster_s7 |
| human | 571 | 0.26 | 0.01-1.19 | cluster_s7 |
| human | 572 | 0.13 | 0-0.79 | cluster_s7 |
| human | 573 | 0.13 | 0-0.98 | cluster_s7 |
| human | 574 | 0.13 | 0-0.72 | cluster_s7 |
| human | 575 | 0.13 | 0-0.73 | cluster_s7 |
| human | 576 | 0.13 | 0-0.81 | cluster_s7 |
| human | 577 | 0.13 | 0-0.79 | cluster_s7 |
| human | 578 | 0.13 | 0-0.73 | cluster_s7 |
| human | 579 | 0.13 | 0-0.81 | cluster_s7 |
| human | 580 | 0.13 | 0-0.95 | cluster_s7 |
| human | 581 | 0.13 | 0-0.71 | cluster_s7 |
| human | 582 | 0.13 | 0-0.76 | cluster_s7 |
| human | 583 | 0.13 | 0-0.75 | cluster_s7 |
| human | 584 | 0.13 | 0-0.74 | cluster_s7 |
| human | 585 | 0.13 | 0-0.85 | cluster_s7 |
| human | 586 | 0.13 | 0-0.68 | cluster_s7 |
| human | 587 | 0.13 | 0-0.8 | cluster_s7 |
| human | 588 | 0.13 | 0-0.68 | cluster_s7 |
| human | 589 | 0.13 | 0-0.75 | cluster_s7 |
| human | 590 | 0.13 | 0-0.74 | cluster_s7 |
| human | 591 | 0.13 | 0-0.73 | cluster_s7 |
| human | 592 | 0.13 | 0-0.8 | cluster_s7 |
| human | 593 | 0.13 | 0-0.75 | cluster_s7 |
| human | 594 | 0.13 | 0-0.8 | cluster_s7 |
| human | 595 | 0.13 | 0-0.78 | cluster_s7 |
| human | 596 | 0.13 | 0-0.7 | cluster_s7 |
| human | 597 | 0.13 | 0-0.7 | cluster_s7 |
| human | 598 | 0.13 | 0-0.72 | cluster_s7 |
| human | 599 | 0.13 | 0-0.81 | cluster_s7 |
| human | 600 | 0.13 | 0-0.68 | cluster_s7 |
| human | 601 | 0.13 | 0-0.69 | cluster_s7 |
| human | 602 | 0.13 | 0-0.88 | cluster_s7 |
| human | 603 | 0.13 | 0-0.77 | cluster_s7 |
| human | 604 | 0.13 | 0-0.73 | cluster_s7 |
| human | 605 | 0.13 | 0-0.71 | cluster_s7 |
| human | 606 | 0.13 | 0-0.86 | cluster_s7 |
| human | 607 | 0.13 | 0-0.73 | cluster_s7 |
| human | 608 | 0.13 | 0-0.79 | cluster_s7 |
| human | 609 | 0.13 | 0-0.79 | cluster_s7 |
| human | 610 | 0.13 | 0-0.78 | cluster_s7 |
| human | 611 | 0.77 | 0.22-1.91 | cluster_s7 |
| human | 612 | 0.13 | 0-0.99 | cluster_s7 |
| human | 613 | 0.77 | 0.22-1.96 | cluster_s7 |
| human | 614 | 0.13 | 0-0.79 | cluster_s7 |
| human | 615 | 0.06 | 0-0.72 | cluster_s7 |
| human | 616 | 0.06 | 0-0.55 | cluster_s7 |
| human | 617 | 0.06 | 0-0.53 | cluster_s7 |
| human | 618 | 0.06 | 0-0.56 | cluster_s7 |
| human | 619 | 0.06 | 0-0.62 | cluster_s7 |
| human | 620 | 0.06 | 0-0.72 | cluster_s7 |
| human | 621 | 0.06 | 0-0.59 | cluster_s7 |
| human | 622 | 0.06 | 0-0.63 | cluster_s7 |
| human | 623 | 0.06 | 0-0.55 | cluster_s7 |
| human | 624 | 0.07 | 0-0.76 | cluster_s7 |
| human | 625 | 0.06 | 0-0.61 | cluster_s7 |
| human | 626 | 0.06 | 0-0.54 | cluster_s7 |
| human | 627 | 0.06 | 0-0.62 | cluster_s7 |
| human | 628 | 0.06 | 0-0.73 | cluster_s7 |
| human | 629 | 0.06 | 0-0.54 | cluster_s7 |
| human | 630 | 0.06 | 0-0.6 | cluster_s7 |
| human | 631 | 0.06 | 0-0.56 | cluster_s7 |
| human | 632 | 0.06 | 0-0.56 | cluster_s7 |
| human | 633 | 0.13 | 0-0.75 | cluster_s7 |
| human | 634 | 0.06 | 0-0.66 | cluster_s7 |
| human | 635 | 0.06 | 0-0.72 | cluster_s7 |
| human | 636 | 0.06 | 0-0.62 | cluster_s7 |
| human | 637 | 0.19 | 0.01-1.01 | cluster_s7 |
| human | 638 | 0.19 | 0-1.35 | cluster_s7 |
| human | 639 | 0.13 | 0-0.79 | cluster_s7 |
| human | 640 | 0.39 | 0.04-1.33 | cluster_s7 |
| human | 641 | 0.13 | 0-0.68 | cluster_s7 |
| human | 642 | 0.13 | 0-0.72 | cluster_s7 |
| human | 643 | 0.13 | 0-0.98 | cluster_s7 |
| human | 644 | 0.26 | 0.01-1.07 | cluster_s7 |
| human | 645 | 0.13 | 0-0.7 | cluster_s7 |
| human | 646 | 0.13 | 0-0.79 | cluster_s7 |
| human | 647 | 0.71 | 0.14-1.95 | cluster_s7 |
| human | 648 | 0.13 | 0-0.72 | cluster_s7 |
| human | 649 | 0.13 | 0-0.71 | cluster_s7 |
| human | 650 | 0.13 | 0-0.71 | cluster_s7 |
| human | 651 | 0.13 | 0-0.84 | cluster_s7 |
| human | 652 | 0.39 | 0.02-1.41 | cluster_s7 |
| human | 653 | 0.13 | 0-0.8 | cluster_s7 |
| human | 654 | 0.13 | 0-0.87 | cluster_s7 |
| human | 655 | 0.13 | 0-0.79 | cluster_s7 |
| human | 656 | 0.13 | 0-0.68 | cluster_s7 |
| human | 657 | 0.13 | 0-0.75 | cluster_s7 |
| human | 658 | 0.45 | 0.08-1.39 | cluster_s7 |
| human | 659 | 0.19 | 0-0.95 | cluster_s7 |
| human | 660 | 0.13 | 0-0.79 | cluster_s7 |
| human | 661 | 0.13 | 0-0.91 | cluster_s7 |
| human | 662 | 0.13 | 0-0.84 | cluster_s7 |
| human | 663 | 0.13 | 0-0.78 | cluster_s7 |
| human | 664 | 0.13 | 0-0.8 | cluster_s7 |
| human | 665 | 0.13 | 0-0.76 | cluster_s7 |
| human | 666 | 0.13 | 0-0.88 | cluster_s7 |
| human | 667 | 0.13 | 0-0.81 | cluster_s7 |
| human | 668 | 0.13 | 0-0.74 | cluster_s7 |
| human | 669 | 0.13 | 0-0.87 | cluster_s7 |
| human | 670 | 0.13 | 0-0.82 | cluster_s7 |
| human | 671 | 0.13 | 0-0.72 | cluster_s7 |
| human | 672 | 0.13 | 0-0.95 | cluster_s7 |
| human | 673 | 0.13 | 0-0.68 | cluster_s7 |
| human | 674 | 0.19 | 0-0.96 | cluster_s7 |
| human | 675 | 0.07 | 0-0.52 | cluster_s7 |
| human | 676 | 0.06 | 0-0.54 | cluster_s7 |
| human | 677 | 0.06 | 0-0.6 | cluster_s7 |
| human | 678 | 0.13 | 0-0.89 | cluster_s7 |
| human | 679 | 0.06 | 0-0.6 | cluster_s7 |
| human | 680 | 0.06 | 0-0.71 | cluster_s7 |
| human | 681 | 0.06 | 0-0.69 | cluster_s7 |
| human | 682 | 0.06 | 0-0.57 | cluster_s7 |
| human | 683 | 0.07 | 0-0.62 | cluster_s7 |
| human | 684 | 0.07 | 0-0.56 | cluster_s7 |
| human | 685 | 0.06 | 0-0.66 | cluster_s7 |
| human | 686 | 0.06 | 0-0.58 | cluster_s7 |
| human | 687 | 0.06 | 0-0.57 | cluster_s7 |
| human | 688 | 0.07 | 0-0.74 | cluster_s7 |
| human | 689 | 0.06 | 0-0.52 | cluster_s7 |
| human | 690 | 0.19 | 0.01-1.01 | cluster_s7 |
| human | 691 | 0.19 | 0-0.89 | cluster_s7 |
| human | 692 | 0.13 | 0-0.71 | cluster_s7 |
| human | 693 | 0.13 | 0-0.94 | cluster_s7 |
| human | 694 | 0.13 | 0-0.7 | cluster_s7 |
| human | 695 | 0.13 | 0-0.79 | cluster_s7 |
| human | 696 | 0.13 | 0-0.78 | cluster_s7 |
| human | 697 | 0.13 | 0-0.78 | cluster_s7 |
| human | 698 | 0.13 | 0-0.9 | cluster_s7 |
| human | 699 | 0.13 | 0-0.88 | cluster_s7 |
| human | 700 | 0.13 | 0-0.71 | cluster_s7 |
| human | 701 | 0.13 | 0-0.96 | cluster_s7 |
| human | 702 | 0.13 | 0-0.79 | cluster_s7 |
| human | 703 | 0.13 | 0-0.72 | cluster_s7 |
| human | 704 | 0.13 | 0-0.84 | cluster_s7 |
| human | 705 | 0.13 | 0-0.72 | cluster_s7 |
| human | 706 | 0.13 | 0-0.81 | cluster_s7 |
| human | 707 | 0.13 | 0-0.76 | cluster_s7 |
| human | 708 | 0.06 | 0-0.76 | cluster_s7 |
| human | 709 | 0.06 | 0-0.58 | cluster_s7 |
| human | 710 | 0.06 | 0-0.58 | cluster_s7 |
| human | 711 | 0.13 | 0-0.88 | cluster_s7 |
| human | 712 | 0.13 | 0-0.87 | cluster_s7 |
| human | 713 | 0.13 | 0-0.75 | cluster_s7 |
| human | 714 | 0.13 | 0-0.99 | cluster_s7 |
| human | 715 | 0.19 | 0.01-0.82 | cluster_s7 |
| human | 716 | 0.06 | 0-0.6 | cluster_s7 |
| human | 717 | 0.13 | 0-0.74 | cluster_s7 |
| human | 718 | 0.13 | 0-0.82 | cluster_s7 |
| human | 719 | 0.13 | 0-0.96 | cluster_s7 |
| human | 720 | 0.13 | 0-0.7 | cluster_s7 |
| human | 721 | 0.13 | 0-0.92 | cluster_s7 |
| human | 722 | 0.13 | 0-0.8 | cluster_s7 |
| human | 723 | 0.13 | 0-0.86 | cluster_s7 |
| human | 724 | 0.13 | 0-0.76 | cluster_s7 |
| human | 725 | 0.13 | 0-0.83 | cluster_s7 |
| human | 726 | 0.13 | 0-0.73 | cluster_s7 |
| human | 727 | 0.13 | 0-0.78 | cluster_s7 |
| human | 728 | 0.13 | 0-0.83 | cluster_s7 |
| human | 729 | 0.13 | 0-1.04 | cluster_s7 |
| human | 730 | 0.06 | 0-0.59 | cluster_s7 |
| human | 731 | 0.06 | 0-0.67 | cluster_s7 |
| human | 732 | 0.13 | 0-0.82 | cluster_s7 |
| human | 733 | 0.06 | 0-0.67 | cluster_s7 |
| human | 734 | 0.06 | 0-0.61 | cluster_s7 |
| human | 735 | 0.06 | 0-0.58 | cluster_s7 |
| human | 736 | 0.06 | 0-0.67 | cluster_s7 |
| human | 737 | 0.06 | 0-0.6 | cluster_s7 |
| human | 738 | 0.13 | 0-0.76 | cluster_s7 |
| human | 739 | 0.06 | 0-0.68 | cluster_s7 |
| human | 740 | 0.06 | 0-0.52 | cluster_s7 |
| human | 741 | 0.13 | 0-0.97 | cluster_s7 |
| human | 742 | 0.13 | 0-0.79 | cluster_s7 |
| human | 743 | 0.13 | 0-0.94 | cluster_s7 |
| human | 744 | 0.13 | 0-0.86 | cluster_s7 |
| human | 745 | 0.13 | 0-0.84 | cluster_s7 |
| human | 746 | 0.13 | 0-0.74 | cluster_s7 |
| human | 747 | 0.06 | 0-0.7 | cluster_s7 |
| human | 748 | 0.06 | 0-0.59 | cluster_s7 |
| human | 749 | 0.13 | 0-0.7 | cluster_s7 |
| human | 750 | 0.13 | 0-0.79 | cluster_s7 |
| human | 751 | 0.13 | 0-0.94 | cluster_s7 |
| human | 752 | 0.07 | 0-0.68 | cluster_s7 |
| human | 753 | 0.13 | 0-0.62 | cluster_s7 |
| human | 754 | 0.13 | 0-0.77 | cluster_s7 |
| human | 755 | 0.07 | 0-0.62 | cluster_s7 |
| human | 756 | 0.07 | 0-0.58 | cluster_s7 |
| human | 757 | 0.13 | 0-0.73 | cluster_s7 |
| human | 758 | 0.13 | 0-0.74 | cluster_s7 |
| human | 759 | 0.13 | 0-0.75 | cluster_s7 |
| human | 760 | 0.26 | 0.01-1.06 | cluster_s7 |
| human | 761 | 0.13 | 0-1.01 | cluster_s7 |
| human | 762 | 0.19 | 0.01-0.91 | cluster_s7 |
| human | 763 | 0.13 | 0-0.72 | cluster_s7 |
| human | 764 | 0.13 | 0-0.77 | cluster_s7 |
| human | 765 | 0.13 | 0-0.83 | cluster_s7 |
| human | 766 | 0.13 | 0-0.72 | cluster_s7 |
| human | 767 | 0.19 | 0-0.94 | cluster_s7 |
| human | 768 | 0.13 | 0-0.82 | cluster_s7 |
| human | 769 | 0.13 | 0-0.83 | cluster_s7 |
| human | 770 | 0.13 | 0-0.83 | cluster_s7 |
| human | 771 | 0.13 | 0-0.76 | cluster_s7 |
| human | 772 | 0.13 | 0-0.72 | cluster_s7 |
| human | 773 | 0.13 | 0-0.72 | cluster_s7 |
| human | 774 | 0.13 | 0-0.82 | cluster_s7 |
| human | 775 | 0.06 | 0-0.56 | cluster_s7 |
| human | 776 | 0.06 | 0-0.52 | cluster_s7 |
| human | 777 | 0.07 | 0-0.62 | cluster_s7 |
| human | 778 | 0.06 | 0-0.6 | cluster_s7 |
| human | 779 | 0.07 | 0-0.62 | cluster_s7 |
| human | 780 | 0.06 | 0-0.58 | cluster_s7 |
| human | 781 | 0.06 | 0-0.6 | cluster_s7 |
| human | 782 | 0.06 | 0-0.64 | cluster_s7 |
| human | 783 | 0.06 | 0-0.56 | cluster_s7 |
| human | 784 | 0.06 | 0-0.55 | cluster_s7 |
| human | 785 | 0.06 | 0-0.62 | cluster_s7 |
| human | 786 | 0.06 | 0-0.51 | cluster_s7 |
| human | 787 | 0.06 | 0-0.98 | cluster_s7 |
| human | 788 | 0.06 | 0-0.59 | cluster_s7 |
| human | 789 | 0.06 | 0-0.65 | cluster_s7 |
| human | 790 | 0.07 | 0-0.66 | cluster_s7 |
| human | 791 | 0.06 | 0-0.6 | cluster_s7 |
| human | 792 | 0.06 | 0-0.53 | cluster_s7 |
| human | 793 | 0.06 | 0-0.62 | cluster_s7 |
| human | 794 | 0.06 | 0-0.57 | cluster_s7 |
| human | 795 | 0.06 | 0-0.56 | cluster_s7 |
| human | 796 | 0.06 | 0-0.62 | cluster_s7 |

**Supplementary Table 6**: Multi-year clusters.

| cluster | 2010 | 2011 | 2012 | 2013 | 2014 | 2015 | 2016 | 2017 | 2018 | 2019 | 2020 |
| --- | --- | --- | --- | --- | --- | --- | --- | --- | --- | --- | --- |
| cluster2 | 0 | 0 | 0 | 0 | 0 | 0 | 1 | 17 | 15 | 8 | 12 |
| cluster3 | 0 | 0 | 0 | 0 | 0 | 0 | 0 | 2 | 4 | 1 | 1 |
| cluster12 | 0 | 0 | 0 | 1 | 0 | 0 | 0 | 0 | 0 | 1 | 0 |
| cluster14 | 0 | 0 | 0 | 0 | 1 | 0 | 0 | 0 | 0 | 0 | 1 |
| cluster16 | 0 | 0 | 1 | 0 | 3 | 1 | 0 | 0 | 0 | 0 | 0 |
| cluster20 | 0 | 0 | 0 | 0 | 0 | 1 | 10 | 4 | 2 | 0 | 2 |
| cluster23 | 0 | 0 | 0 | 0 | 0 | 0 | 0 | 2 | 0 | 2 | 0 |
| cluster25 | 0 | 0 | 0 | 0 | 0 | 0 | 1 | 0 | 1 | 0 | 0 |
| cluster29 | 0 | 0 | 0 | 0 | 0 | 2 | 0 | 0 | 0 | 3 | 2 |
| cluster31 | 0 | 0 | 0 | 0 | 0 | 0 | 1 | 0 | 0 | 1 | 0 |
| cluster40 | 0 | 0 | 0 | 0 | 0 | 0 | 2 | 1 | 0 | 1 | 0 |
| cluster42 | 1 | 1 | 0 | 0 | 0 | 0 | 4 | 1 | 3 | 2 | 0 |
| cluster45 | 0 | 0 | 0 | 0 | 0 | 0 | 1 | 1 | 2 | 0 | 0 |
| cluster46 | 0 | 0 | 0 | 0 | 1 | 1 | 1 | 3 | 1 | 0 | 1 |
| cluster50 | 0 | 0 | 0 | 0 | 0 | 0 | 0 | 1 | 1 | 0 | 0 |
| cluster53 | 0 | 0 | 0 | 0 | 0 | 0 | 0 | 2 | 0 | 0 | 7 |
| cluster61 | 0 | 0 | 0 | 0 | 0 | 0 | 0 | 1 | 0 | 1 | 0 |
| cluster66 | 0 | 0 | 0 | 0 | 0 | 0 | 0 | 0 | 1 | 1 | 0 |
| cluster71 | 0 | 0 | 0 | 0 | 0 | 0 | 1 | 1 | 2 | 1 | 1 |
| cluster72 | 0 | 0 | 0 | 0 | 0 | 0 | 1 | 0 | 1 | 0 | 1 |
| cluster74 | 0 | 0 | 0 | 0 | 0 | 0 | 0 | 2 | 2 | 1 | 2 |
| cluster81 | 0 | 0 | 0 | 0 | 0 | 0 | 0 | 0 | 0 | 3 | 3 |
| cluster86 | 0 | 0 | 0 | 0 | 0 | 0 | 0 | 0 | 0 | 2 | 2 |
| cluster90 | 0 | 0 | 0 | 0 | 0 | 0 | 0 | 0 | 0 | 2 | 3 |
| cluster106 | 2 | 1 | 0 | 2 | 0 | 0 | 0 | 0 | 0 | 0 | 0 |
| cluster107 | 0 | 0 | 1 | 0 | 0 | 1 | 1 | 1 | 0 | 1 | 1 |
| cluster109 | 0 | 0 | 1 | 0 | 1 | 2 | 3 | 1 | 0 | 1 | 0 |
| cluster110 | 0 | 0 | 1 | 0 | 0 | 0 | 0 | 0 | 0 | 0 | 1 |
| cluster112 | 0 | 0 | 0 | 0 | 2 | 1 | 3 | 6 | 2 | 2 | 0 |
| cluster126 | 0 | 0 | 0 | 0 | 0 | 0 | 1 | 0 | 1 | 4 | 1 |
| cluster136 | 0 | 0 | 1 | 0 | 1 | 0 | 0 | 0 | 0 | 0 | 1 |
| cluster139 | 0 | 0 | 0 | 0 | 0 | 1 | 1 | 0 | 4 | 3 | 0 |
| cluster145 | 0 | 0 | 0 | 0 | 0 | 0 | 6 | 14 | 6 | 0 | 1 |
| cluster147 | 0 | 0 | 0 | 0 | 0 | 0 | 1 | 1 | 0 | 1 | 1 |
| cluster150 | 0 | 0 | 0 | 0 | 0 | 0 | 1 | 0 | 0 | 1 | 0 |
| cluster156 | 0 | 0 | 0 | 0 | 0 | 0 | 2 | 2 | 1 | 0 | 0 |
| cluster159 | 0 | 0 | 0 | 0 | 0 | 0 | 0 | 2 | 0 | 0 | 1 |
| cluster163 | 0 | 0 | 0 | 0 | 0 | 1 | 0 | 1 | 1 | 0 | 0 |
| cluster173 | 0 | 0 | 0 | 0 | 0 | 0 | 0 | 2 | 1 | 1 | 1 |
| cluster174 | 0 | 0 | 0 | 0 | 0 | 0 | 0 | 0 | 1 | 1 | 0 |
| cluster178 | 0 | 0 | 0 | 0 | 0 | 0 | 1 | 1 | 1 | 0 | 0 |
| cluster184 | 0 | 0 | 1 | 0 | 0 | 0 | 0 | 0 | 0 | 4 | 0 |
| cluster185 | 0 | 0 | 0 | 0 | 0 | 0 | 0 | 0 | 0 | 2 | 2 |
| cluster190 | 0 | 0 | 0 | 0 | 0 | 0 | 0 | 1 | 0 | 2 | 0 |
| cluster203 | 0 | 0 | 0 | 0 | 1 | 0 | 0 | 1 | 0 | 0 | 0 |
| cluster208 | 0 | 0 | 0 | 0 | 0 | 0 | 2 | 11 | 2 | 1 | 0 |
| cluster209 | 0 | 0 | 1 | 0 | 0 | 0 | 1 | 0 | 0 | 0 | 0 |
| cluster213 | 0 | 0 | 0 | 0 | 0 | 5 | 0 | 2 | 10 | 6 | 2 |
| cluster215 | 0 | 0 | 0 | 0 | 0 | 0 | 0 | 2 | 1 | 1 | 0 |
| cluster218 | 0 | 0 | 0 | 0 | 0 | 0 | 4 | 0 | 1 | 0 | 0 |
| cluster222 | 0 | 0 | 0 | 0 | 0 | 0 | 1 | 2 | 0 | 2 | 1 |
| cluster228 | 0 | 0 | 0 | 0 | 0 | 0 | 0 | 1 | 0 | 0 | 1 |
| cluster239 | 0 | 0 | 0 | 0 | 0 | 0 | 0 | 0 | 2 | 0 | 1 |
| cluster253 | 0 | 0 | 1 | 0 | 0 | 0 | 0 | 5 | 2 | 4 | 0 |
| cluster259 | 0 | 0 | 0 | 2 | 0 | 0 | 1 | 2 | 0 | 1 | 0 |
| cluster264 | 0 | 0 | 0 | 0 | 2 | 0 | 0 | 0 | 0 | 1 | 0 |
| cluster271 | 0 | 0 | 0 | 1 | 0 | 1 | 0 | 0 | 0 | 0 | 1 |
| cluster274 | 0 | 0 | 0 | 0 | 0 | 0 | 8 | 6 | 1 | 6 | 0 |
| cluster293 | 0 | 0 | 1 | 0 | 0 | 1 | 2 | 1 | 1 | 2 | 2 |
| cluster294 | 0 | 0 | 0 | 2 | 5 | 3 | 5 | 1 | 0 | 0 | 0 |
| cluster299 | 0 | 0 | 0 | 0 | 0 | 0 | 1 | 1 | 1 | 0 | 0 |
| cluster306 | 0 | 0 | 0 | 0 | 0 | 0 | 0 | 3 | 1 | 0 | 1 |
| cluster327 | 11 | 4 | 0 | 3 | 6 | 2 | 0 | 4 | 10 | 13 | 0 |
| cluster333 | 1 | 1 | 1 | 0 | 0 | 0 | 0 | 0 | 0 | 0 | 0 |
| cluster334 | 1 | 1 | 0 | 1 | 1 | 0 | 1 | 0 | 0 | 0 | 0 |
| cluster335 | 3 | 0 | 0 | 0 | 0 | 0 | 0 | 0 | 0 | 1 | 2 |
| cluster336 | 1 | 0 | 1 | 0 | 1 | 0 | 0 | 3 | 1 | 2 | 2 |
| cluster339 | 0 | 3 | 0 | 0 | 0 | 0 | 0 | 1 | 1 | 0 | 0 |
| cluster341 | 0 | 1 | 0 | 0 | 0 | 0 | 0 | 0 | 2 | 0 | 0 |
| cluster342 | 0 | 0 | 1 | 0 | 0 | 0 | 1 | 0 | 0 | 0 | 0 |
| cluster343 | 0 | 0 | 1 | 0 | 0 | 0 | 0 | 0 | 0 | 1 | 0 |
| cluster348 | 0 | 0 | 0 | 0 | 0 | 0 | 1 | 0 | 0 | 1 | 0 |
| cluster350 | 0 | 0 | 0 | 0 | 0 | 0 | 2 | 1 | 0 | 0 | 0 |
| cluster373 | 0 | 0 | 0 | 0 | 0 | 0 | 3 | 0 | 1 | 0 | 0 |
| cluster374 | 0 | 0 | 0 | 0 | 0 | 0 | 1 | 0 | 1 | 1 | 0 |
| cluster380 | 0 | 0 | 0 | 0 | 0 | 0 | 0 | 0 | 1 | 1 | 0 |
| cluster389 | 1 | 0 | 0 | 1 | 0 | 0 | 0 | 0 | 0 | 0 | 0 |
| cluster391 | 0 | 1 | 0 | 0 | 0 | 1 | 0 | 0 | 1 | 0 | 0 |
| cluster394 | 0 | 0 | 1 | 0 | 0 | 0 | 0 | 3 | 0 | 0 | 0 |
| cluster412 | 0 | 0 | 0 | 0 | 2 | 0 | 1 | 0 | 0 | 0 | 0 |
| cluster416 | 0 | 0 | 0 | 0 | 0 | 0 | 5 | 0 | 1 | 1 | 1 |
| cluster425 | 0 | 0 | 0 | 0 | 0 | 1 | 0 | 0 | 1 | 0 | 0 |
| cluster451 | 1 | 0 | 1 | 0 | 0 | 0 | 1 | 0 | 1 | 0 | 0 |
| cluster462 | 0 | 1 | 1 | 1 | 1 | 1 | 2 | 1 | 0 | 0 | 0 |
| cluster477 | 0 | 0 | 0 | 0 | 0 | 1 | 0 | 0 | 0 | 0 | 1 |
| cluster493 | 0 | 0 | 0 | 0 | 0 | 1 | 0 | 2 | 0 | 0 | 0 |
| cluster499 | 0 | 0 | 0 | 0 | 0 | 0 | 0 | 1 | 0 | 1 | 0 |
| cluster516 | 0 | 0 | 0 | 0 | 0 | 2 | 0 | 1 | 0 | 1 | 1 |
| cluster526 | 0 | 0 | 0 | 0 | 0 | 0 | 0 | 3 | 0 | 1 | 0 |
| cluster527 | 0 | 0 | 0 | 0 | 0 | 0 | 0 | 1 | 1 | 0 | 0 |
| cluster530 | 0 | 0 | 0 | 0 | 0 | 0 | 0 | 2 | 6 | 0 | 3 |
| cluster547 | 1 | 3 | 3 | 1 | 0 | 0 | 0 | 1 | 0 | 0 | 0 |
| cluster549 | 0 | 1 | 1 | 0 | 0 | 0 | 0 | 0 | 0 | 0 | 0 |
| cluster571 | 0 | 0 | 0 | 2 | 0 | 0 | 0 | 0 | 1 | 0 | 0 |
| cluster611 | 0 | 0 | 0 | 0 | 0 | 0 | 0 | 1 | 0 | 2 | 9 |
| cluster613 | 0 | 0 | 0 | 0 | 0 | 0 | 0 | 0 | 0 | 2 | 26 |
| cluster615 | 0 | 0 | 0 | 0 | 0 | 2 | 0 | 1 | 0 | 0 | 0 |
| cluster623 | 0 | 0 | 0 | 0 | 0 | 0 | 0 | 1 | 3 | 0 | 3 |
| cluster633 | 0 | 0 | 1 | 0 | 0 | 0 | 0 | 0 | 0 | 2 | 0 |
| cluster638 | 2 | 0 | 0 | 0 | 0 | 0 | 0 | 0 | 0 | 0 | 0 |
| cluster640 | 1 | 0 | 0 | 0 | 1 | 0 | 1 | 0 | 2 | 0 | 0 |
| cluster644 | 0 | 2 | 1 | 0 | 0 | 0 | 0 | 0 | 0 | 0 | 0 |
| cluster647 | 0 | 0 | 1 | 0 | 1 | 1 | 0 | 2 | 4 | 1 | 0 |
| cluster652 | 0 | 0 | 0 | 0 | 1 | 0 | 0 | 0 | 0 | 3 | 1 |
| cluster658 | 0 | 3 | 0 | 0 | 0 | 0 | 0 | 3 | 0 | 0 | 0 |
| cluster674 | 0 | 0 | 0 | 0 | 1 | 0 | 0 | 0 | 0 | 0 | 1 |
| cluster678 | 1 | 0 | 0 | 0 | 0 | 0 | 1 | 0 | 0 | 0 | 0 |
| cluster715 | 0 | 0 | 0 | 0 | 0 | 0 | 0 | 0 | 0 | 1 | 1 |
| cluster760 | 1 | 0 | 0 | 0 | 0 | 0 | 1 | 1 | 0 | 0 | 0 |
| cluster762 | 0 | 0 | 1 | 1 | 0 | 0 | 0 | 0 | 1 | 0 | 0 |
| cluster780 | 0 | 0 | 0 | 0 | 0 | 0 | 0 | 0 | 1 | 1 | 0 |

**Supplementary Table 7**: Potential food sources for the sporadic human isolates.

| food_category | total | d<=20 | 20<d<=50 | 50<d<=100 | d>100 |
| --- | --- | --- | --- | --- | --- |
| chicken | 137 | 31 | 80 | 14 | 12 |
| cattle | 162 | 38 | 90 | 13 | 21 |
| fish/shellfish | 154 | 92 | 56 | 5 | 1 |
| small ruminants | 81 | 22 | 45 | 4 | 10 |
| turkey | 3 | 3 | 0 | 0 | 0 |
| spices/herbs/vegetables | 8 | 0 | 4 | 4 | 0 |
| swine | 32 | 10 | 22 | 0 | 0 |
| game | 1 | 1 | 0 | 0 | 0 |
| amphibia | 5 | 1 | 4 | 0 | 0 |
| total | 583 | 198 | 301 | 40 | 44 |

**Supplementary Table 8**: Results of the multinomial logistic regression for association of genotypes with epidemiological variables.

| explanatory_variable | response_variable | p_value | exponent_value | genotype |
| --- | --- | --- | --- | --- |
| CC14 | host_age_groups[65,80) | <0.01 | 6.97 | CC |
| CC14 | host_age_groups[80,100) | =0.029 | 3.98 | CC |
| CC19 | host_age_groups[80,100) | =0.048 | 8.89 | CC |
| CC6 | host_age_groups[65,80) | =0.013 | 2.34 | CC |
| Lineage1 | antacids1 | =0.04 | 1.44 | Lineage |
| ST14 | host_sexV | <0.01 | 18.06 | ST |
| ST399 | host_age_groups[65,80) | =0.031 | 5.79 | ST |
| ST6 | host_age_groups[65,80) | =0.026 | 2.18 | ST |

**Supplementary Table 9**: Accession numbers for the sequences used in this study.

| sample | study | source |
| --- | --- | --- |
| ERS2407350 | PRJEB26058 | human |
| ERS2407352 | PRJEB26058 | human |
| ERS2407351 | PRJEB26058 | human |
| ERS2407356 | PRJEB26058 | human |
| ERS2407354 | PRJEB26058 | human |
| ERS2407355 | PRJEB26058 | human |
| ERS2407357 | PRJEB26058 | human |
| ERS2407358 | PRJEB26058 | human |
| ERS2407360 | PRJEB26058 | human |
| ERS2407359 | PRJEB26058 | human |
| ERS2407363 | PRJEB26058 | human |
| ERS2407353 | PRJEB26058 | human |
| ERS2407362 | PRJEB26058 | human |
| ERS2407364 | PRJEB26058 | human |
| ERS2407365 | PRJEB26058 | human |
| ERS2407367 | PRJEB26058 | human |
| ERS2407368 | PRJEB26058 | human |
| ERS2407371 | PRJEB26058 | human |
| ERS2407372 | PRJEB26058 | human |
| ERS2407374 | PRJEB26058 | human |
| ERS2407373 | PRJEB26058 | human |
| ERS2407375 | PRJEB26058 | human |
| ERS2407376 | PRJEB26058 | human |
| ERS2407377 | PRJEB26058 | human |
| ERS2407378 | PRJEB26058 | human |
| ERS2407379 | PRJEB26058 | human |
| ERS2407382 | PRJEB26058 | human |
| ERS2407380 | PRJEB26058 | human |
| ERS2407383 | PRJEB26058 | human |
| ERS2407386 | PRJEB26058 | human |
| ERS2407387 | PRJEB26058 | human |
| ERS2407384 | PRJEB26058 | human |
| ERS2407385 | PRJEB26058 | human |
| ERS2407388 | PRJEB26058 | human |
| ERS2407389 | PRJEB26058 | human |
| ERS2407393 | PRJEB26058 | human |
| ERS2407390 | PRJEB26058 | human |
| ERS2407391 | PRJEB26058 | human |
| ERS2407394 | PRJEB26058 | human |
| ERS2407392 | PRJEB26058 | human |
| ERS2407395 | PRJEB26058 | human |
| ERS2407396 | PRJEB26058 | human |
| ERS2407397 | PRJEB26058 | human |
| ERS2407398 | PRJEB26058 | human |
| ERS2407399 | PRJEB26058 | human |
| ERS2407400 | PRJEB26058 | human |
| ERS2407401 | PRJEB26058 | human |
| ERS2407402 | PRJEB26058 | human |
| ERS2407404 | PRJEB26058 | human |
| ERS2407403 | PRJEB26058 | human |
| ERS2407405 | PRJEB26058 | human |
| ERS2407406 | PRJEB26058 | human |
| ERS2407408 | PRJEB26058 | human |
| ERS2407407 | PRJEB26058 | human |
| ERS2407409 | PRJEB26058 | human |
| ERS2407410 | PRJEB26058 | human |
| ERS2407411 | PRJEB26058 | human |
| ERS2407412 | PRJEB26058 | human |
| ERS2407413 | PRJEB26058 | human |
| ERS2407415 | PRJEB26058 | human |
| ERS2407414 | PRJEB26058 | human |
| ERS2407416 | PRJEB26058 | human |
| ERS2407417 | PRJEB26058 | human |
| ERS2407419 | PRJEB26058 | human |
| ERS2407418 | PRJEB26058 | human |
| ERS2407420 | PRJEB26058 | human |
| ERS2407421 | PRJEB26058 | human |
| ERS2407422 | PRJEB26058 | human |
| ERS2407423 | PRJEB26058 | human |
| ERS2407424 | PRJEB26058 | human |
| ERS2407425 | PRJEB26058 | human |
| ERS2407427 | PRJEB26058 | human |
| ERS2407428 | PRJEB26058 | human |
| ERS2407426 | PRJEB26058 | human |
| ERS2407429 | PRJEB26058 | human |
| ERS2407432 | PRJEB26058 | human |
| ERS2407434 | PRJEB26058 | human |
| ERS2407433 | PRJEB26058 | human |
| ERS2407439 | PRJEB26058 | human |
| ERS2407435 | PRJEB26058 | human |
| ERS2407436 | PRJEB26058 | human |
| ERS2407430 | PRJEB26058 | human |
| ERS2407438 | PRJEB26058 | human |
| ERS2407443 | PRJEB26058 | human |
| ERS2407441 | PRJEB26058 | human |
| ERS2407442 | PRJEB26058 | human |
| ERS2407440 | PRJEB26058 | human |
| ERS2407444 | PRJEB26058 | human |
| ERS2407446 | PRJEB26058 | human |
| ERS2407431 | PRJEB26058 | human |
| ERS2407447 | PRJEB26058 | human |
| ERS2407448 | PRJEB26058 | human |
| ERS2407449 | PRJEB26058 | human |
| ERS2407450 | PRJEB26058 | human |
| ERS2407455 | PRJEB26058 | human |
| ERS2407453 | PRJEB26058 | human |
| ERS2407457 | PRJEB26058 | human |
| ERS2407456 | PRJEB26058 | human |
| ERS2407458 | PRJEB26058 | human |
| ERS2407459 | PRJEB26058 | human |
| ERS2407451 | PRJEB26058 | human |
| ERS2407445 | PRJEB26058 | human |
| ERS2407460 | PRJEB26058 | human |
| ERS2407461 | PRJEB26058 | human |
| ERS2407463 | PRJEB26058 | human |
| ERS2407462 | PRJEB26058 | human |
| ERS2407464 | PRJEB26058 | human |
| ERS2407465 | PRJEB26058 | human |
| ERS2407466 | PRJEB26058 | human |
| ERS2407468 | PRJEB26058 | human |
| ERS2407467 | PRJEB26058 | human |
| ERS2407454 | PRJEB26058 | human |
| ERS2407469 | PRJEB26058 | human |
| ERS2407475 | PRJEB26058 | human |
| ERS2407470 | PRJEB26058 | human |
| ERS2407471 | PRJEB26058 | human |
| ERS2407472 | PRJEB26058 | human |
| ERS2407473 | PRJEB26058 | human |
| ERS2407474 | PRJEB26058 | human |
| ERS2407476 | PRJEB26058 | human |
| ERS2407477 | PRJEB26058 | human |
| ERS2407478 | PRJEB26058 | human |
| ERS2407480 | PRJEB26058 | human |
| ERS2407479 | PRJEB26058 | human |
| ERS2407481 | PRJEB26058 | human |
| ERS2407482 | PRJEB26058 | human |
| ERS2407484 | PRJEB26058 | human |
| ERS2407483 | PRJEB26058 | human |
| ERS2407485 | PRJEB26058 | human |
| ERS2407486 | PRJEB26058 | human |
| ERS2407487 | PRJEB26058 | human |
| ERS2407488 | PRJEB26058 | human |
| ERS2407489 | PRJEB26058 | human |
| ERS2407490 | PRJEB26058 | human |
| ERS2407491 | PRJEB26058 | human |
| ERS2407493 | PRJEB26058 | human |
| ERS2407494 | PRJEB26058 | human |
| ERS2407492 | PRJEB26058 | human |
| ERS2407496 | PRJEB26058 | human |
| ERS2407495 | PRJEB26058 | human |
| ERS2407503 | PRJEB26058 | human |
| ERS2407500 | PRJEB26058 | human |
| ERS2407498 | PRJEB26058 | human |
| ERS2407497 | PRJEB26058 | human |
| ERS2407501 | PRJEB26058 | human |
| ERS2407499 | PRJEB26058 | human |
| ERS2407502 | PRJEB26058 | human |
| ERS2407505 | PRJEB26058 | human |
| ERS2407504 | PRJEB26058 | human |
| ERS2407506 | PRJEB26058 | human |
| ERS2407507 | PRJEB26058 | human |
| ERS2407508 | PRJEB26058 | human |
| ERS2407509 | PRJEB26058 | human |
| ERS2407511 | PRJEB26058 | human |
| ERS2407510 | PRJEB26058 | human |
| ERS2407512 | PRJEB26058 | human |
| ERS2407513 | PRJEB26058 | human |
| ERS2407516 | PRJEB26058 | human |
| ERS2407518 | PRJEB26058 | human |
| ERS2407517 | PRJEB26058 | human |
| ERS2407514 | PRJEB26058 | human |
| ERS2407515 | PRJEB26058 | human |
| ERS2407519 | PRJEB26058 | human |
| ERS2407520 | PRJEB26058 | human |
| ERS2407521 | PRJEB26058 | human |
| ERS2407522 | PRJEB26058 | human |
| ERS2407523 | PRJEB26058 | human |
| ERS2407527 | PRJEB26058 | human |
| ERS2407526 | PRJEB26058 | human |
| ERS2407524 | PRJEB26058 | human |
| ERS2407525 | PRJEB26058 | human |
| ERS2407528 | PRJEB26058 | human |
| ERS2407529 | PRJEB26058 | human |
| ERS2407531 | PRJEB26058 | human |
| ERS2407530 | PRJEB26058 | human |
| ERS2407532 | PRJEB26058 | human |
| ERS2407533 | PRJEB26058 | human |
| ERS2407534 | PRJEB26058 | human |
| ERS2407536 | PRJEB26058 | human |
| ERS2407535 | PRJEB26058 | human |
| ERS2407537 | PRJEB26058 | human |
| ERS2407538 | PRJEB26058 | human |
| ERS2407539 | PRJEB26058 | human |
| ERS2407542 | PRJEB26058 | human |
| ERS2407543 | PRJEB26058 | human |
| ERS2407544 | PRJEB26058 | human |
| ERS2407541 | PRJEB26058 | human |
| ERS2407540 | PRJEB26058 | human |
| ERS2407545 | PRJEB26058 | human |
| ERS2407546 | PRJEB26058 | human |
| ERS2407547 | PRJEB26058 | human |
| ERS2407548 | PRJEB26058 | human |
| ERS2407549 | PRJEB26058 | human |
| ERS2407552 | PRJEB26058 | human |
| ERS2407551 | PRJEB26058 | human |
| ERS2407550 | PRJEB26058 | human |
| ERS2407554 | PRJEB26058 | human |
| ERS2407553 | PRJEB26058 | human |
| ERS2407556 | PRJEB26058 | human |
| ERS2407555 | PRJEB26058 | human |
| ERS2407557 | PRJEB26058 | human |
| ERS2407558 | PRJEB26058 | human |
| ERS2407559 | PRJEB26058 | human |
| ERS2407560 | PRJEB26058 | human |
| ERS2407561 | PRJEB26058 | human |
| ERS2407562 | PRJEB26058 | human |
| ERS2407563 | PRJEB26058 | human |
| ERS2407565 | PRJEB26058 | human |
| ERS2407566 | PRJEB26058 | human |
| ERS2407564 | PRJEB26058 | human |
| ERS2407567 | PRJEB26058 | human |
| ERS2407568 | PRJEB26058 | human |
| ERS2407569 | PRJEB26058 | human |
| ERS2407570 | PRJEB26058 | human |
| ERS2407571 | PRJEB26058 | human |
| ERS2407572 | PRJEB26058 | human |
| ERS2407574 | PRJEB26058 | human |
| ERS2407576 | PRJEB26058 | human |
| ERS2407575 | PRJEB26058 | human |
| ERS2407577 | PRJEB26058 | human |
| ERS2407578 | PRJEB26058 | human |
| ERS2407579 | PRJEB26058 | human |
| ERS2407573 | PRJEB26058 | human |
| ERS2407580 | PRJEB26058 | human |
| ERS2407581 | PRJEB26058 | human |
| ERS2407584 | PRJEB26058 | human |
| ERS2407583 | PRJEB26058 | human |
| ERS2407582 | PRJEB26058 | human |
| ERS2407585 | PRJEB26058 | human |
| ERS2407587 | PRJEB26058 | human |
| ERS2407586 | PRJEB26058 | human |
| ERS2407588 | PRJEB26058 | human |
| ERS2407589 | PRJEB26058 | human |
| ERS2407590 | PRJEB26058 | human |
| ERS2407592 | PRJEB26058 | human |
| ERS2407591 | PRJEB26058 | human |
| ERS2407593 | PRJEB26058 | human |
| ERS2407595 | PRJEB26058 | human |
| ERS2407594 | PRJEB26058 | human |
| ERS2407596 | PRJEB26058 | human |
| ERS2407597 | PRJEB26058 | human |
| ERS2407598 | PRJEB26058 | human |
| ERS2407599 | PRJEB26058 | human |
| ERS2407600 | PRJEB26058 | human |
| ERS2407601 | PRJEB26058 | human |
| ERS2407604 | PRJEB26058 | human |
| ERS2407603 | PRJEB26058 | human |
| ERS2407602 | PRJEB26058 | human |
| ERS2407605 | PRJEB26058 | human |
| ERS2407609 | PRJEB26058 | human |
| ERS2407611 | PRJEB26058 | human |
| ERS2407612 | PRJEB26058 | human |
| ERS2407613 | PRJEB26058 | human |
| ERS2407614 | PRJEB26058 | human |
| ERS2407615 | PRJEB26058 | human |
| ERS2407616 | PRJEB26058 | human |
| ERS2407617 | PRJEB26058 | human |
| ERS2407606 | PRJEB26058 | human |
| ERS2407607 | PRJEB26058 | human |
| ERS2407608 | PRJEB26058 | human |
| ERS2407610 | PRJEB26058 | human |
| ERS2407618 | PRJEB26058 | human |
| ERS2407620 | PRJEB26058 | human |
| ERS2407619 | PRJEB26058 | human |
| ERS2407627 | PRJEB26058 | human |
| ERS2407621 | PRJEB26058 | human |
| ERS2407622 | PRJEB26058 | human |
| ERS2407623 | PRJEB26058 | human |
| ERS2407624 | PRJEB26058 | human |
| ERS2407628 | PRJEB26058 | human |
| ERS2407625 | PRJEB26058 | human |
| ERS2407626 | PRJEB26058 | human |
| ERS2407629 | PRJEB26058 | human |
| ERS2407630 | PRJEB26058 | human |
| ERS2407631 | PRJEB26058 | human |
| ERS2407632 | PRJEB26058 | human |
| ERS2407633 | PRJEB26058 | human |
| ERS2407634 | PRJEB26058 | human |
| ERS2407637 | PRJEB26058 | human |
| ERS2407636 | PRJEB26058 | human |
| ERS2407639 | PRJEB26058 | human |
| ERS2407638 | PRJEB26058 | human |
| ERS2407635 | PRJEB26058 | human |
| ERS2407641 | PRJEB26058 | human |
| ERS2407640 | PRJEB26058 | human |
| ERS2407642 | PRJEB26058 | human |
| ERS2407643 | PRJEB26058 | human |
| ERS2407644 | PRJEB26058 | human |
| ERS2407646 | PRJEB26058 | human |
| ERS2407647 | PRJEB26058 | human |
| ERS2407649 | PRJEB26058 | human |
| ERS2407648 | PRJEB26058 | human |
| ERS2407650 | PRJEB26058 | human |
| ERS2407651 | PRJEB26058 | human |
| ERS2407652 | PRJEB26058 | human |
| ERS2407658 | PRJEB26058 | human |
| ERS2407653 | PRJEB26058 | human |
| ERS2407654 | PRJEB26058 | human |
| ERS2407655 | PRJEB26058 | human |
| ERS2407656 | PRJEB26058 | human |
| ERS2407657 | PRJEB26058 | human |
| ERS2407659 | PRJEB26058 | human |
| ERS2407662 | PRJEB26058 | human |
| ERS2407660 | PRJEB26058 | human |
| ERS2407661 | PRJEB26058 | human |
| ERS2407663 | PRJEB26058 | human |
| ERS2407665 | PRJEB26058 | human |
| ERS2407664 | PRJEB26058 | human |
| ERS2407666 | PRJEB26058 | human |
| ERS2407667 | PRJEB26058 | human |
| ERS2407668 | PRJEB26058 | human |
| ERS2407669 | PRJEB26058 | human |
| ERS2407670 | PRJEB26058 | human |
| ERS2407671 | PRJEB26058 | human |
| ERS2407672 | PRJEB26058 | human |
| ERS2407673 | PRJEB26058 | human |
| ERS2407674 | PRJEB26058 | human |
| ERS2407675 | PRJEB26058 | human |
| ERS2407676 | PRJEB26058 | human |
| ERS2407677 | PRJEB26058 | human |
| ERS2407678 | PRJEB26058 | human |
| ERS2407679 | PRJEB26058 | human |
| ERS2407680 | PRJEB26058 | human |
| ERS2407681 | PRJEB26058 | human |
| ERS2407682 | PRJEB26058 | human |
| ERS2407683 | PRJEB26058 | human |
| ERS2407684 | PRJEB26058 | human |
| ERS2407685 | PRJEB26058 | human |
| ERS2407686 | PRJEB26058 | human |
| ERS2407687 | PRJEB26058 | human |
| ERS2407688 | PRJEB26058 | human |
| ERS2407689 | PRJEB26058 | human |
| ERS2407690 | PRJEB26058 | human |
| ERS2407691 | PRJEB26058 | human |
| ERS2407692 | PRJEB26058 | human |
| ERS2407693 | PRJEB26058 | human |
| ERS2407694 | PRJEB26058 | human |
| ERS2407695 | PRJEB26058 | human |
| ERS2407696 | PRJEB26058 | human |
| ERS2407697 | PRJEB26058 | human |
| ERS2407698 | PRJEB26058 | human |
| ERS2407699 | PRJEB26058 | human |
| ERS2407700 | PRJEB26058 | human |
| ERS2407702 | PRJEB26058 | human |
| ERS2407703 | PRJEB26058 | human |
| ERS2407704 | PRJEB26058 | human |
| ERS2407705 | PRJEB26058 | human |
| ERS2407706 | PRJEB26058 | human |
| ERS2407707 | PRJEB26058 | human |
| ERS2407708 | PRJEB26058 | human |
| ERS2407709 | PRJEB26058 | human |
| ERS2407710 | PRJEB26058 | human |
| ERS2407711 | PRJEB26058 | human |
| ERS2407712 | PRJEB26058 | human |
| ERS14411926 | PRJEB58647 | human |
| ERS14411927 | PRJEB58647 | human |
| ERS14411928 | PRJEB58647 | human |
| ERS14411929 | PRJEB58647 | human |
| ERS14411930 | PRJEB58647 | human |
| ERS14411931 | PRJEB58647 | human |
| ERS14411932 | PRJEB58647 | human |
| ERS14411933 | PRJEB58647 | human |
| ERS14411934 | PRJEB58647 | human |
| ERS14411935 | PRJEB58647 | human |
| ERS14411936 | PRJEB58647 | human |
| ERS14411937 | PRJEB58647 | human |
| ERS14411938 | PRJEB58647 | human |
| ERS14411939 | PRJEB58647 | human |
| ERS14411940 | PRJEB58647 | human |
| ERS14411941 | PRJEB58647 | human |
| ERS14411942 | PRJEB58647 | human |
| ERS14411943 | PRJEB58647 | human |
| ERS14411944 | PRJEB58647 | human |
| ERS14411945 | PRJEB58647 | human |
| ERS14411946 | PRJEB58647 | human |
| ERS14411947 | PRJEB58647 | human |
| ERS14411948 | PRJEB58647 | human |
| ERS14411949 | PRJEB58647 | human |
| ERS14411950 | PRJEB58647 | human |
| ERS14411951 | PRJEB58647 | human |
| ERS14411952 | PRJEB58647 | human |
| ERS14411953 | PRJEB58647 | human |
| ERS14411954 | PRJEB58647 | human |
| ERS14411955 | PRJEB58647 | human |
| ERS14411956 | PRJEB58647 | human |
| ERS14411957 | PRJEB58647 | human |
| ERS14411958 | PRJEB58647 | human |
| ERS14411959 | PRJEB58647 | human |
| ERS14411960 | PRJEB58647 | human |
| ERS14411961 | PRJEB58647 | human |
| ERS14411962 | PRJEB58647 | human |
| ERS14411963 | PRJEB58647 | human |
| ERS14411964 | PRJEB58647 | human |
| ERS14411965 | PRJEB58647 | human |
| ERS14411966 | PRJEB58647 | human |
| ERS14411967 | PRJEB58647 | human |
| ERS14411968 | PRJEB58647 | human |
| ERS14411969 | PRJEB58647 | human |
| ERS14411970 | PRJEB58647 | human |
| ERS14411971 | PRJEB58647 | human |
| ERS14411972 | PRJEB58647 | human |
| ERS14411973 | PRJEB58647 | human |
| ERS14411974 | PRJEB58647 | human |
| ERS14411975 | PRJEB58647 | human |
| ERS14411976 | PRJEB58647 | human |
| ERS14411977 | PRJEB58647 | human |
| ERS14411978 | PRJEB58647 | human |
| ERS14411979 | PRJEB58647 | human |
| ERS14411980 | PRJEB58647 | human |
| ERS14411981 | PRJEB58647 | human |
| ERS14411982 | PRJEB58647 | human |
| ERS14411983 | PRJEB58647 | human |
| ERS14411984 | PRJEB58647 | human |
| ERS14411985 | PRJEB58647 | human |
| ERS14411986 | PRJEB58647 | human |
| ERS14411987 | PRJEB58647 | human |
| ERS14411988 | PRJEB58647 | human |
| ERS14411989 | PRJEB58647 | human |
| ERS14411990 | PRJEB58647 | human |
| ERS14411991 | PRJEB58647 | human |
| ERS14411992 | PRJEB58647 | human |
| ERS14411993 | PRJEB58647 | human |
| ERS14411994 | PRJEB58647 | human |
| ERS14411995 | PRJEB58647 | human |
| ERS14411996 | PRJEB58647 | human |
| ERS14411997 | PRJEB58647 | human |
| ERS14411998 | PRJEB58647 | human |
| ERS14411999 | PRJEB58647 | human |
| ERS14412000 | PRJEB58647 | human |
| ERS14412001 | PRJEB58647 | human |
| ERS14412002 | PRJEB58647 | human |
| ERS14412003 | PRJEB58647 | human |
| ERS14412004 | PRJEB58647 | human |
| ERS14412005 | PRJEB58647 | human |
| ERS14412006 | PRJEB58647 | human |
| ERS14412007 | PRJEB58647 | human |
| ERS14412008 | PRJEB58647 | human |
| ERS14412009 | PRJEB58647 | human |
| ERS14412010 | PRJEB58647 | human |
| ERS14412011 | PRJEB58647 | human |
| ERS14412012 | PRJEB58647 | human |
| ERS14412013 | PRJEB58647 | human |
| ERS14412014 | PRJEB58647 | human |
| ERS14412015 | PRJEB58647 | human |
| ERS14412016 | PRJEB58647 | human |
| ERS14412017 | PRJEB58647 | human |
| ERS14412018 | PRJEB58647 | human |
| ERS14412019 | PRJEB58647 | human |
| ERS14412020 | PRJEB58647 | human |
| ERS14412021 | PRJEB58647 | human |
| ERS14412022 | PRJEB58647 | human |
| ERS14412023 | PRJEB58647 | human |
| ERS14412024 | PRJEB58647 | human |
| ERS14412025 | PRJEB58647 | human |
| ERS14412026 | PRJEB58647 | human |
| ERS14412027 | PRJEB58647 | human |
| ERS14412028 | PRJEB58647 | human |
| ERS14412029 | PRJEB58647 | human |
| ERS14412030 | PRJEB58647 | human |
| ERS14412031 | PRJEB58647 | human |
| ERS14412032 | PRJEB58647 | human |
| ERS14412033 | PRJEB58647 | human |
| ERS14412034 | PRJEB58647 | human |
| ERS14412035 | PRJEB58647 | human |
| ERS14412036 | PRJEB58647 | human |
| ERS14412037 | PRJEB58647 | human |
| ERS14412038 | PRJEB58647 | human |
| ERS14412039 | PRJEB58647 | human |
| ERS14412040 | PRJEB58647 | human |
| ERS14412041 | PRJEB58647 | human |
| ERS14412042 | PRJEB58647 | human |
| ERS14412043 | PRJEB58647 | human |
| ERS14412044 | PRJEB58647 | human |
| ERS14412045 | PRJEB58647 | human |
| ERS14412046 | PRJEB58647 | human |
| ERS14412047 | PRJEB58647 | human |
| ERS14412048 | PRJEB58647 | human |
| ERS14412049 | PRJEB58647 | human |
| ERS14412050 | PRJEB58647 | human |
| ERS14412051 | PRJEB58647 | human |
| ERS14412052 | PRJEB58647 | human |
| ERS14412053 | PRJEB58647 | human |
| ERS14412054 | PRJEB58647 | human |
| ERS14412055 | PRJEB58647 | human |
| ERS14412056 | PRJEB58647 | human |
| ERS14412057 | PRJEB58647 | human |
| ERS14412058 | PRJEB58647 | human |
| ERS14412059 | PRJEB58647 | human |
| ERS14412060 | PRJEB58647 | human |
| ERS14412061 | PRJEB58647 | human |
| ERS14412062 | PRJEB58647 | human |
| ERS14412063 | PRJEB58647 | human |
| ERS14412064 | PRJEB58647 | human |
| ERS14412065 | PRJEB58647 | human |
| ERS14412066 | PRJEB58647 | human |
| ERS14412067 | PRJEB58647 | human |
| ERS14412068 | PRJEB58647 | human |
| ERS14412069 | PRJEB58647 | human |
| ERS14412070 | PRJEB58647 | human |
| ERS14412071 | PRJEB58647 | human |
| ERS14412072 | PRJEB58647 | human |
| ERS14412073 | PRJEB58647 | human |
| ERS14412074 | PRJEB58647 | human |
| ERS14412075 | PRJEB58647 | human |
| ERS14412076 | PRJEB58647 | human |
| ERS14412077 | PRJEB58647 | human |
| ERS14412078 | PRJEB58647 | human |
| ERS14412079 | PRJEB58647 | human |
| ERS14412080 | PRJEB58647 | human |
| ERS14412081 | PRJEB58647 | human |
| ERS14412082 | PRJEB58647 | human |
| ERS14412083 | PRJEB58647 | human |
| ERS14412084 | PRJEB58647 | human |
| ERS14412085 | PRJEB58647 | human |
| ERS14412086 | PRJEB58647 | human |
| ERS14412087 | PRJEB58647 | human |
| ERS14412088 | PRJEB58647 | human |
| ERS14412089 | PRJEB58647 | human |
| ERS14412090 | PRJEB58647 | human |
| ERS14412091 | PRJEB58647 | human |
| ERS14412092 | PRJEB58647 | human |
| ERS14412093 | PRJEB58647 | human |
| ERS14412094 | PRJEB58647 | human |
| ERS14412095 | PRJEB58647 | human |
| ERS14412096 | PRJEB58647 | human |
| ERS14412097 | PRJEB58647 | human |
| ERS14412098 | PRJEB58647 | human |
| ERS14412099 | PRJEB58647 | human |
| ERS14412100 | PRJEB58647 | human |
| ERS14412101 | PRJEB58647 | human |
| ERS14412102 | PRJEB58647 | human |
| ERS14412103 | PRJEB58647 | human |
| ERS14412104 | PRJEB58647 | human |
| ERS14412105 | PRJEB58647 | human |
| ERS14412106 | PRJEB58647 | human |
| ERS14412107 | PRJEB58647 | human |
| ERS14412108 | PRJEB58647 | human |
| ERS14412109 | PRJEB58647 | human |
| ERS14412110 | PRJEB58647 | human |
| ERS14412111 | PRJEB58647 | human |
| ERS14412112 | PRJEB58647 | human |
| ERS14412113 | PRJEB58647 | human |
| ERS14412114 | PRJEB58647 | human |
| ERS14412115 | PRJEB58647 | human |
| ERS14412116 | PRJEB58647 | human |
| ERS14412117 | PRJEB58647 | human |
| ERS14412118 | PRJEB58647 | human |
| ERS14412119 | PRJEB58647 | human |
| ERS14412120 | PRJEB58647 | human |
| ERS14412121 | PRJEB58647 | human |
| ERS14412122 | PRJEB58647 | human |
| ERS14412123 | PRJEB58647 | human |
| ERS14412124 | PRJEB58647 | human |
| ERS14412125 | PRJEB58647 | human |
| ERS14412126 | PRJEB58647 | human |
| ERS14412127 | PRJEB58647 | human |
| ERS14412128 | PRJEB58647 | human |
| ERS14412129 | PRJEB58647 | human |
| ERS14412130 | PRJEB58647 | human |
| ERS14412131 | PRJEB58647 | human |
| ERS14412132 | PRJEB58647 | human |
| ERS14412133 | PRJEB58647 | human |
| ERS14412134 | PRJEB58647 | human |
| ERS14412135 | PRJEB58647 | human |
| ERS14412136 | PRJEB58647 | human |
| ERS14412137 | PRJEB58647 | human |
| ERS14412138 | PRJEB58647 | human |
| ERS14412139 | PRJEB58647 | human |
| ERS14412140 | PRJEB58647 | human |
| ERS14412141 | PRJEB58647 | human |
| ERS14412142 | PRJEB58647 | human |
| ERS14412143 | PRJEB58647 | human |
| ERS14412144 | PRJEB58647 | human |
| ERS14412145 | PRJEB58647 | human |
| ERS14412146 | PRJEB58647 | human |
| ERS14412147 | PRJEB58647 | human |
| ERS14412148 | PRJEB58647 | human |
| ERS14412149 | PRJEB58647 | human |
| ERS14412150 | PRJEB58647 | human |
| ERS14412151 | PRJEB58647 | human |
| ERS14412152 | PRJEB58647 | human |
| ERS14412153 | PRJEB58647 | human |
| ERS14412154 | PRJEB58647 | human |
| ERS14412155 | PRJEB58647 | human |
| ERS14412156 | PRJEB58647 | human |
| ERS14412157 | PRJEB58647 | human |
| ERS14412158 | PRJEB58647 | human |
| ERS14412159 | PRJEB58647 | human |
| ERS14412160 | PRJEB58647 | human |
| ERS14412161 | PRJEB58647 | human |
| ERS14412162 | PRJEB58647 | human |
| ERS14412163 | PRJEB58647 | human |
| ERS14412164 | PRJEB58647 | human |
| ERS14412165 | PRJEB58647 | human |
| ERS14412166 | PRJEB58647 | human |
| ERS14412167 | PRJEB58647 | human |
| ERS14412168 | PRJEB58647 | human |
| ERS14412169 | PRJEB58647 | human |
| ERS14412170 | PRJEB58647 | human |
| ERS14412171 | PRJEB58647 | human |
| ERS14412172 | PRJEB58647 | human |
| ERS14412173 | PRJEB58647 | human |
| ERS14412174 | PRJEB58647 | human |
| ERS14412175 | PRJEB58647 | human |
| ERS14412176 | PRJEB58647 | human |
| ERS14412177 | PRJEB58647 | human |
| ERS14412178 | PRJEB58647 | human |
| ERS14412179 | PRJEB58647 | human |
| ERS14412180 | PRJEB58647 | human |
| ERS14412181 | PRJEB58647 | human |
| ERS14412182 | PRJEB58647 | human |
| ERS14412183 | PRJEB58647 | human |
| ERS14412184 | PRJEB58647 | human |
| ERS14412185 | PRJEB58647 | human |
| ERS14412186 | PRJEB58647 | human |
| ERS14412187 | PRJEB58647 | human |
| ERS14412188 | PRJEB58647 | human |
| ERS14412189 | PRJEB58647 | human |
| ERS14412190 | PRJEB58647 | human |
| ERS14412191 | PRJEB58647 | human |
| ERS14412192 | PRJEB58647 | human |
| ERS14412193 | PRJEB58647 | human |
| ERS14412194 | PRJEB58647 | human |
| ERS14412195 | PRJEB58647 | human |
| ERS14412196 | PRJEB58647 | human |
| ERS14412197 | PRJEB58647 | human |
| ERS14412198 | PRJEB58647 | human |
| ERS14412199 | PRJEB58647 | human |
| ERS14412200 | PRJEB58647 | human |
| ERS14412201 | PRJEB58647 | human |
| ERS14412202 | PRJEB58647 | human |
| ERS14412203 | PRJEB58647 | human |
| ERS14412204 | PRJEB58647 | human |
| ERS14412205 | PRJEB58647 | human |
| ERS14412206 | PRJEB58647 | human |
| ERS14412207 | PRJEB58647 | human |
| ERS14412208 | PRJEB58647 | human |
| ERS14412209 | PRJEB58647 | human |
| ERS14412210 | PRJEB58647 | human |
| ERS14412211 | PRJEB58647 | human |
| ERS14412212 | PRJEB58647 | human |
| ERS14412213 | PRJEB58647 | human |
| ERS14412214 | PRJEB58647 | human |
| ERS14412215 | PRJEB58647 | human |
| ERS14412216 | PRJEB58647 | human |
| ERS14412217 | PRJEB58647 | human |
| ERS14412218 | PRJEB58647 | human |
| ERS14412219 | PRJEB58647 | human |
| ERS14412220 | PRJEB58647 | human |
| ERS14412221 | PRJEB58647 | human |
| ERS14412222 | PRJEB58647 | human |
| ERS14412223 | PRJEB58647 | human |
| ERS14412224 | PRJEB58647 | human |
| ERS14412225 | PRJEB58647 | human |
| ERS14412226 | PRJEB58647 | human |
| ERS14412227 | PRJEB58647 | human |
| ERS14412228 | PRJEB58647 | human |
| ERS14412229 | PRJEB58647 | human |
| ERS14412230 | PRJEB58647 | human |
| ERS14412231 | PRJEB58647 | human |
| ERS14412232 | PRJEB58647 | human |
| ERS14412233 | PRJEB58647 | human |
| ERS14412234 | PRJEB58647 | human |
| ERS14412235 | PRJEB58647 | human |
| ERS14412236 | PRJEB58647 | human |
| ERS14412237 | PRJEB58647 | human |
| ERS14412238 | PRJEB58647 | human |
| ERS14412239 | PRJEB58647 | human |
| ERS14412240 | PRJEB58647 | human |
| ERS14412241 | PRJEB58647 | human |
| ERS14412242 | PRJEB58647 | human |
| ERS14412243 | PRJEB58647 | human |
| ERS14412244 | PRJEB58647 | human |
| ERS14412245 | PRJEB58647 | human |
| ERS14412246 | PRJEB58647 | human |
| ERS14412247 | PRJEB58647 | human |
| ERS14412248 | PRJEB58647 | human |
| ERS14412249 | PRJEB58647 | human |
| ERS14412250 | PRJEB58647 | human |
| ERS14412251 | PRJEB58647 | human |
| ERS14412252 | PRJEB58647 | human |
| ERS14412253 | PRJEB58647 | human |
| ERS14412254 | PRJEB58647 | human |
| ERS14412255 | PRJEB58647 | human |
| ERS14412256 | PRJEB58647 | human |
| ERS14412257 | PRJEB58647 | human |
| ERS14412258 | PRJEB58647 | human |
| ERS14412259 | PRJEB58647 | human |
| ERS14412260 | PRJEB58647 | human |
| ERS14412261 | PRJEB58647 | human |
| ERS14412262 | PRJEB58647 | human |
| ERS14412263 | PRJEB58647 | human |
| ERS14412264 | PRJEB58647 | human |
| ERS14412265 | PRJEB58647 | human |
| ERS14412266 | PRJEB58647 | human |
| ERS14412267 | PRJEB58647 | human |
| ERS14412268 | PRJEB58647 | human |
| ERS14412269 | PRJEB58647 | human |
| ERS14412270 | PRJEB58647 | human |
| ERS14412271 | PRJEB58647 | human |
| ERS14412272 | PRJEB58647 | human |
| ERS14412273 | PRJEB58647 | human |
| ERS14412274 | PRJEB58647 | human |
| ERS14412275 | PRJEB58647 | human |
| ERS14412276 | PRJEB58647 | human |
| ERS14412277 | PRJEB58647 | human |
| ERS14412278 | PRJEB58647 | human |
| ERS14412279 | PRJEB58647 | human |
| ERS14412280 | PRJEB58647 | human |
| ERS14412281 | PRJEB58647 | human |
| ERS14412282 | PRJEB58647 | human |
| ERS14412283 | PRJEB58647 | human |
| ERS14412284 | PRJEB58647 | human |
| ERS14412285 | PRJEB58647 | human |
| ERS14412286 | PRJEB58647 | human |
| ERS14412287 | PRJEB58647 | human |
| ERS14412288 | PRJEB58647 | human |
| ERS14412289 | PRJEB58647 | human |
| ERS14412290 | PRJEB58647 | human |
| ERS14412291 | PRJEB58647 | human |
| ERS14412292 | PRJEB58647 | human |
| ERS14412293 | PRJEB58647 | human |
| ERS14412294 | PRJEB58647 | human |
| ERS14412295 | PRJEB58647 | human |
| ERS14412296 | PRJEB58647 | human |
| ERS14412297 | PRJEB58647 | human |
| ERS14412298 | PRJEB58647 | human |
| ERS14412299 | PRJEB58647 | human |
| ERS14412300 | PRJEB58647 | human |
| ERS14412301 | PRJEB58647 | human |
| ERS14412302 | PRJEB58647 | human |
| ERS14412303 | PRJEB58647 | human |
| ERS14412304 | PRJEB58647 | human |
| ERS14412305 | PRJEB58647 | human |
| ERS14412306 | PRJEB58647 | human |
| ERS14412307 | PRJEB58647 | human |
| ERS14412308 | PRJEB58647 | human |
| ERS14412309 | PRJEB58647 | human |
| ERS14412310 | PRJEB58647 | human |
| ERS14412311 | PRJEB58647 | human |
| ERS14412312 | PRJEB58647 | human |
| ERS14412313 | PRJEB58647 | human |
| ERS14412314 | PRJEB58647 | human |
| ERS14412315 | PRJEB58647 | human |
| ERS14412316 | PRJEB58647 | human |
| ERS14412317 | PRJEB58647 | human |
| ERS14412318 | PRJEB58647 | human |
| ERS14412319 | PRJEB58647 | human |
| ERS14412320 | PRJEB58647 | human |
| ERS14412321 | PRJEB58647 | human |
| ERS14412322 | PRJEB58647 | human |
| ERS14412323 | PRJEB58647 | human |
| ERS14412324 | PRJEB58647 | human |
| ERS14412325 | PRJEB58647 | human |
| ERS14412326 | PRJEB58647 | human |
| ERS14412327 | PRJEB58647 | human |
| ERS12476411 | PRJEB54813 | food |
| ERS12476398 | PRJEB54813 | food |
| ERS12476392 | PRJEB54813 | food |
| ERS12476381 | PRJEB54813 | food |
| ERS12476393 | PRJEB54813 | food |
| ERS12476395 | PRJEB54813 | food |
| ERS12476403 | PRJEB54813 | food |
| ERS12476413 | PRJEB54813 | food |
| ERS12476396 | PRJEB54813 | food |
| ERS12476397 | PRJEB54813 | food |
| ERS12476382 | PRJEB54813 | food |
| ERS12476410 | PRJEB54813 | food |
| ERS12476394 | PRJEB54813 | food |
| ERS12476380 | PRJEB54813 | food |
| ERS12476400 | PRJEB54813 | food |
| ERS12476401 | PRJEB54813 | food |
| ERS12476407 | PRJEB54813 | food |
| ERS12476391 | PRJEB54813 | food |
| ERS12476399 | PRJEB54813 | food |
| ERS12476383 | PRJEB54813 | food |
| ERS12476406 | PRJEB54813 | food |
| ERS12476386 | PRJEB54813 | food |
| ERS12476412 | PRJEB54813 | food |
| ERS12476404 | PRJEB54813 | food |
| ERS12476405 | PRJEB54813 | food |
| ERS12476402 | PRJEB54813 | food |
| ERS12476408 | PRJEB54813 | food |
| ERS12476387 | PRJEB54813 | food |
| ERS12476384 | PRJEB54813 | food |
| ERS12476385 | PRJEB54813 | food |
| ERS12476389 | PRJEB54813 | food |
| ERS12476409 | PRJEB54813 | food |
| ERS12476390 | PRJEB54813 | food |
| ERS12476388 | PRJEB54813 | food |
| ERS12476379 | PRJEB54813 | food |
| ERS12476456 | PRJEB54813 | food |
| ERS12476423 | PRJEB54813 | food |
| ERS12476516 | PRJEB54813 | food |
| ERS12476417 | PRJEB54813 | food |
| ERS12476520 | PRJEB54813 | food |
| ERS12476462 | PRJEB54813 | food |
| ERS12476519 | PRJEB54813 | food |
| ERS12476518 | PRJEB54813 | food |
| ERS12476507 | PRJEB54813 | food |
| ERS12476469 | PRJEB54813 | food |
| ERS12476464 | PRJEB54813 | food |
| ERS12476421 | PRJEB54813 | food |
| ERS12476509 | PRJEB54813 | food |
| ERS12476463 | PRJEB54813 | food |
| ERS12476414 | PRJEB54813 | food |
| ERS12476465 | PRJEB54813 | food |
| ERS12476457 | PRJEB54813 | food |
| ERS12476466 | PRJEB54813 | food |
| ERS12476424 | PRJEB54813 | food |
| ERS12476515 | PRJEB54813 | food |
| ERS12476490 | PRJEB54813 | food |
| ERS12476429 | PRJEB54813 | food |
| ERS12476472 | PRJEB54813 | food |
| ERS12476491 | PRJEB54813 | food |
| ERS12476470 | PRJEB54813 | food |
| ERS12476439 | PRJEB54813 | food |
| ERS12476418 | PRJEB54813 | food |
| ERS12476458 | PRJEB54813 | food |
| ERS12476431 | PRJEB54813 | food |
| ERS12476524 | PRJEB54813 | food |
| ERS12476492 | PRJEB54813 | food |
| ERS12476513 | PRJEB54813 | food |
| ERS12476525 | PRJEB54813 | food |
| ERS12476425 | PRJEB54813 | food |
| ERS12476494 | PRJEB54813 | food |
| ERS12476497 | PRJEB54813 | food |
| ERS12476521 | PRJEB54813 | food |
| ERS12476436 | PRJEB54813 | food |
| ERS12476517 | PRJEB54813 | food |
| ERS12476426 | PRJEB54813 | food |
| ERS12476514 | PRJEB54813 | food |
| ERS12476523 | PRJEB54813 | food |
| ERS12476522 | PRJEB54813 | food |
| ERS12476435 | PRJEB54813 | food |
| ERS12476434 | PRJEB54813 | food |
| ERS12476471 | PRJEB54813 | food |
| ERS12476473 | PRJEB54813 | food |
| ERS12476455 | PRJEB54813 | food |
| ERS12476475 | PRJEB54813 | food |
| ERS12476510 | PRJEB54813 | food |
| ERS12476415 | PRJEB54813 | food |
| ERS12476476 | PRJEB54813 | food |
| ERS12476502 | PRJEB54813 | food |
| ERS12476503 | PRJEB54813 | food |
| ERS12476422 | PRJEB54813 | food |
| ERS12476474 | PRJEB54813 | food |
| ERS12476430 | PRJEB54813 | food |
| ERS12476427 | PRJEB54813 | food |
| ERS12476477 | PRJEB54813 | food |
| ERS12476461 | PRJEB54813 | food |
| ERS12476454 | PRJEB54813 | food |
| ERS12476419 | PRJEB54813 | food |
| ERS12476459 | PRJEB54813 | food |
| ERS12476467 | PRJEB54813 | food |
| ERS12476468 | PRJEB54813 | food |
| ERS12476433 | PRJEB54813 | food |
| ERS12476432 | PRJEB54813 | food |
| ERS12476511 | PRJEB54813 | food |
| ERS12476420 | PRJEB54813 | food |
| ERS12476526 | PRJEB54813 | food |
| ERS12476416 | PRJEB54813 | food |
| ERS12476478 | PRJEB54813 | food |
| ERS12476428 | PRJEB54813 | food |
| ERS12476460 | PRJEB54813 | food |
| ERS12476479 | PRJEB54813 | food |
| ERS12476500 | PRJEB54813 | food |
| ERS12476437 | PRJEB54813 | food |
| ERS12476512 | PRJEB54813 | food |
| ERS12476489 | PRJEB54813 | food |
| ERS12476488 | PRJEB54813 | food |
| ERS12476443 | PRJEB54813 | food |
| ERS12476528 | PRJEB54813 | food |
| ERS12476529 | PRJEB54813 | food |
| ERS12476484 | PRJEB54813 | food |
| ERS12476501 | PRJEB54813 | food |
| ERS12476444 | PRJEB54813 | food |
| ERS12476440 | PRJEB54813 | food |
| ERS12476527 | PRJEB54813 | food |
| ERS12476446 | PRJEB54813 | food |
| ERS12476481 | PRJEB54813 | food |
| ERS12476530 | PRJEB54813 | food |
| ERS12476485 | PRJEB54813 | food |
| ERS12476449 | PRJEB54813 | food |
| ERS12476498 | PRJEB54813 | food |
| ERS12476506 | PRJEB54813 | food |
| ERS12476438 | PRJEB54813 | food |
| ERS12476445 | PRJEB54813 | food |
| ERS12476450 | PRJEB54813 | food |
| ERS12476486 | PRJEB54813 | food |
| ERS12476495 | PRJEB54813 | food |
| ERS12476482 | PRJEB54813 | food |
| ERS12476483 | PRJEB54813 | food |
| ERS12476447 | PRJEB54813 | food |
| ERS12476504 | PRJEB54813 | food |
| ERS12476448 | PRJEB54813 | food |
| ERS12476487 | PRJEB54813 | food |
| ERS12476499 | PRJEB54813 | food |
| ERS12476480 | PRJEB54813 | food |
| ERS12476505 | PRJEB54813 | food |
| ERS12476508 | PRJEB54813 | food |
| ERS12476531 | PRJEB54813 | food |
| ERS12476441 | PRJEB54813 | food |
| ERS12476451 | PRJEB54813 | food |
| ERS12476453 | PRJEB54813 | food |
| ERS12476493 | PRJEB54813 | food |
| ERS12476452 | PRJEB54813 | food |
| ERS12476442 | PRJEB54813 | food |
| ERS12476496 | PRJEB54813 | food |
| ERS12476709 | PRJEB54813 | food |
| ERS12476532 | PRJEB54813 | food |
| ERS12476690 | PRJEB54813 | food |
| ERS12476533 | PRJEB54813 | food |
| ERS12476710 | PRJEB54813 | food |
| ERS12476564 | PRJEB54813 | food |
| ERS12476566 | PRJEB54813 | food |
| ERS12476565 | PRJEB54813 | food |
| ERS12476534 | PRJEB54813 | food |
| ERS12476563 | PRJEB54813 | food |
| ERS12476711 | PRJEB54813 | food |
| ERS12476567 | PRJEB54813 | food |
| ERS12476693 | PRJEB54813 | food |
| ERS12476568 | PRJEB54813 | food |
| ERS12476569 | PRJEB54813 | food |
| ERS12476570 | PRJEB54813 | food |
| ERS12476535 | PRJEB54813 | food |
| ERS12476712 | PRJEB54813 | food |
| ERS12476713 | PRJEB54813 | food |
| ERS12476572 | PRJEB54813 | food |
| ERS12476571 | PRJEB54813 | food |
| ERS12476714 | PRJEB54813 | food |
| ERS12476574 | PRJEB54813 | food |
| ERS12476573 | PRJEB54813 | food |
| ERS12476536 | PRJEB54813 | food |
| ERS12476675 | PRJEB54813 | food |
| ERS12476705 | PRJEB54813 | food |
| ERS12476576 | PRJEB54813 | food |
| ERS12476575 | PRJEB54813 | food |
| ERS12476682 | PRJEB54813 | food |
| ERS12476676 | PRJEB54813 | food |
| ERS12476577 | PRJEB54813 | food |
| ERS12476691 | PRJEB54813 | food |
| ERS12476578 | PRJEB54813 | food |
| ERS12476683 | PRJEB54813 | food |
| ERS12476579 | PRJEB54813 | food |
| ERS12476685 | PRJEB54813 | food |
| ERS12476674 | PRJEB54813 | food |
| ERS12476686 | PRJEB54813 | food |
| ERS12476580 | PRJEB54813 | food |
| ERS12476684 | PRJEB54813 | food |
| ERS12476537 | PRJEB54813 | food |
| ERS12476581 | PRJEB54813 | food |
| ERS12476538 | PRJEB54813 | food |
| ERS12476582 | PRJEB54813 | food |
| ERS12476583 | PRJEB54813 | food |
| ERS12476715 | PRJEB54813 | food |
| ERS12476584 | PRJEB54813 | food |
| ERS12476585 | PRJEB54813 | food |
| ERS12476586 | PRJEB54813 | food |
| ERS12476716 | PRJEB54813 | food |
| ERS12476587 | PRJEB54813 | food |
| ERS12476588 | PRJEB54813 | food |
| ERS12476539 | PRJEB54813 | food |
| ERS12476720 | PRJEB54813 | food |
| ERS12476589 | PRJEB54813 | food |
| ERS12476694 | PRJEB54813 | food |
| ERS12476706 | PRJEB54813 | food |
| ERS12476591 | PRJEB54813 | food |
| ERS12476590 | PRJEB54813 | food |
| ERS12476540 | PRJEB54813 | food |
| ERS12476695 | PRJEB54813 | food |
| ERS12476707 | PRJEB54813 | food |
| ERS12476593 | PRJEB54813 | food |
| ERS12476541 | PRJEB54813 | food |
| ERS12476592 | PRJEB54813 | food |
| ERS12476594 | PRJEB54813 | food |
| ERS12476595 | PRJEB54813 | food |
| ERS12476596 | PRJEB54813 | food |
| ERS12476597 | PRJEB54813 | food |
| ERS12476562 | PRJEB54813 | food |
| ERS12476672 | PRJEB54813 | food |
| ERS12476598 | PRJEB54813 | food |
| ERS12476599 | PRJEB54813 | food |
| ERS12476692 | PRJEB54813 | food |
| ERS12476542 | PRJEB54813 | food |
| ERS12476600 | PRJEB54813 | food |
| ERS12476543 | PRJEB54813 | food |
| ERS12476717 | PRJEB54813 | food |
| ERS12476696 | PRJEB54813 | food |
| ERS12476601 | PRJEB54813 | food |
| ERS12476602 | PRJEB54813 | food |
| ERS12476604 | PRJEB54813 | food |
| ERS12476544 | PRJEB54813 | food |
| ERS12476607 | PRJEB54813 | food |
| ERS12476606 | PRJEB54813 | food |
| ERS12476605 | PRJEB54813 | food |
| ERS12476718 | PRJEB54813 | food |
| ERS12476603 | PRJEB54813 | food |
| ERS12476545 | PRJEB54813 | food |
| ERS12476608 | PRJEB54813 | food |
| ERS12476609 | PRJEB54813 | food |
| ERS12476546 | PRJEB54813 | food |
| ERS12476610 | PRJEB54813 | food |
| ERS12476611 | PRJEB54813 | food |
| ERS12476612 | PRJEB54813 | food |
| ERS12476613 | PRJEB54813 | food |
| ERS12476547 | PRJEB54813 | food |
| ERS12476614 | PRJEB54813 | food |
| ERS12476548 | PRJEB54813 | food |
| ERS12476615 | PRJEB54813 | food |
| ERS12476618 | PRJEB54813 | food |
| ERS12476617 | PRJEB54813 | food |
| ERS12476616 | PRJEB54813 | food |
| ERS12476619 | PRJEB54813 | food |
| ERS12476549 | PRJEB54813 | food |
| ERS12476620 | PRJEB54813 | food |
| ERS12476677 | PRJEB54813 | food |
| ERS12476621 | PRJEB54813 | food |
| ERS12476719 | PRJEB54813 | food |
| ERS12476551 | PRJEB54813 | food |
| ERS12476550 | PRJEB54813 | food |
| ERS12476622 | PRJEB54813 | food |
| ERS12476624 | PRJEB54813 | food |
| ERS12476623 | PRJEB54813 | food |
| ERS12476625 | PRJEB54813 | food |
| ERS12476697 | PRJEB54813 | food |
| ERS12476626 | PRJEB54813 | food |
| ERS12476627 | PRJEB54813 | food |
| ERS12476552 | PRJEB54813 | food |
| ERS12476553 | PRJEB54813 | food |
| ERS12476628 | PRJEB54813 | food |
| ERS12476554 | PRJEB54813 | food |
| ERS12476673 | PRJEB54813 | food |
| ERS12476629 | PRJEB54813 | food |
| ERS12476630 | PRJEB54813 | food |
| ERS12476631 | PRJEB54813 | food |
| ERS12476555 | PRJEB54813 | food |
| ERS12476632 | PRJEB54813 | food |
| ERS12476678 | PRJEB54813 | food |
| ERS12476556 | PRJEB54813 | food |
| ERS12476679 | PRJEB54813 | food |
| ERS12476557 | PRJEB54813 | food |
| ERS12476633 | PRJEB54813 | food |
| ERS12476687 | PRJEB54813 | food |
| ERS12476688 | PRJEB54813 | food |
| ERS12476689 | PRJEB54813 | food |
| ERS12476634 | PRJEB54813 | food |
| ERS12476635 | PRJEB54813 | food |
| ERS12476636 | PRJEB54813 | food |
| ERS12476637 | PRJEB54813 | food |
| ERS12476638 | PRJEB54813 | food |
| ERS12476642 | PRJEB54813 | food |
| ERS12476640 | PRJEB54813 | food |
| ERS12476700 | PRJEB54813 | food |
| ERS12476698 | PRJEB54813 | food |
| ERS12476639 | PRJEB54813 | food |
| ERS12476641 | PRJEB54813 | food |
| ERS12476699 | PRJEB54813 | food |
| ERS12476708 | PRJEB54813 | food |
| ERS12476643 | PRJEB54813 | food |
| ERS12476644 | PRJEB54813 | food |
| ERS12476645 | PRJEB54813 | food |
| ERS12476646 | PRJEB54813 | food |
| ERS12476647 | PRJEB54813 | food |
| ERS12476648 | PRJEB54813 | food |
| ERS12476649 | PRJEB54813 | food |
| ERS12476651 | PRJEB54813 | food |
| ERS12476702 | PRJEB54813 | food |
| ERS12476650 | PRJEB54813 | food |
| ERS12476701 | PRJEB54813 | food |
| ERS12476652 | PRJEB54813 | food |
| ERS12476653 | PRJEB54813 | food |
| ERS12476558 | PRJEB54813 | food |
| ERS12476654 | PRJEB54813 | food |
| ERS12476680 | PRJEB54813 | food |
| ERS12476655 | PRJEB54813 | food |
| ERS12476657 | PRJEB54813 | food |
| ERS12476656 | PRJEB54813 | food |
| ERS12476658 | PRJEB54813 | food |
| ERS12476659 | PRJEB54813 | food |
| ERS12476559 | PRJEB54813 | food |
| ERS12476703 | PRJEB54813 | food |
| ERS12476660 | PRJEB54813 | food |
| ERS12476661 | PRJEB54813 | food |
| ERS12476560 | PRJEB54813 | food |
| ERS12476681 | PRJEB54813 | food |
| ERS12476662 | PRJEB54813 | food |
| ERS12476663 | PRJEB54813 | food |
| ERS12476664 | PRJEB54813 | food |
| ERS12476666 | PRJEB54813 | food |
| ERS12476665 | PRJEB54813 | food |
| ERS12476561 | PRJEB54813 | food |
| ERS12476704 | PRJEB54813 | food |
| ERS12476667 | PRJEB54813 | food |
| ERS12476668 | PRJEB54813 | food |
| ERS12476669 | PRJEB54813 | food |
| ERS12476670 | PRJEB54813 | food |
| ERS12476671 | PRJEB54813 | food |
| ERS12476772 | PRJEB54813 | food |
| ERS12476773 | PRJEB54813 | food |
| ERS12476774 | PRJEB54813 | food |
| ERS12476776 | PRJEB54813 | food |
| ERS12476777 | PRJEB54813 | food |
| ERS12476775 | PRJEB54813 | food |
| ERS12476778 | PRJEB54813 | food |
| ERS12476721 | PRJEB54813 | food |
| ERS12476722 | PRJEB54813 | food |
| ERS12476779 | PRJEB54813 | food |
| ERS12476871 | PRJEB54813 | food |
| ERS12476781 | PRJEB54813 | food |
| ERS12476780 | PRJEB54813 | food |
| ERS12476782 | PRJEB54813 | food |
| ERS12476846 | PRJEB54813 | food |
| ERS12476860 | PRJEB54813 | food |
| ERS12476723 | PRJEB54813 | food |
| ERS12476724 | PRJEB54813 | food |
| ERS12476783 | PRJEB54813 | food |
| ERS12476784 | PRJEB54813 | food |
| ERS12476785 | PRJEB54813 | food |
| ERS12476725 | PRJEB54813 | food |
| ERS12476847 | PRJEB54813 | food |
| ERS12476863 | PRJEB54813 | food |
| ERS12476786 | PRJEB54813 | food |
| ERS12476787 | PRJEB54813 | food |
| ERS12476726 | PRJEB54813 | food |
| ERS12476727 | PRJEB54813 | food |
| ERS12476728 | PRJEB54813 | food |
| ERS12476832 | PRJEB54813 | food |
| ERS12476789 | PRJEB54813 | food |
| ERS12476730 | PRJEB54813 | food |
| ERS12476729 | PRJEB54813 | food |
| ERS12476788 | PRJEB54813 | food |
| ERS12476790 | PRJEB54813 | food |
| ERS12476848 | PRJEB54813 | food |
| ERS12476842 | PRJEB54813 | food |
| ERS12476791 | PRJEB54813 | food |
| ERS12476843 | PRJEB54813 | food |
| ERS12476792 | PRJEB54813 | food |
| ERS12476794 | PRJEB54813 | food |
| ERS12476793 | PRJEB54813 | food |
| ERS12476731 | PRJEB54813 | food |
| ERS12476732 | PRJEB54813 | food |
| ERS12476733 | PRJEB54813 | food |
| ERS12476872 | PRJEB54813 | food |
| ERS12476734 | PRJEB54813 | food |
| ERS12476827 | PRJEB54813 | food |
| ERS12476831 | PRJEB54813 | food |
| ERS12476844 | PRJEB54813 | food |
| ERS12476735 | PRJEB54813 | food |
| ERS12476736 | PRJEB54813 | food |
| ERS12476795 | PRJEB54813 | food |
| ERS12476849 | PRJEB54813 | food |
| ERS12476737 | PRJEB54813 | food |
| ERS12476852 | PRJEB54813 | food |
| ERS12476864 | PRJEB54813 | food |
| ERS12476796 | PRJEB54813 | food |
| ERS12476797 | PRJEB54813 | food |
| ERS12476850 | PRJEB54813 | food |
| ERS12476833 | PRJEB54813 | food |
| ERS12476798 | PRJEB54813 | food |
| ERS12476799 | PRJEB54813 | food |
| ERS12476851 | PRJEB54813 | food |
| ERS12476834 | PRJEB54813 | food |
| ERS12476738 | PRJEB54813 | food |
| ERS12476800 | PRJEB54813 | food |
| ERS12476801 | PRJEB54813 | food |
| ERS12476802 | PRJEB54813 | food |
| ERS12476803 | PRJEB54813 | food |
| ERS12476873 | PRJEB54813 | food |
| ERS12476840 | PRJEB54813 | food |
| ERS12476835 | PRJEB54813 | food |
| ERS12476739 | PRJEB54813 | food |
| ERS12476861 | PRJEB54813 | food |
| ERS12476865 | PRJEB54813 | food |
| ERS12476740 | PRJEB54813 | food |
| ERS12476741 | PRJEB54813 | food |
| ERS12476804 | PRJEB54813 | food |
| ERS12476742 | PRJEB54813 | food |
| ERS12476743 | PRJEB54813 | food |
| ERS12476841 | PRJEB54813 | food |
| ERS12476805 | PRJEB54813 | food |
| ERS12476744 | PRJEB54813 | food |
| ERS12476836 | PRJEB54813 | food |
| ERS12476853 | PRJEB54813 | food |
| ERS12476862 | PRJEB54813 | food |
| ERS12476806 | PRJEB54813 | food |
| ERS12476807 | PRJEB54813 | food |
| ERS12476745 | PRJEB54813 | food |
| ERS12476746 | PRJEB54813 | food |
| ERS12476747 | PRJEB54813 | food |
| ERS12476837 | PRJEB54813 | food |
| ERS12476748 | PRJEB54813 | food |
| ERS12476749 | PRJEB54813 | food |
| ERS12476750 | PRJEB54813 | food |
| ERS12476808 | PRJEB54813 | food |
| ERS12476854 | PRJEB54813 | food |
| ERS12476751 | PRJEB54813 | food |
| ERS12476752 | PRJEB54813 | food |
| ERS12476809 | PRJEB54813 | food |
| ERS12476810 | PRJEB54813 | food |
| ERS12476811 | PRJEB54813 | food |
| ERS12476812 | PRJEB54813 | food |
| ERS12476866 | PRJEB54813 | food |
| ERS12476753 | PRJEB54813 | food |
| ERS12476828 | PRJEB54813 | food |
| ERS12476813 | PRJEB54813 | food |
| ERS12476814 | PRJEB54813 | food |
| ERS12476754 | PRJEB54813 | food |
| ERS12476874 | PRJEB54813 | food |
| ERS12476875 | PRJEB54813 | food |
| ERS12476756 | PRJEB54813 | food |
| ERS12476755 | PRJEB54813 | food |
| ERS12476757 | PRJEB54813 | food |
| ERS12476758 | PRJEB54813 | food |
| ERS12476815 | PRJEB54813 | food |
| ERS12476838 | PRJEB54813 | food |
| ERS12476829 | PRJEB54813 | food |
| ERS12476816 | PRJEB54813 | food |
| ERS12476867 | PRJEB54813 | food |
| ERS12476876 | PRJEB54813 | food |
| ERS12476760 | PRJEB54813 | food |
| ERS12476759 | PRJEB54813 | food |
| ERS12476855 | PRJEB54813 | food |
| ERS12476761 | PRJEB54813 | food |
| ERS12476817 | PRJEB54813 | food |
| ERS12476856 | PRJEB54813 | food |
| ERS12476764 | PRJEB54813 | food |
| ERS12476818 | PRJEB54813 | food |
| ERS12476763 | PRJEB54813 | food |
| ERS12476762 | PRJEB54813 | food |
| ERS12476869 | PRJEB54813 | food |
| ERS12476868 | PRJEB54813 | food |
| ERS12476870 | PRJEB54813 | food |
| ERS12476819 | PRJEB54813 | food |
| ERS12476845 | PRJEB54813 | food |
| ERS12476839 | PRJEB54813 | food |
| ERS12476877 | PRJEB54813 | food |
| ERS12476857 | PRJEB54813 | food |
| ERS12476830 | PRJEB54813 | food |
| ERS12476820 | PRJEB54813 | food |
| ERS12476765 | PRJEB54813 | food |
| ERS12476821 | PRJEB54813 | food |
| ERS12476822 | PRJEB54813 | food |
| ERS12476766 | PRJEB54813 | food |
| ERS12476767 | PRJEB54813 | food |
| ERS12476823 | PRJEB54813 | food |
| ERS12476824 | PRJEB54813 | food |
| ERS12476825 | PRJEB54813 | food |
| ERS12476768 | PRJEB54813 | food |
| ERS12476769 | PRJEB54813 | food |
| ERS12476770 | PRJEB54813 | food |
| ERS12476858 | PRJEB54813 | food |
| ERS12476859 | PRJEB54813 | food |
| ERS12476826 | PRJEB54813 | food |
| ERS12476771 | PRJEB54813 | food |
| ERS12476921 | PRJEB54813 | food |
| ERS12476922 | PRJEB54813 | food |
| ERS12476878 | PRJEB54813 | food |
| ERS12476966 | PRJEB54813 | food |
| ERS12476879 | PRJEB54813 | food |
| ERS12476923 | PRJEB54813 | food |
| ERS12476881 | PRJEB54813 | food |
| ERS12476880 | PRJEB54813 | food |
| ERS12476882 | PRJEB54813 | food |
| ERS12476976 | PRJEB54813 | food |
| ERS12476883 | PRJEB54813 | food |
| ERS12476884 | PRJEB54813 | food |
| ERS12476924 | PRJEB54813 | food |
| ERS12476972 | PRJEB54813 | food |
| ERS12476925 | PRJEB54813 | food |
| ERS12476926 | PRJEB54813 | food |
| ERS12476885 | PRJEB54813 | food |
| ERS12476886 | PRJEB54813 | food |
| ERS12476887 | PRJEB54813 | food |
| ERS12476996 | PRJEB54813 | food |
| ERS12476973 | PRJEB54813 | food |
| ERS12476977 | PRJEB54813 | food |
| ERS12476888 | PRJEB54813 | food |
| ERS12476978 | PRJEB54813 | food |
| ERS12476890 | PRJEB54813 | food |
| ERS12476889 | PRJEB54813 | food |
| ERS12476891 | PRJEB54813 | food |
| ERS12476979 | PRJEB54813 | food |
| ERS12476892 | PRJEB54813 | food |
| ERS12476893 | PRJEB54813 | food |
| ERS12476980 | PRJEB54813 | food |
| ERS12476894 | PRJEB54813 | food |
| ERS12476981 | PRJEB54813 | food |
| ERS12476982 | PRJEB54813 | food |
| ERS12476895 | PRJEB54813 | food |
| ERS12476897 | PRJEB54813 | food |
| ERS12476896 | PRJEB54813 | food |
| ERS12476997 | PRJEB54813 | food |
| ERS12477009 | PRJEB54813 | food |
| ERS12476927 | PRJEB54813 | food |
| ERS12476898 | PRJEB54813 | food |
| ERS12476929 | PRJEB54813 | food |
| ERS12476930 | PRJEB54813 | food |
| ERS12476928 | PRJEB54813 | food |
| ERS12476998 | PRJEB54813 | food |
| ERS12476984 | PRJEB54813 | food |
| ERS12476983 | PRJEB54813 | food |
| ERS12476931 | PRJEB54813 | food |
| ERS12476967 | PRJEB54813 | food |
| ERS12476932 | PRJEB54813 | food |
| ERS12476899 | PRJEB54813 | food |
| ERS12476974 | PRJEB54813 | food |
| ERS12476999 | PRJEB54813 | food |
| ERS12476900 | PRJEB54813 | food |
| ERS12476933 | PRJEB54813 | food |
| ERS12476901 | PRJEB54813 | food |
| ERS12477001 | PRJEB54813 | food |
| ERS12477000 | PRJEB54813 | food |
| ERS12476968 | PRJEB54813 | food |
| ERS12476902 | PRJEB54813 | food |
| ERS12476985 | PRJEB54813 | food |
| ERS12476934 | PRJEB54813 | food |
| ERS12476935 | PRJEB54813 | food |
| ERS12477002 | PRJEB54813 | food |
| ERS12476936 | PRJEB54813 | food |
| ERS12476904 | PRJEB54813 | food |
| ERS12476903 | PRJEB54813 | food |
| ERS12476937 | PRJEB54813 | food |
| ERS12477008 | PRJEB54813 | food |
| ERS12476987 | PRJEB54813 | food |
| ERS12476986 | PRJEB54813 | food |
| ERS12476905 | PRJEB54813 | food |
| ERS12476989 | PRJEB54813 | food |
| ERS12476990 | PRJEB54813 | food |
| ERS12476938 | PRJEB54813 | food |
| ERS12476988 | PRJEB54813 | food |
| ERS12476939 | PRJEB54813 | food |
| ERS12476906 | PRJEB54813 | food |
| ERS12477010 | PRJEB54813 | food |
| ERS12476907 | PRJEB54813 | food |
| ERS12476940 | PRJEB54813 | food |
| ERS12476969 | PRJEB54813 | food |
| ERS12476941 | PRJEB54813 | food |
| ERS12476943 | PRJEB54813 | food |
| ERS12476942 | PRJEB54813 | food |
| ERS12477011 | PRJEB54813 | food |
| ERS12476908 | PRJEB54813 | food |
| ERS12476909 | PRJEB54813 | food |
| ERS12477012 | PRJEB54813 | food |
| ERS12476944 | PRJEB54813 | food |
| ERS12476911 | PRJEB54813 | food |
| ERS12476910 | PRJEB54813 | food |
| ERS12476945 | PRJEB54813 | food |
| ERS12476946 | PRJEB54813 | food |
| ERS12476947 | PRJEB54813 | food |
| ERS12476948 | PRJEB54813 | food |
| ERS12476991 | PRJEB54813 | food |
| ERS12477003 | PRJEB54813 | food |
| ERS12476949 | PRJEB54813 | food |
| ERS12476992 | PRJEB54813 | food |
| ERS12476951 | PRJEB54813 | food |
| ERS12476950 | PRJEB54813 | food |
| ERS12477004 | PRJEB54813 | food |
| ERS12476952 | PRJEB54813 | food |
| ERS12476953 | PRJEB54813 | food |
| ERS12476912 | PRJEB54813 | food |
| ERS12476970 | PRJEB54813 | food |
| ERS12477005 | PRJEB54813 | food |
| ERS12476914 | PRJEB54813 | food |
| ERS12476913 | PRJEB54813 | food |
| ERS12476954 | PRJEB54813 | food |
| ERS12476956 | PRJEB54813 | food |
| ERS12476957 | PRJEB54813 | food |
| ERS12476955 | PRJEB54813 | food |
| ERS12476994 | PRJEB54813 | food |
| ERS12476959 | PRJEB54813 | food |
| ERS12476993 | PRJEB54813 | food |
| ERS12477006 | PRJEB54813 | food |
| ERS12476958 | PRJEB54813 | food |
| ERS12476915 | PRJEB54813 | food |
| ERS12476960 | PRJEB54813 | food |
| ERS12476917 | PRJEB54813 | food |
| ERS12476916 | PRJEB54813 | food |
| ERS12476971 | PRJEB54813 | food |
| ERS12476918 | PRJEB54813 | food |
| ERS12476961 | PRJEB54813 | food |
| ERS12476964 | PRJEB54813 | food |
| ERS12476963 | PRJEB54813 | food |
| ERS12476962 | PRJEB54813 | food |
| ERS12476975 | PRJEB54813 | food |
| ERS12476995 | PRJEB54813 | food |
| ERS12476919 | PRJEB54813 | food |
| ERS12476920 | PRJEB54813 | food |
| ERS12476965 | PRJEB54813 | food |
| ERS12477013 | PRJEB54813 | food |
| ERS12477007 | PRJEB54813 | food |
| ERS12477049 | PRJEB54813 | food |
| ERS12477015 | PRJEB54813 | food |
| ERS12477048 | PRJEB54813 | food |
| ERS12477014 | PRJEB54813 | food |
| ERS12477082 | PRJEB54813 | food |
| ERS12477016 | PRJEB54813 | food |
| ERS12477096 | PRJEB54813 | food |
| ERS12477050 | PRJEB54813 | food |
| ERS12477017 | PRJEB54813 | food |
| ERS12477122 | PRJEB54813 | food |
| ERS12477051 | PRJEB54813 | food |
| ERS12477020 | PRJEB54813 | food |
| ERS12477019 | PRJEB54813 | food |
| ERS12477018 | PRJEB54813 | food |
| ERS12477101 | PRJEB54813 | food |
| ERS12477098 | PRJEB54813 | food |
| ERS12477097 | PRJEB54813 | food |
| ERS12477114 | PRJEB54813 | food |
| ERS12477140 | PRJEB54813 | food |
| ERS12477123 | PRJEB54813 | food |
| ERS12477052 | PRJEB54813 | food |
| ERS12477053 | PRJEB54813 | food |
| ERS12477144 | PRJEB54813 | food |
| ERS12477145 | PRJEB54813 | food |
| ERS12477084 | PRJEB54813 | food |
| ERS12477055 | PRJEB54813 | food |
| ERS12477054 | PRJEB54813 | food |
| ERS12477115 | PRJEB54813 | food |
| ERS12477102 | PRJEB54813 | food |
| ERS12477021 | PRJEB54813 | food |
| ERS12477022 | PRJEB54813 | food |
| ERS12477090 | PRJEB54813 | food |
| ERS12477023 | PRJEB54813 | food |
| ERS12477099 | PRJEB54813 | food |
| ERS12477024 | PRJEB54813 | food |
| ERS12477025 | PRJEB54813 | food |
| ERS12477026 | PRJEB54813 | food |
| ERS12477103 | PRJEB54813 | food |
| ERS12477056 | PRJEB54813 | food |
| ERS12477104 | PRJEB54813 | food |
| ERS12477027 | PRJEB54813 | food |
| ERS12477028 | PRJEB54813 | food |
| ERS12477124 | PRJEB54813 | food |
| ERS12477029 | PRJEB54813 | food |
| ERS12477138 | PRJEB54813 | food |
| ERS12477030 | PRJEB54813 | food |
| ERS12477125 | PRJEB54813 | food |
| ERS12477057 | PRJEB54813 | food |
| ERS12477031 | PRJEB54813 | food |
| ERS12477032 | PRJEB54813 | food |
| ERS12477034 | PRJEB54813 | food |
| ERS12477058 | PRJEB54813 | food |
| ERS12477033 | PRJEB54813 | food |
| ERS12477126 | PRJEB54813 | food |
| ERS12477059 | PRJEB54813 | food |
| ERS12477091 | PRJEB54813 | food |
| ERS12477130 | PRJEB54813 | food |
| ERS12477128 | PRJEB54813 | food |
| ERS12477127 | PRJEB54813 | food |
| ERS12477061 | PRJEB54813 | food |
| ERS12477105 | PRJEB54813 | food |
| ERS12477060 | PRJEB54813 | food |
| ERS12477062 | PRJEB54813 | food |
| ERS12477133 | PRJEB54813 | food |
| ERS12477129 | PRJEB54813 | food |
| ERS12477035 | PRJEB54813 | food |
| ERS12477119 | PRJEB54813 | food |
| ERS12477120 | PRJEB54813 | food |
| ERS12477063 | PRJEB54813 | food |
| ERS12477036 | PRJEB54813 | food |
| ERS12477131 | PRJEB54813 | food |
| ERS12477132 | PRJEB54813 | food |
| ERS12477134 | PRJEB54813 | food |
| ERS12477064 | PRJEB54813 | food |
| ERS12477037 | PRJEB54813 | food |
| ERS12477106 | PRJEB54813 | food |
| ERS12477092 | PRJEB54813 | food |
| ERS12477121 | PRJEB54813 | food |
| ERS12477067 | PRJEB54813 | food |
| ERS12477066 | PRJEB54813 | food |
| ERS12477065 | PRJEB54813 | food |
| ERS12477139 | PRJEB54813 | food |
| ERS12477038 | PRJEB54813 | food |
| ERS12477095 | PRJEB54813 | food |
| ERS12477094 | PRJEB54813 | food |
| ERS12477085 | PRJEB54813 | food |
| ERS12477068 | PRJEB54813 | food |
| ERS12477039 | PRJEB54813 | food |
| ERS12477069 | PRJEB54813 | food |
| ERS12477086 | PRJEB54813 | food |
| ERS12477135 | PRJEB54813 | food |
| ERS12477040 | PRJEB54813 | food |
| ERS12477093 | PRJEB54813 | food |
| ERS12477136 | PRJEB54813 | food |
| ERS12477141 | PRJEB54813 | food |
| ERS12477107 | PRJEB54813 | food |
| ERS12477108 | PRJEB54813 | food |
| ERS12477070 | PRJEB54813 | food |
| ERS12477087 | PRJEB54813 | food |
| ERS12477071 | PRJEB54813 | food |
| ERS12477116 | PRJEB54813 | food |
| ERS12477041 | PRJEB54813 | food |
| ERS12477109 | PRJEB54813 | food |
| ERS12477110 | PRJEB54813 | food |
| ERS12477072 | PRJEB54813 | food |
| ERS12477042 | PRJEB54813 | food |
| ERS12477043 | PRJEB54813 | food |
| ERS12477100 | PRJEB54813 | food |
| ERS12477111 | PRJEB54813 | food |
| ERS12477112 | PRJEB54813 | food |
| ERS12477143 | PRJEB54813 | food |
| ERS12477142 | PRJEB54813 | food |
| ERS12477083 | PRJEB54813 | food |
| ERS12477044 | PRJEB54813 | food |
| ERS12477045 | PRJEB54813 | food |
| ERS12477146 | PRJEB54813 | food |
| ERS12477117 | PRJEB54813 | food |
| ERS12477088 | PRJEB54813 | food |
| ERS12477089 | PRJEB54813 | food |
| ERS12477073 | PRJEB54813 | food |
| ERS12477074 | PRJEB54813 | food |
| ERS12477077 | PRJEB54813 | food |
| ERS12477046 | PRJEB54813 | food |
| ERS12477076 | PRJEB54813 | food |
| ERS12477075 | PRJEB54813 | food |
| ERS12477137 | PRJEB54813 | food |
| ERS12477113 | PRJEB54813 | food |
| ERS12477047 | PRJEB54813 | food |
| ERS12477148 | PRJEB54813 | food |
| ERS12477079 | PRJEB54813 | food |
| ERS12477078 | PRJEB54813 | food |
| ERS12477118 | PRJEB54813 | food |
| ERS12477080 | PRJEB54813 | food |
| ERS12477147 | PRJEB54813 | food |
| ERS12477081 | PRJEB54813 | food |

### Supplementary figures


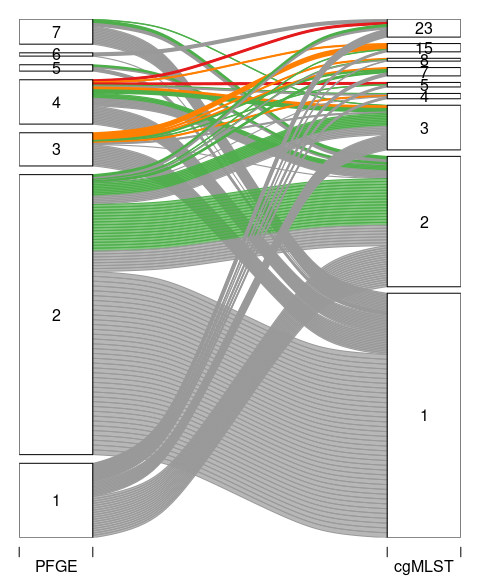


**Supplementary Figure 1**: Correspondence of clusters between the two typing methods: PFGE with ApaI/AscI, and cgMLST. The lateral bars stand for each of the two methods. The height of the lateral bars indicate the number of sequences comprised in clusters, and the size of these clusters is indicated by the numbers in each block. e.g. for PFGE a large proportion of the sequences clustered in clusters of size two, while only a small number of sequences clustered in a cluster of six. The colour of the alluvials indicate sequences that are found together in a cluster in both methods: grey=one sequence, green=two sequences, orange=three sequences, red=four sequences e.g. a large fraction of the sequences will be found in a same cluster in both methods with only one other sequence (pairs of two).


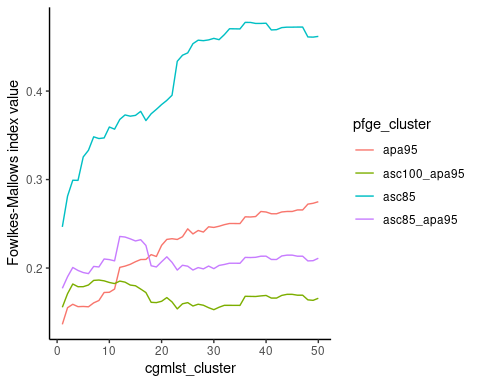


**Supplementary Figure 2**: Concordance among the cgMLST and PFGE clusters, as measured by the value of the FM index (y-axis), for the various restriction enzymes profiles in PFGE (colours) and distance thresholds for defining cgMLST clusters (x-axis).


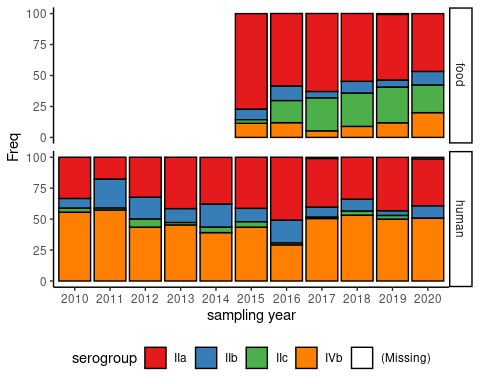


**Supplementary Figure 3**: Distribution of serogroups per year among the human and food *Lm* sequences.


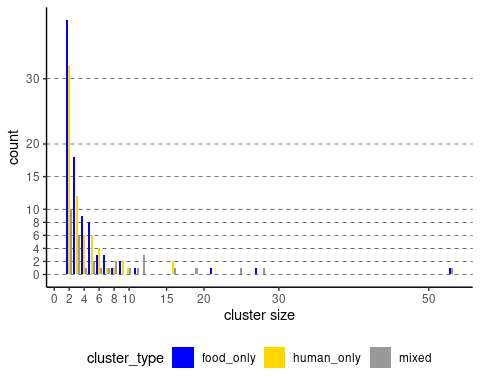


**Supplementary Figure 4**: Cluster size distribution.


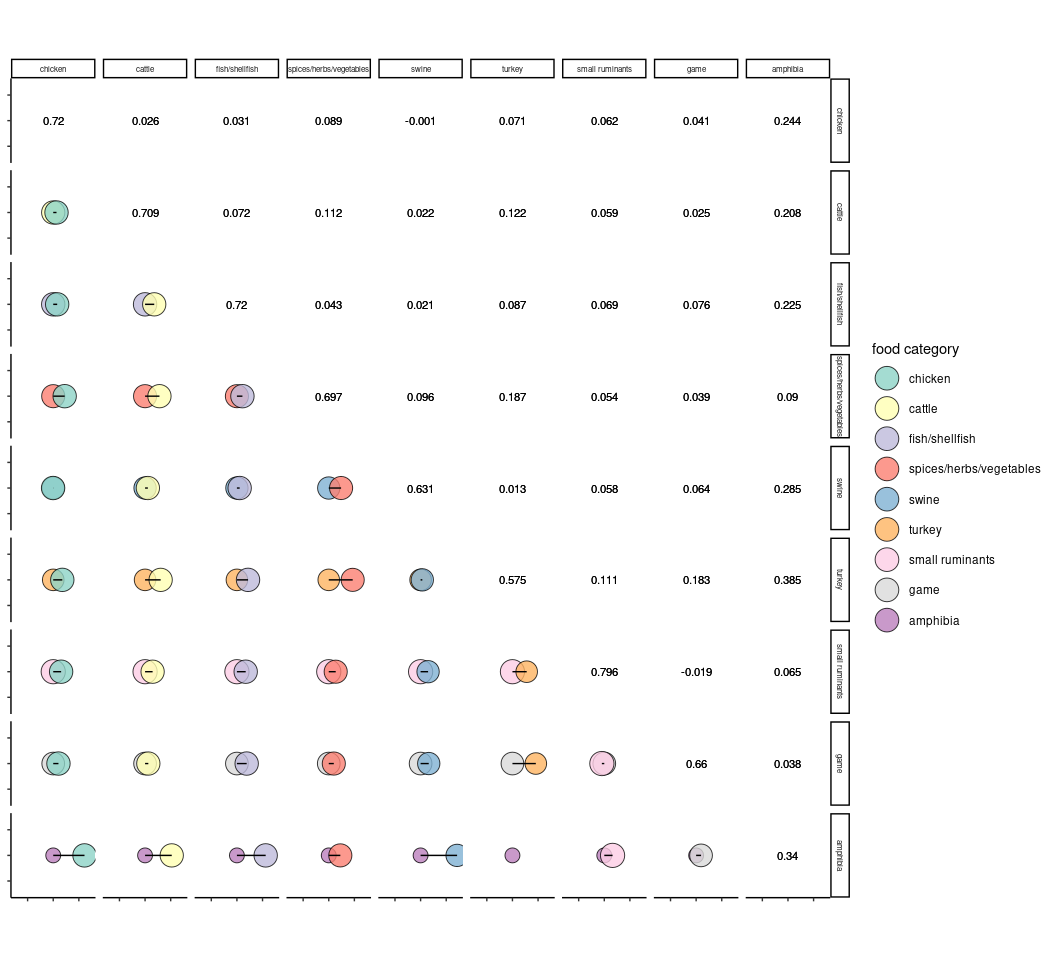


**Supplementary Figure 5**: Genetic differentiation of *Lm* in the populations defined by the food categories. The upper half of the graph matrix shows the pairwise differentiation indices between the food categories indicated in each panel headers; the major diagonal indicates the genetic divergence within a food category; the lower matrix shows a visual representation of the above mentioned indices, where the diameters of the circles are proportional to the within population differentiation indices and the distance between their centers is proportional to the pairwise differentiation index (between). A larger overlap of the circles is indicative of lower genetic differentiation of the respective populations.


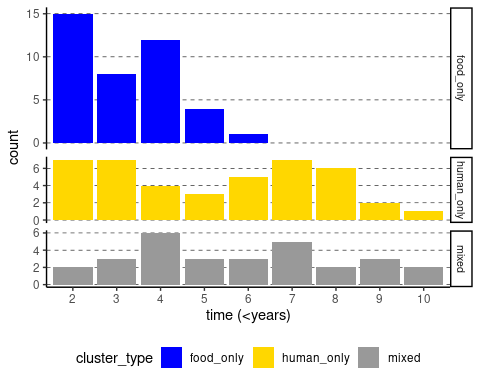


**Supplementary Figure 6**: Distribution of clusters according to their persistence. The x-axis indicates the maximum interval of years the clusters have been found; the y-axis indicates the number of clusters.
